# A resting-state fMRI pattern of spinocerebellar ataxia type 3 and comparison with ^18^F-FDG PET

**DOI:** 10.1016/j.nicl.2022.103023

**Published:** 2022-04-25

**Authors:** Harm J. van der Horn, Sanne K. Meles, Jelmer G. Kok, Victor M. Vergara, Shile Qi, Vince D. Calhoun, Jelle R. Dalenberg, Jeroen C.W. Siero, Remco J. Renken, Jeroen J. de Vries, Jacoba M. Spikman, Hubertus P.H. Kremer, Bauke M. De Jong

**Affiliations:** a Department of Neurology, University Medical Center Groningen, University of Groningen, the Netherlands; b Tri-institutional Center for Translational Research (TReNDS), Georgia State, Georgia Tech, Emory, Atlanta, GA, USA; c Department of Radiology, Utrecht Center for Image Sciences, University Medical Center Utrecht, Utrecht, the Netherlands; d Department of Neuroscience, University Medical Center Groningen, University of Groningen, the Netherlands; e Department of Neuropsychology, University of Groningen, University Medical Center Groningen, Groningen, the Netherlands; f Spinoza Centre for Neuroimaging Amsterdam, Amsterdam, the Netherlands

**Keywords:** BOLD, Ataxia, ICA, Brain glucose metabolism, Disease-related pattern

## Abstract

Spinocerebellar ataxia type 3 (SCA3) is a rare genetic neurodegenerative disease. The neurobiological basis of SCA3 is still poorly understood, and up until now resting-state fMRI (rs-fMRI) has not been used to study this disease. In the current study we investigated (multi-echo) rs-fMRI data from patients with genetically confirmed SCA3 (n = 17) and matched healthy subjects (n = 16). Using independent component analysis (ICA) and subsequent regression with bootstrap resampling, we identified a pattern of differences between patients and healthy subjects, which we coined the fMRI SCA3 related pattern (fSCA3-RP) comprising cerebellum, anterior striatum and various cortical regions. Individual fSCA3-RP scores were highly correlated with a previously published ^18^F-FDG PET pattern found in the same sample (rho = 0.78, P = 0.0003). Also, a high correlation was found with the Scale for Assessment and Rating of Ataxia scores (r = 0.63, P = 0.007). No correlations were found with neuropsychological test scores, nor with levels of grey matter atrophy. Compared with the ^18^F-FDG PET pattern, the fSCA3-RP included a more extensive contribution of the mediofrontal cortex, putatively representing changes in default network activity. This rs-fMRI identification of additional regions is proposed to reflect a consequence of the nature of the BOLD technique, enabling measurement of dynamic network activity, compared to the more static ^18^F-FDG PET methodology. Altogether, our findings shed new light on the neural substrate of SCA3, and encourage further validation of the fSCA3-RP to assess its potential contribution as imaging biomarker for future research and clinical use.

## Highlights

• This is the first study identifying a resting-state fMRI pattern in SCA3.
• This pattern was closely associated with a metabolic (^18^F-FDG PET) counterpart.
• Pattern subject scores were highly correlated with ataxia severity.

## 1 Introduction

Spinocerebellar ataxia type 3 (SCA3) is a rare neurodegenerative disease caused by a trinucleotide (CAG) repeat expansion in exon 10 of the ATXN3 gene on chromosome 14 (p32). Neuropathological studies in SCA3 have revealed variable neuronal loss in cerebellum, brainstem, thalamus, subthalamic nucleus, pallidum, and motor cortex (Rüb et al., 2008, Rüb et al., 2013, Seidel et al., 2012). The cerebellum is most severely affected, with ataxia as the presenting and most prominent feature, but patients may also develop pyramidal and extra-pyramidal signs, neuropathy, oculomotor dysfunction, and cognitive problems (Pilotto and Saxena, 2018, Ruano et al., 2014, Yap et al., 2021). More than direct effects of local pathology, clinical manifestations are likely to be a consequence of more widespread functional changes in cerebellar-thalamo-cerebral and striatal-cortical networks. In-vivo insights into brain networks involved in SCA3 can be provided with functional neuroimaging combined with advanced computational algorithms. The main principle of functional neuro-imaging is that brain activity can be mapped by measuring energy metabolism or hemodynamics, (indirectly) reflecting the underlying cellular events. In the present study we employed Functional Magnetic Resonance Imaging (fMRI) to measure resting-state regional cerebral hemodynamics in order to identify SCA3-related network impairment. Moreover, we compared these results with functional network changes recently identified with metabolic measurements in the same patient group (Meles et al., 2018), which provides insight in the overlap and possible differences that might be expected from the two methods of data acquisition.

Local glucose metabolism and oxygen utilization are coupled with local brain activity, which implies that in vivo measurement of regional glucose consumption with ^18^F-2-fluoro-2-deoxy-D-glucose Positron Emission Tomography (^18^F-FDG PET) provides an index of regional neuronal activity (Reivich et al., 1979). Under physiological steady-state conditions, cerebral blood flow (CBF) is tightly coupled to the level of cerebral oxygen and glucose consumption (Sokoloff, 1977). Studies of brain metabolism with ^18^F-FDG PET are typically static and capture accumulation of the ^18^F-FDG tracer in brain tissue during the uptake and scanning periods of around 30 and 5 min, respectively. Brain regions with altered ^18^F-FDG uptake can be identified in patients as compared with controls using univariate models. Regions with decreased ^18^F-FDG uptake may reflect (i) impaired neuronal function due to localized pathology, but (ii) also neuronal dysfunction in unaffected tissue, secondarily caused by dysfunction of a distant region, if these two regions are organized in the same functional brain network (e.g., Reesink et al., 2018).

Spatial covariance analysis of ^18^F-FDG PET data is designed to take into account the functional relationships between brain regions. In this, principal component analysis (PCA) can be used to reduce the large number of voxels for every subject to a limited number of orthogonal dimensions (eigenvectors) that explain the major sources of variance in the data. A disease-related pattern (or ‘network’) is identified among the eigenvectors that discriminate between controls and patients (Eidelberg, 2009). Although spatial covariance analysis provides a better approximation of network-level effects on brain metabolism than classic univariate approaches, spatial covariance patterns do not reflect true functional connectivity.

During an increase of local brain activity, regional CBF exceeds oxygen extraction, resulting in a relative decrease in the oxygen extraction fraction (Iadecola and Nedergaard, 2007, Raichle et al., 2001). The subsequent increase of oxygenated hemoglobin can be detected with fMRI, a measurement which is coined is called the blood oxygenation level-dependent (BOLD) response (Logothetis et al., 2001, Ogawa et al., 1990). BOLD fMRI provides a time-series of fluctuations in the BOLD signal for each voxel, which is a reflection of fluctuations in CBF caused by changes in neuronal activity. Synchronization of BOLD fluctuations across regions implies that these regions are functionally connected and participate in the same brain network. Loss of integrity (i.e., synchronization) of one of the participating regions will affect the entire network. With independent component analysis (ICA), functional connectivity networks can be identified. This method enables separation of a mixture of sources and noise into independent components (i.e., (parts of) brain networks) and thus is very suitable for detecting pathophysiological changes in specific brain networks (Calhoun et al., 2001).

As regional CBF and glucose metabolism are both related to local neuronal activity, fMRI and ^18^F-FDG PET are expected to quantify signals from a similar source. However, from the above it also follows that ^18^F-FDG PET patterns reflect the *spatial* covariance relationships between voxels across subjects, whereas fMRI-ICA also delineates relationships between voxels *over time*. As a consequence, certain regions may show altered temporal fluctuations, but normal FDG uptake in a static situation. Thus, BOLD fMRI potentially gives additional information concerning dynamical aspects of functional network changes.

Until now, only two fMRI-studies have been conducted in SCA3, both of which made use of a motor activation paradigm (Duarte et al., 2016, Stefanescu et al., 2015, Wan et al., 2020). These studies have demonstrated changes in activation of the cerebellar cortex and nuclei, basal ganglia, thalamus and cerebral cortex. These regional changes were more widely distributed than regional atrophy. In the present fMRI study, we used the BOLD technique for scanning in resting state, which can be expected to yield valuable information about coherent time-variant aspects of spatially distributed brain function. So far, no resting-state fMRI studies on SCA3 have been published.

In a previous study, we identified a disease-related cerebral metabolic pattern in ^18^F-FDG-PET scans of SCA3 patients and age-matched controls using PCA (Meles et al., 2018). This SCA3 related pattern (pSCA3-RP) was characterized by relative decreases in the cerebellum, brainstem, caudate nucleus and posterior parietal cortex, co-varying with relatively increased activity in several limbic regions and the somatosensory cortex. An advantage of the PCA approach is that ^18^F-FDG PET brain pattern expression can be quantified in individual scans. The degree of pattern expression is denoted by a z-score. In our previous study, pSCA3-RP z-scores were significantly correlated with the severity of ataxia as measured by the Scale for Assessment and Rating of Ataxia (SARA). However, SARA scores did not correlate with ^18^F-FDG uptake in any single region, supporting the notion that the SCA3-RP represents network-level changes underlying ataxia in SCA3.

Integrating fMRI and ^18^F-FDG PET may provide new and complementary insights in the underlying network changes in SCA3. In this study, we aimed to investigate the changes in resting-state brain networks in SCA3 using fMRI combined with ICA. Although the ICA approach has had limited applicability to quantify scans at an individual basis, an adapted ICA approach, analogous to the ^18^F-FDG PET PCA analysis, has been shown to enable identification of resting state fMRI networks and quantification of their activity in individual cases (Vo et al., 2017). Using this approach, we set out to identify independent components (ICs) that reflect the main neural changes underlying SCA3, and that could potentially be used to quantify disease-related changes on a scan-by-scan basis, analogous to the previously identified SCA3-related ^18^F-FDG PET pattern in the same dataset (Meles et al., 2018). We examined whether individual pattern scores on the resting state fMRI SCA3-related pattern (fSCA3-RP) are linked to (i) the ^18^F-FDG PET pattern scores, (ii) patterns of grey matter atrophy, and (iii) clinical measures, such as ataxia severity and cognitive functioning.

## 2 Materials and methods

### 2.1 Participants and clinical measures

A group of 17 patients with SCA3 and a group of 16 age-, sex-, and education-matched healthy controls were included from a previous study (for details, see (Meles et al., 2018)). Onset age of SCA3 was estimated after a review of each patient’s medical chart, as the age at which the patient first reported symptoms to the treating neurologist (most often this included gait problems). For both groups, the severity of ataxia was assessed by an experienced neurologist (H.P.H.K., or J.J.d.V.) using the Scale for Assessment and Rating of Ataxia (SARA) (Schmitz-Hübsch et al., 2006). Anxiety and depression was measured using the hospital anxiety and depression scale (HADS) (Zigmond & Snaith, 1983). Neuropsychological metrics included semantic and letter fluency tests to measure language and executive functioning, the Dutch version of the Rey Auditory Verbal Learning Test (RAVLT) to measure memory, and the Symbol Digit Modalities Test (SDMT) to measure mental processing speed (Rey, 1964, Smith, 2007). The selection of these tests was based on previous publications on cognitive functioning in SCA3 (Braga-Neto et al., 2012, Braga-Neto et al., 2014). Raw scores were used for statistical analyses.

The study was approved by the Medical Ethics Committee of the University Medical Center Groningen, The Netherlands, and all subjects gave written informed consent (NL45036.042.13). All procedures were carried out in accordance with the Declaration of Helsinki.

### 2.2 Image acquisition and preprocessing

**Fig. 1:**
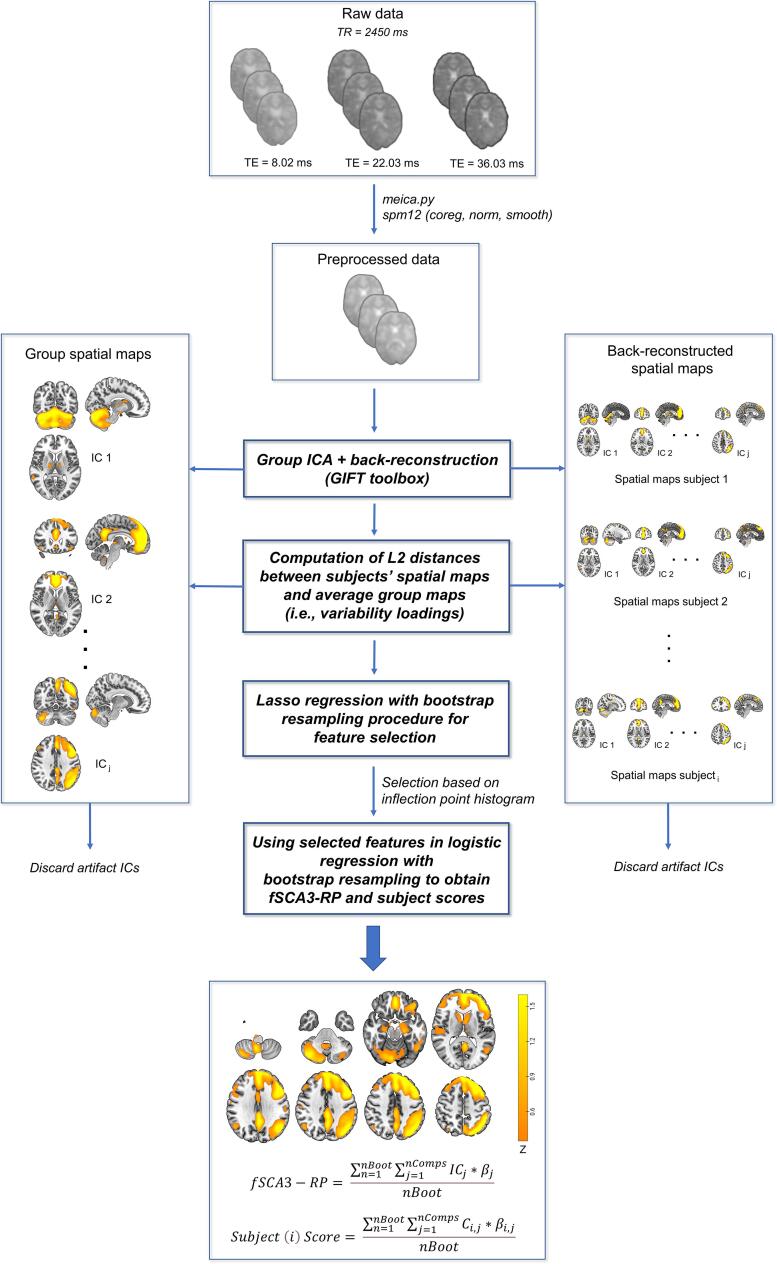
Resting-state fMRI analysis pipeline. Preprocessed fMRI data was subject to a group independent component analysis (GICA). Euclidean Distance (L2) variability loadings ($C_{i,j}$) were computed, and Bootstrapped Feature Selection via Lasso Regression was applied. After selecting the most robust predictors using the frequency histogram, final model estimates were calculated using Bootstrapped Logistic Regression. During each bootstrap iteration (n of nBoot), the components (${IC}_{1\;\cdots \;nComps}$) of the final model were linearly combined in a new spatial map (consisting of IC19, 22, and 10 out of nComps). The fSCA3 related pattern was computed by taking the mean (spatial map) across all bootstrap iterations (nBoot = 5,000). Accordingly, subject scores were calculated by taking the mean of the linearly combined variability loadings ($C_{i,j}$) obtained during each iteration.

Fig. 1 depicts the analysis pipeline. The details on the acquisition parameters and fMRI preprocessing pipeline can be found in our previously published fMRI-study performed by the Department of Neuroscience of the University Medical Center Groningen (Dalenberg et al., 2018). In brief, 300 resting-state (eyes closed) functional brain images were recorded using a multi-echo sequence (FOV 224 × 224 × 157.5 mm^3^ (rl, ap, fh); 45 axial slices; voxel size 3.5 × 3.5 × 3.5 mm; matrix size 64 × 61; slice gap 0 mm; echo times 8.02, 22.03, and 36.03 ms; flip angle 80°; SENSE factors: 3, 1 (ap, os); repetition time 2.45 s; descending slice acquisition). In addition, a 3D T1-weighted image was acquired for anatomical reference, and analysis of grey matter atrophy. Functional data were denoised at the subject-level using the *meica.py* pipeline, consisting of realignment, slice timing correction, TE-dependent ICA, T2*-weighted time course combination (Kundu et al., 2012, Kundu et al., 2013). This was followed by co-registration to the T1-weighted volume, normalization and smoothing (6 mm FWHM) using SPM12 (Wellcome Department, University College London, England) implemented in MATLAB version 2020a (MathWorks, Natick, Massachusetts, USA). Detailed information regarding acquisition parameters and preprocessing of T1 (resulting in smoothed grey matter segmentations), and [^18^F]-FDG PET images, as well as the generation of a metabolic pattern (pSCA3-RP) using the scaled sub-profile model (SSM) PCA method, can be found in our previously published work on the same sample (Meles et al., 2018). For 14/17 patients with SCA3, and 10/16 healthy subjects fMRI and [^18^F]-FDG PET acquisition was done on the same day.

### 2.3 Independent component analysis

The group ICA of fMRI toolbox (GIFT; https://trendscenter.org/software/gift), implemented in MATLAB, was used for ICA (Calhoun et al., 2001). The first three volumes were discarded to ensure signal equilibrium. The number of independent components was estimated using the minimum description length (MDL) with a smoothness kernel of 6 mm at FWHM. For fMRI the optimal number of ICs was 34. Group ICA (Infomax algorithm) was then run multiple times (20 iterations) using ICASSO, and the best estimate (centrotype of cluster) for every component was selected (Himberg et al., 2004). This ultimately resulted in a set of group ICs (each consisting of a spatial map and a time course). Subject-specific ICs were generated based on linear back-reconstruction with scaling to z-scores. Voxel z-scores of an IC can be either positive or negative, and express how strongly voxels are correlated (for positive values) or anti-correlated (for negative values) with the IC time course (or in other words, values reflect relative functional connectivity of a voxel within a specific IC). In the GIFT output, positive voxels of an IC are larger in amplitude, and if necessary, the sign of an IC is flipped to ensure this will happen.

Prior to further analyses, individual ICs (maps, time courses and power spectra) were inspected independently by two raters (H.J.v.d.H. and S.K.M.) and discussed until consensus was reached regarding the neural or artefactual nature of components. This resulted in a set of 23 retained independent neural components, and 11 non-neural components that were discarded (all ICs, and their corresponding power spectra, are shown in Suppl. Material 1). Subsequently, a matrix called $C_{i,j}$, containing IC variability loadings for every subject *i* and component *j*, was generated by calculating the squared Euclidean (L2) distances between subjects’ spatial maps and the group (average) maps (Qi et al., 2019). These variability loadings reflect how spatially different subjects’ ICs are from the average group components.

### 2.4 Identification of the fSCA3-related pattern

We identified a fSCA3-RP using a two stage bootstrap feature selection method adapted from a previous study on Parkinson’s disease (Vo et al., 2017). To find a set of components that best discriminated SCA3 from HC (i.e., the response variable), a matrix (33 subj × 23 ICs) containing all subjects’ IC variability loadings ($C_{i,j}$) (i.e., predictor data) was fed into lasso regression (*lassoglm* function of the Statistics and Machine Learning toolbox in MATLAB). This type of regression uses L1-regularization to reduce coefficient sizes for unimportant predictors (to a minimum of zero), thereby preventing overfitting, and adequately dealing with multicollinearity, which makes it highly suitable for selecting the most important features in a dataset. Lasso uses a range of regularization values, or lambda values, and computes the cross-validated error (deviance) for every lambda (using 10 fold cross-validation) (Hovens et al., 2019). The largest lambda value was used so that the deviance was within one standard error of the minimum deviance. To obtain a robust selection of ICs that best discriminated between patients and HC, we performed a bootstrap resampling procedure (5,000 iterations) with lasso regression performed during each bootstrap iteration. This resulted in a frequency histogram delineating how well ICs discriminate between the two groups. Subsequently, a selection of ICs was made based on the inflection point of the histogram, using the discrete first and second derivative (forward difference used for the first point, centered differences for the midpoints, and backward difference for the last point).

Because in the feature selection step all neural ICs were entered, coefficients were not specific for the final selected set of ICs. Therefore, an additional bootstrap resampling procedure (5,000 iterations) was conducted to compute a pattern image and associated individual subject scores by using only the selected ICs (as described in (Vo et al., 2017)). We now used logistic regression (*stepwiseglm* function in MATLAB) so that no further shrinkage was applied to the regression coefficients (which is the case for lasso regression). During every iteration, a linear combination of IC maps was made, based on the coefficients out of the fitted general linear model. Subsequently, a mean image was created across iterations, and coined the fSCA3-RP (Fig. 3). An additional image was created by masking out voxels for which the bootstrapped 99% confidence interval straddled zero, which were considered non-informative. Thus, the most stable voxels were visualized with this additional image. Subject scores were computed during every iteration by linearly combining the variability loadings $(C_{i,j}$) of the selected ICs based on the coefficients out of the general linear model. The final set of subject scores was established by calculating the mean score across iterations for every IC, and scaling these to the absolute sum of all ICs’ coefficients.

### 2.5 Structural analyses

To analyze brain atrophy, we performed ICA on grey matter segmentations using the so-called source based morphometry (SBM) toolbox, which is an extension of GIFT (Xu et al., 2009). A total of 10 ICs was extracted after MDL (FWHM) estimation, using Infomax and ICASSO (20 iterations) with selection of the best estimate for every IC (all ICs are shown in Suppl. Material 2). Mixing coefficients in the ICA mixing matrix $A_{\mathrm{ij}}$, where *i* represents subjects and *j* components, reflect how strong a subject contributes to a group grey matter component; these coefficients were used for subsequent analyses.

### 2.6 Statistical analyses and visualization

Continuous demographical and neuropsychological data were examined for group differences using independent t-tests, or Mann Whitney U tests (depending on the distribution) in SPSS version 27 (IBM Corp., Armonk, NY, USA). Shapiro Wilk tests were performed to assess continuous data for normality. Nominal variables were analyzed using Chi-square tests.

Associations of subjects’ fSCA3-RP scores with SARA, grey matter component subject scores, pSCA3-RP scores (i.e., the ^18^F-FDG PET pattern scores as published in (Meles et al., 2018)), disease duration, HADS scores, and neuropsychological test scores were investigated with Pearson or Spearman correlations using the *corr* and *partialcorr* functions in MATLAB. Shapiro-Wilk tests (*swtest* MATLAB function, provided by A. B. Saïda) were performed to test for normality. For neuropsychological test scores that were significantly correlated with SARA scores, we computed partial correlations (with SARA as covariate).

Group differences in grey matter component coefficients were calculated using independent t-tests, or Mann Whitney U tests (depending on the distribution) using MATLAB. Overall alpha was set at 0.05 with FDR-corrections in case of multiple comparisons (*mafdr* function in matlab).

Jittered data plots were made using notBoxPlot (version 1.31, by Rob Campbell) implemented in MATLAB. Histograms and scatter plots were created using custom made MATLAB scripts. Scatter plots in Supplementary Material 3 (Suppl. Fig. 5) were made using the fncCorrMatrixPlot function (version 2.0.0.01, by John Chow) implemented in MATLAB. Pattern images were overlaid on 2D brain slices using Chris Rorden’s MRIcroGL version 1.2.20210317.

## 3 Results

### 3.1 Demographics and clinical measures

Table 1 shows the demographics and clinical characteristics. Patients showed higher SARA scores, and impaired cognitive performance. Letter fluency scores were significantly negatively correlated with SARA scores in the group of patients with SCA3 (r = -0.6, P_FDR_ = 0.012). One patient had dystonic features, and two patients had parkinsonism.

**Table 1:** Participant characteristics.

	SCA3 (*n* = 17)	HC (*n* = 16)	*P-value*
Age, years	45.3 ± 11.4	49.4 ± 13.6	0.36
Sex, % female	50	46.1	0.87
Age at disease onset, years	35.6 ± 10.2	N/A	N/A
Disease duration, years	9.7 ± 7	N/A	N/A
CAG repeats^a^	70 ± 4	N/A	N/A
SARA^a^	10 ± 3.6	0 ± 0.9	<0.001
Education (range)^a,b^	5 (5–6)	5.5 (5–6)	0.31
Handedness n (%) right	14 (64.7)	11 (87.5)	0.13
HADS-A	3.5 ± 2.9	4.0 ± 2.4	0.63
HADS-D	5.3 ± 3.5	1.6 ± 1.5	0.001

*Neuropsychological tests:*			
Semantic fluency^a^	20.0 ± 6.5	26.2 ± 7.8	0.028
Letter fluency^a^	24.3 ± 12.0	38.6 ± 11.5	0.001
RAVLT total	42.6 ± 9.5	50.7 ± 9.0	0.021
RAVLT delayed	8.8 ± 2.8	11.4 ± 3.0	0.010
SDMT	44.4 ± 8.2	62.5 ± 9.6	<0.001

*Note*: All values are depicted as means ± SD unless stated otherwise. Statistics for neuropsychological tests were performed using raw scores.

^a^Median (interquartile range).

^b^According to (Verhage, 1964).

Abbreviations: CAG = cytosine-adenine-guanine trinucleotide; HADS-A = hospital anxiety and depression scale anxiety; HADS-D = hospital anxiety and depression scale depression; N/A = not applicable; SARA = Scale for Assessment and Rating of Ataxia; RAVLT = Rey Auditory Verbal Learning Test; SDMT = Symbol Digit Modalities Test.

### 3.2 fSCA3 related pattern identification

**Fig. 2:**
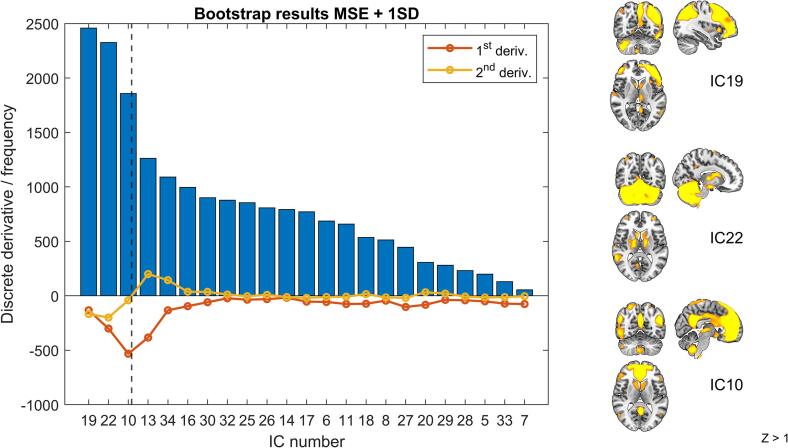
Bootstrapped feature selection results. Selection of IC 19, 22, and 10 was made based on the inflection point of the histogram (of non-artefactual ICs). It can be noticed that the first derivative (red) is minimal at IC10, and that the second derivative (yellow) crosses the x-axis (changes sign) between IC 10 and 13. Therefore, IC19, 22 and 10 were selected for further analyses.

The frequency results of the lasso regression analysis with bootstrap resampling procedure are depicted in Fig. 2. It is clear that the first derivative has a minimum at IC10, and the second derivative crosses the x-axis between IC 10 and 13. Therefore, IC19, 22 and 10 were selected as predictive components, corresponding with a left frontoparietal, cerebellar, and anterior default network component, respectively.

**Fig. 3:**
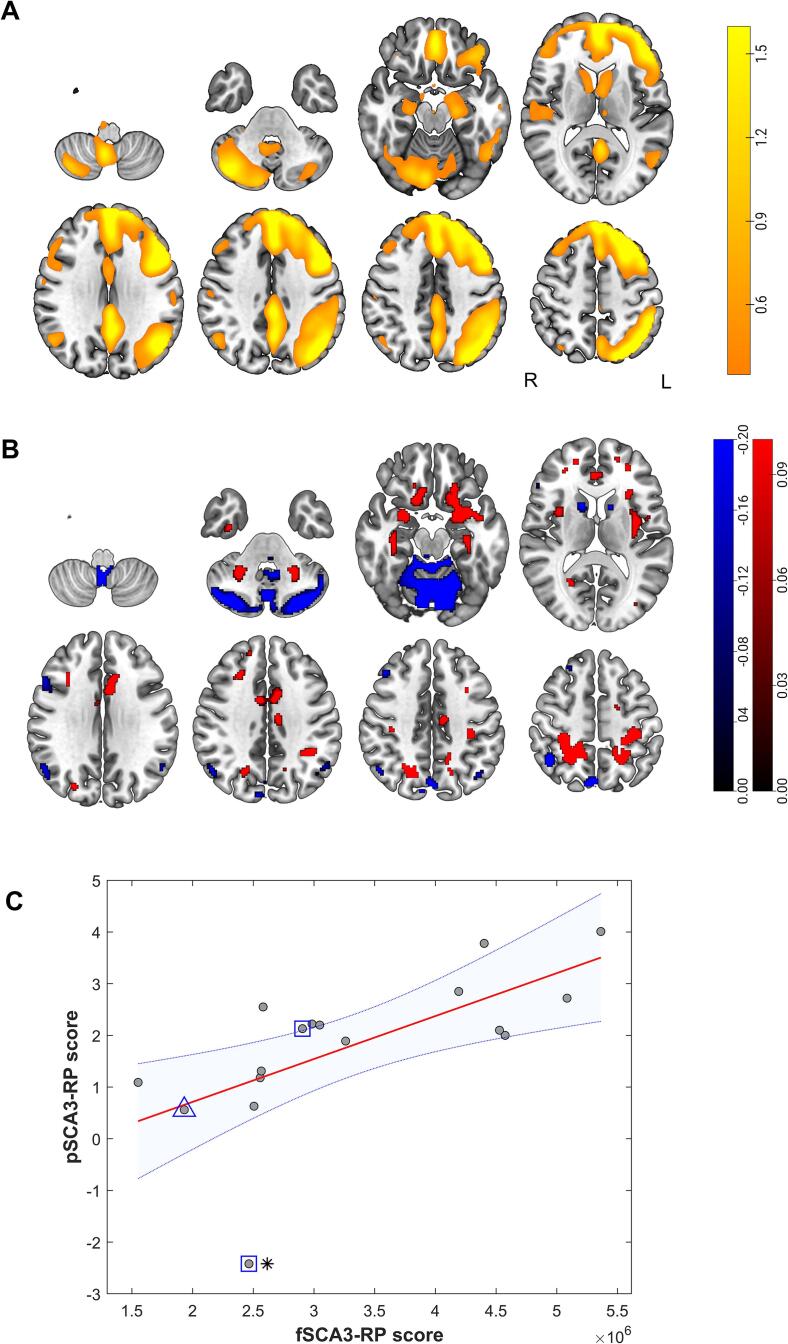
A: The fSCA3-RP (fMRI). Positive voxels (thresholded at Z > 0.5) are shown, representing a magnitude of changed functional connectivity in the marked regions. Color scale reflects the linearly combined Z-values of the mean group independent components. B: The pSCA3-RP (^18^F-FDG PET; red voxels = relative hypermetabolism, blue = relative hypometabolism), as derived from the same dataset (Meles et al., 2018). Color scale reflects median of voxel values obtained with bootstrap resampling. C: Correlation between fSCA3-RP and pSCA3-RP scores (rho = 0.78, P = 0.0003). A subject’s fSCA3-RP score represents the linear combination (based on regression coefficients) of L2-distances for the selected components (IC19, 22 and 10) of the subject. The outlier (indicated by an asterisk) on pSCA3-RP used levodopa at time of scanning. Patients with parkinsonism are indicated by a square, and the patient with dystonic features is indicated by a triangle.

The linear combination of these components using logistic regression coefficients (0.8740, 0.2831, and 0.6826 for IC19, 22 and 10, respectively; for the histograms of coefficients see Supplementary Materials 3 (Suppl. Fig. 1). resulted in the fSCA3-RP (Fig. 3A). The fSCA3-RP involved the cerebellar hemispheres and vermis, the medial temporal lobe (parahippocampal gyrus), the orbitofrontal cortex, the medial frontal cortex, the antero-lateral frontal cortex of particularly the left hemisphere, the cingulate, and the caudate nuclei. A figure showing the most stable voxels outside the 99% confidence interval can be found in Supplementary Materials 3 (Suppl. Fig. 2). When the (full) mean positive pattern was shown, a more global left cerebral hemisphere lateralization was seen, contralateral to the dominant right-sided cerebellum involvement. Both statistical measures demonstrated this stronger right than left cerebellar involvement.

Fig. 3C shows that scores on the fSCA3-RP were highly correlated (rho = 0.78, P = 0.0003) with scores on a ^18^F-FDG-PET pattern (Fig. 3B) that was published previously.

A jittered data plot (including mean, 95% confidence interval, and one standard deviation) showing the subject scores per group can be found in Supplementary Materials 3 (Suppl. Fig. 3).

### 3.3 Associations with clinical measures

**Fig. 4:**
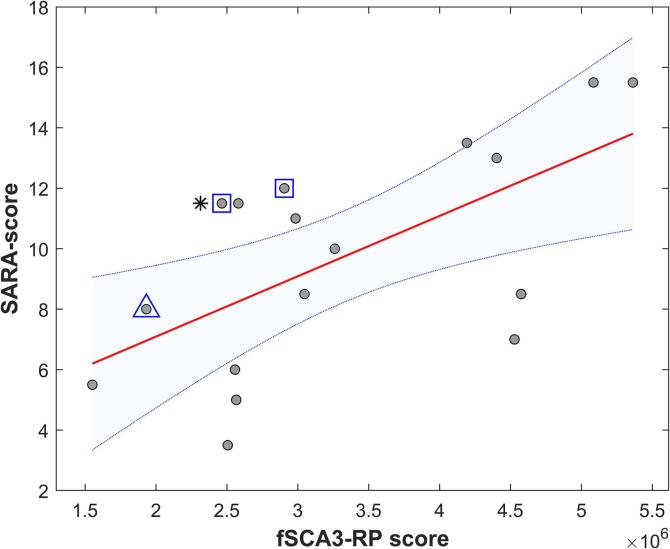
Correlation between fSCA3-RP scores and SARA scores for the SCA3-group. The patient who used levodopa at time of scanning is indicated by an asterisk; patients with parkinsonism are indicated by a square; the patient with dystonic features is indicated by a triangle.

The subject specific fSCA3-RP scores were significantly correlated with SARA scores within the SCA3 group (r = 0.63, P = 0.007; Fig. 4).

No significant correlations were found between fSCA3-RP scores and neurocognitive measures in the SCA3 group. However, when investigating the three components in the fSCA3-RP separately, we found a significant negative correlation between SDMT scores (mental processing speed, with higher scores indicating a higher speed) and L2 distances for a cerebellothalamic (IC22) component in the SCA3 group (-0.65, P = 0.0045).

There were no significant correlations between fSCA3-RP scores and HADS-scores, or disease duration.

### 3.4 Associations between fSCA3-RP scores and brain atrophy

**Fig. 5:**
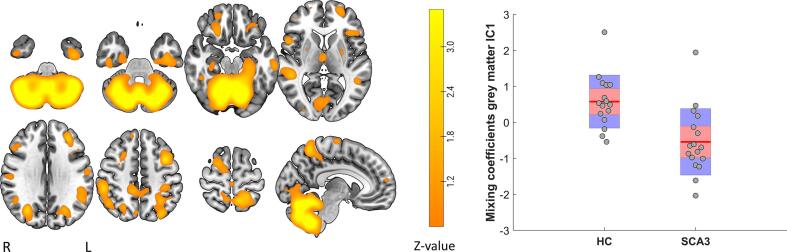
Grey matter IC1. To enhance interpretability only voxels with positive z-scores are shown. A clear group difference was found, with SCA3 patients have lower mixing coefficients (i.e., their spatial maps contribute less to the average group map), indicating grey matter loss.

For a cerebellar grey matter component (GM-IC1), significantly lower mixing coefficients were found for SCA3 patients relative to HC (P_FDR_ = 0.006; Fig. 5). However, no significant correlations were found of these mixing coefficients with fSCA3-RP scores, nor with subject scores on the individual fMRI ICs. Also, there was no significant correlation between GM-IC1 mixing coefficients and SARA scores. For several grey matter components, however, significant or trend significant group differences were found at an uncorrected alpha of 0.05 (Fig. 4 in Suppl. Materials 3). For these components, moderate to strong correlations were found with pSCA3-RP, fSCA3-RP and/or SARA scores (Fig. 5 in Supplementary Materials 3).

## 4 Discussion

This study reports resting-state brain network changes using fMRI in patients with SCA3. We identified a resting-state fMRI pattern (the fSCA3-RP) that was characterized by functional connectivity changes within cerebellar-cerebral and striatal-cortical networks. Individual fSCA3-RP expression scores correlated significantly with the SARA scores, a clinical scale that reflects the severity of ataxia. Further support for the biological relevance of the fSCA3-RP is provided by the strong correlation between subject scores on the fSCA3-RP and its previously discovered metabolic counterpart, the pSCA3-RP (Meles et al., 2018).

The fSCA3-RP is characterized by altered (relative) functional connectivity in the cerebellum, parahippocampal areas, the medial prefrontal / cingulate cortex, caudate nuclei, thalamus and lateral frontoparietal cortical regions. Previous task-based activation fMRI and resting-state ^18^F-FDG-PET studies have also found activity changes in the cerebellum and basal ganglia, and also in frontoparietal cortical areas (Duarte et al., 2016, Meles et al., 2018, Stefanescu et al., 2015, Wang et al., 2007). This is likely a reflection of altered connectivity in cerebellar-cerebral and striatal-cortical networks due to SCA3-associated pathology in cerebellum, striatum, brainstem and pyramidal tracts (Rüb et al., 2008). Indeed, the fSCA3-RP topography, combined with the significant correlation with ataxia severity, suggests that the fSCA3-RP reflects network-level changes in (higher-order) motor control. The significance of the obvious overlap between the regions identified in the fMRI and ^18^F-FDG PET-derived SCA3 patterns was further supported by the strong correlation between subject scores obtained from the two data-sets concerning the same patients. Both patterns include the cerebellum, medial temporal lobe, the caudate nuclei, the cingulate and the posterior parietal cortex.

However, the two patterns do not correspond exactly. For instance, the fMRI pattern shows more extensive alterations in the prefrontal cortex, which is particularly strong in the medial frontal cortex, including the cingulate cortex. Due to the differences between the two modalities, as well as analysis techniques (PCA vs. ICA), a direct comparison of the two patterns in terms of involved regions cannot be provided. That said, it is of interest to speculate about the similarities and discrepancies in the identified networks in the context of the basic physiological and biophysical differences between the ^18^F-FDG PET and BOLD techniques. Especially large-scale anterior-posterior networks, such as the default mode network and frontoparietal executive networks have been described to be more strongly connected in BOLD than in ^18^F-FDG PET images (Di & Biswal, 2012). This was explained by the difference between a static (cumulative) metabolic activity of regions, and the summation of BOLD fluctuations over the time of scanning. Here the higher temporal resolution of rs-fMRI might also explain the identification of changes in inter-regional connectivity which were not observed in the static ^18^F-FDG PET images. The extensive mediofrontal involvement in specifically the fSCA3-RP may thus well represent changes in default network activity, which is fluctuating in nature. This will be further treated in following paragraph. The coherent dominance of change in the right cerebellum and left cerebral cortex, which was only observed in the fSCA3-RP, and not in the pSCA3-RP, similarly suggest a change in the dynamic nature of a specific left-hemisphere function. Although intriguing, we refrain from further speculation on this issue.

While the fSCA3-RP topography showed a significant correlation with ataxia severity, we did not find any significant correlations between fSCA3-RP scores and any of the neurocognitive measures we investigated. SCA3 patients, however, do experience cognitive dysfunction in addition to their motor symptoms. We did find a negative correlation between one of the three components in the fSCA3-RP (a cerebello-limbic-thalamo-cortical, but mainly cerebellar component) and mental processing speed. This finding fits with the cognitive (and affective) functions of the cerebellum, through connections with cortical and limbic structures (Schmahmann & Sherman, 1998). A recent systematic review concluded that the neurocognitive profile of SCA3 is mainly characterized by impairments of executive function (Yap et al., 2021). Prefrontal-striatal circuitry is implied in such higher-order cognitive functions. On the other hand, we propose that the extensive medial prefrontal presence in our fSCA3-RP is not a reflection of change in cognitive function of the patients but may represent default mode network changes, because this medial prefrontal involvement was evidently less extensively present in the ^18^F-FDG pattern, and the fSCA3-RP did not correlate with cognitive impairment. The difference with the ^18^F-FDG pattern is consistent with the fact that particularly the BOLD fMRI method enables detection of dynamic changes concerning fluctuation of neuronal activity, which is an intrinsic characteristic of the default network. We do acknowledge that the absence of a correlation with cognitive impairment might also be due to the fact that the pattern was sought amongst those components that gave maximum discrimination between controls and patients. All patients have ataxia, but the presence of cognitive symptoms was variable, and relationships with network connectivity might have been hidden in the larger fSCA3-RP. But even in that case, based on a strong discriminator between controls and patients, the proposed relation of mediofrontal change with changed fluctuations in default mode activity gains support. Finally, when combining fMRI and ^18^F-FDG PET studies, care has to be taken regarding the spatial mismatch between the two modalities (Jamadar et al., 2019).

Both the fMRI and the ^18^F-FDG PET pattern thus provide a modality-specific signature of SCA3 and they are highly correlated in the same individuals, with a biological significance of network changes and their metabolic alterations. Although they have complementary components, our results suggest that the fSCA3-RP and the pSCA3-RP can be used interchangeably to quantify disease-related changes on the single-subject level, akin to the conclusions of a previous similar study in Parkinson’s disease (Vo et al., 2017). This means that several promising results obtained with ^18^F-FDG PET and PCA in neurodegenerative diseases (Meles et al., 2021, Schindlbeck and Eidelberg, 2018) may also be reached using fMRI-ICA based networks. This application of fMRI is thus potentially useful as robust neuroimaging biomarkers are necessary for these conditions of neurodegenerative disease (regarding diagnosis, monitoring disease progression and treatment effects), and fMRI is considered less invasive and time-consuming than ^18^F-FDG PET.

Aside from its use in diagnostic processes, one might reflect on possible therapeutic implications of ‘network knowledge’ involving cerebellum, caudate and various cortical cerebral regions. The association of specific brain circuitry and the representation of distinct cerebral functions may, in this respect, help to choose targeted tests in motor, cognitive and emotion domains. The results of such tests help to design strategies to effectively cope with emerging dysfunction. Regarding therapy, one might theoretically speculate that affected network function might be brought in a better functional balance by e.g., deep brain stimulation of distinct network nodes. For such a putative strategy, much more knowledge about the networks involved and the dynamics of change over time is required. Similarly, manipulation of distinct network nodes might be guided by knowledge about specific neurotransmitter modulation of an involved brain region (e.g., dopaminergic or serotonergic).

While functional brain imaging may identify disease-related changes ahead of structural imaging, the identification of regional atrophy evidently marks the loss of tissue. In SCA3, disease progression has been demonstrated to occur with a spread of successively affected brain regions (Guo et al., 2020). Using T1 MRI and structural covariance analysis, Guo and colleagues demonstrated a pattern of progressive grey-matter atrophy in a cohort of 47 SCA3 patients with similar CAG repeat lengths, by dividing them into subgroups based on disease stage (pre-manifest, early (1–5 years), intermediate (6–10 years) and final (11–20 years after onset). Atrophy started in the cerebellar vermis in pre-manifest carriers, and extended into the cerebellar hemispheres in the early stage. In the intermediate stage, degeneration also affected the striatum, and in the final stage, atrophy of the motor and association cortex, including the paracentral gyrus, the pre-central gyrus, and the supplementary motor area was identified. The patients in our cohort had a variable disease duration (range 3–30; median 7 years). The regions identified in the fSCA3-RP and pSCA3-RP partly overlap with the atrophy patterns of the final stage described by Guo et al (11–20 years; cerebellum, striatum, motor and association cortex) (Guo et al., 2020). However, the primary motor cortex itself is not implicated in our functional imaging patterns. Furthermore, some regions show alterations on a functional level (lateral frontal and posterior parietal association areas, parahippocampal gyrus, cingulate cortex), whereas they remain unaffected structurally throughout the disease course. This may provide support for the concept that these regions show altered neuronal activity due to disconnection (or other downstream network effects) from cerebellum and striatum, two core structures that are directly affected by local pathology. Regarding structural disconnection, studies have also found evidence for affected (infratentorial) white matter structures, and an association with disease progression and ataxia severity (Faber et al., 2021, Rezende et al., 2018).

Interestingly, neither fSCA3-RP, nor pSCA-RP subject scores were significantly correlated with grey matter atrophy. fSCA3-RP scores specifically, did not correlate with mixing coefficients of a grey matter component that was significantly different between groups. The variation in disease duration (3 – 30 years), clearly associated with variation in distributed atrophy, may play a role in this seeming discrepancy. While the end-stage pattern of gray matter atrophy may show the best correlation with the functional imaging patterns (see also above listed regional distribution of Guo et al), the fSCA3-RP and pSCA3-RP represent extended network dysfunction that emerge at earlier stages of disease, exceeding nodes in the network that are affected by atrophy. Nonetheless, several grey matter components, that were not (yet) significantly different between SCA3 and controls, correlated with scores on the pSCA3-RP, fSCA3-RP and/or SARA scores (Suppl. Materials 3). Therefore, we have reason to believe that these components reflect early changes in grey matter degradation that occur alongside changes in metabolic and dynamic BOLD fluctuations. In this respect, our results suggest that the changes detected by our functional neuroimaging modalities indeed precede atrophy and provide a better understanding of the widespread network-level alterations in SCA3.

Although limitations of this study are the small sample size and the lack of a validation sample, we are confident that the fSCA3-RP represents distinct pathophysiological changes and did not arise by chance, since fSCA3-RP subject scores are clearly correlated with a clinical scale of disease severity. Furthermore, it is striking that these subject scores also correlated strongly with pattern scores derived from a different modality (^18^F-FDG PET). The latter can be considered as an indirect measure of internal validity. We also used bootstrap resampling to obtain a more stable selection of components as compared to only using the sample itself. Our group component maps (4D NIfTI file) are available upon request (to be used for reference-based ICA and generation of subject scores using our regression coefficients), and we kindly invite other researchers on SCA3 to corroborate our findings. Lastly, in the current study we only focused on cognitive functioning as a non-ataxia sign. Future studies may benefit from the use of other tools, such as the Inventory of Non-Ataxia Signs, to further delineate disease-related patterns in SCA3 and the relationship with different phenotypes (Jacobi et al., 2012).

## 5 Conclusion

In contrast to most ICA-based fMRI studies, we provide a quantification of disease activity in resting-state fMRI scans of individual subjects with SCA3. Such quantification may provide a biomarker for SCA3 and could be useful in predicting disease onset in pre-manifest carriers, tracking disease progression, or perhaps even as an outcome measure in future clinical trials. To that end, it is crucial to investigate the genetic – phenotypical relation over time, and to determine the specificity of the fSCA3-RP for SCA3, and possible overlap with other ataxias. The present findings encourage future research directed towards these goals.

## Declaration of Competing Interest

The authors declare that they have no known competing financial interests or personal relationships that could have appeared to influence the work reported in this paper.
