# Supplementary figures and images for: A resting-state fMRI pattern of spinocerebellar ataxia type 3 and comparison with 18F-FDG PET
Source: Neuroimage Clin. 2022 Apr 25;34:103023. doi: 10.1016/j.nicl.2022.103023 (PMC9062756; doi:10.1016/j.nicl.2022.103023)

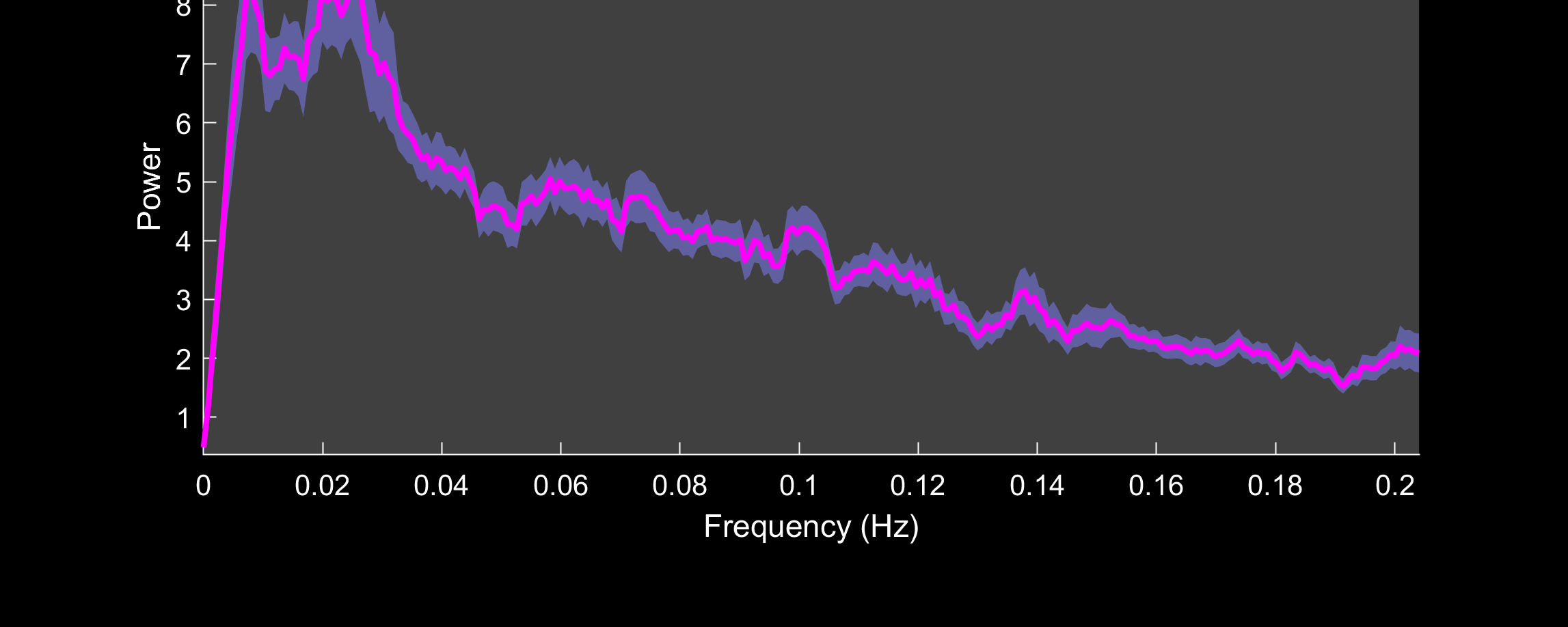

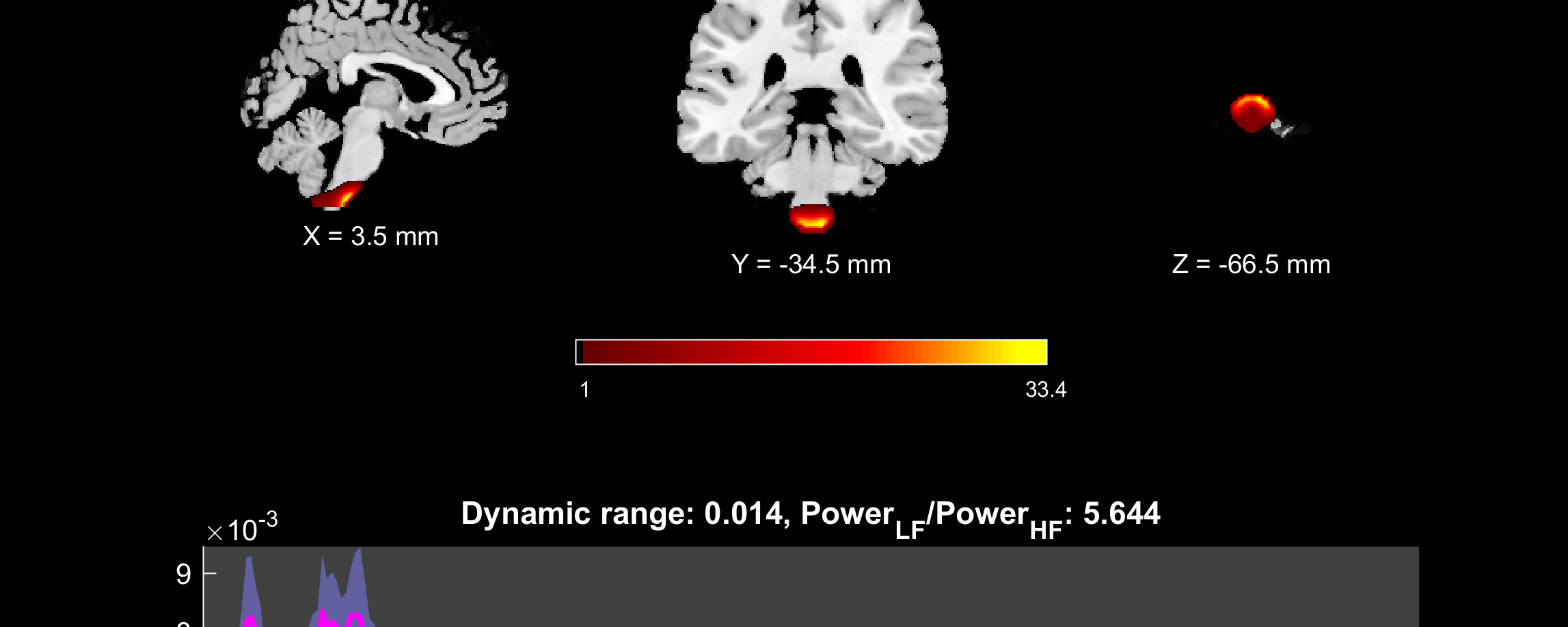

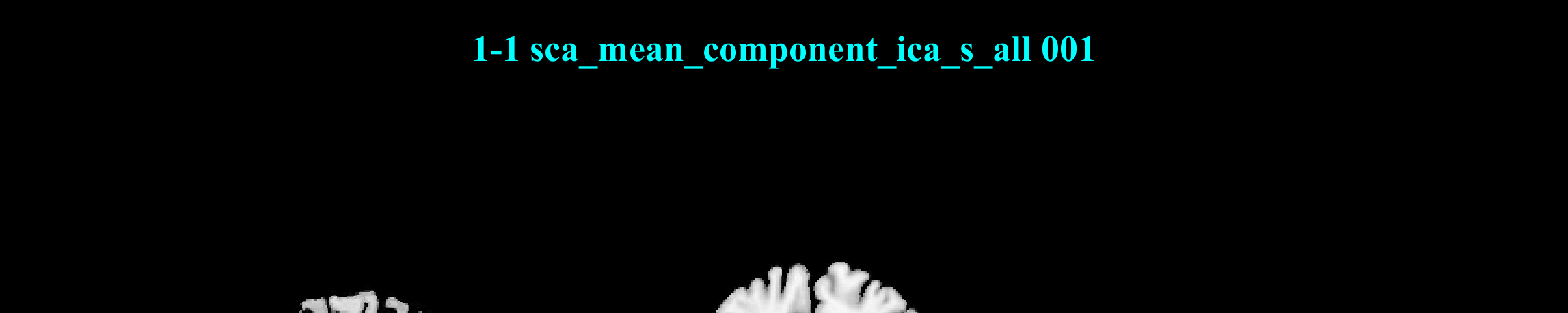

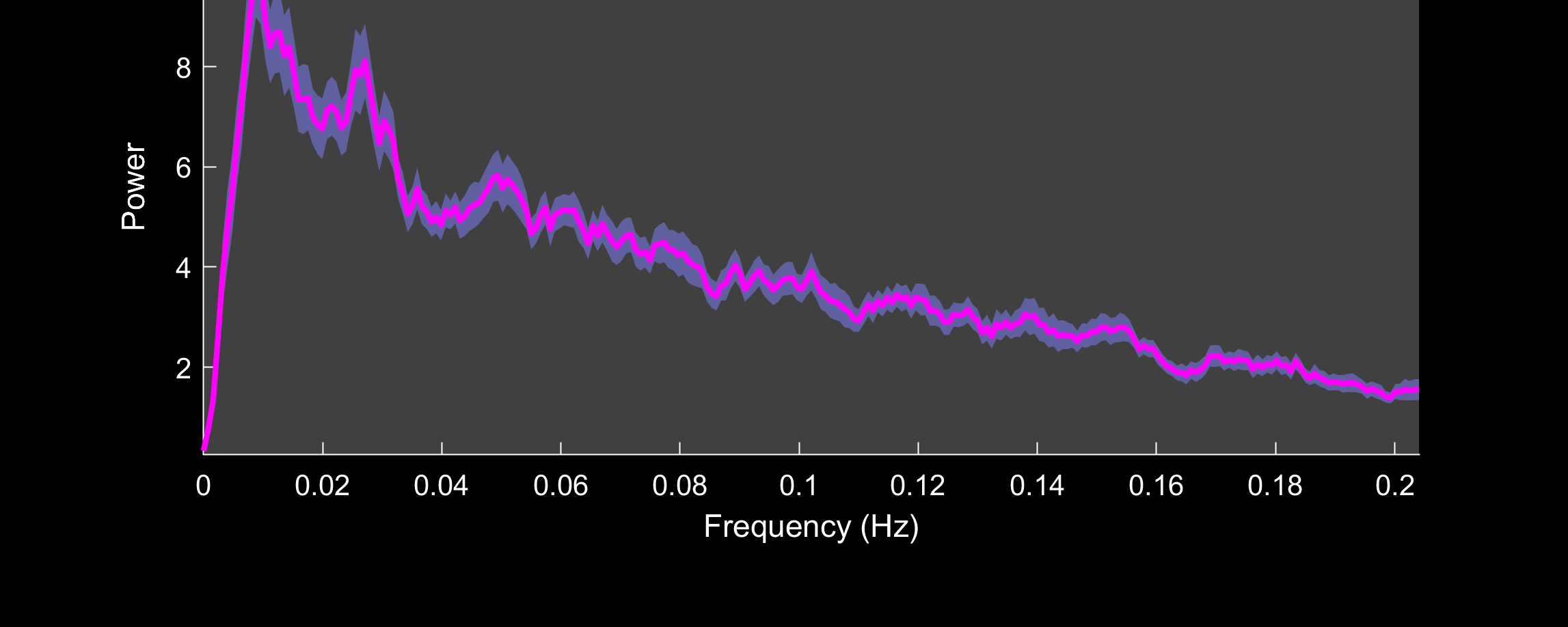

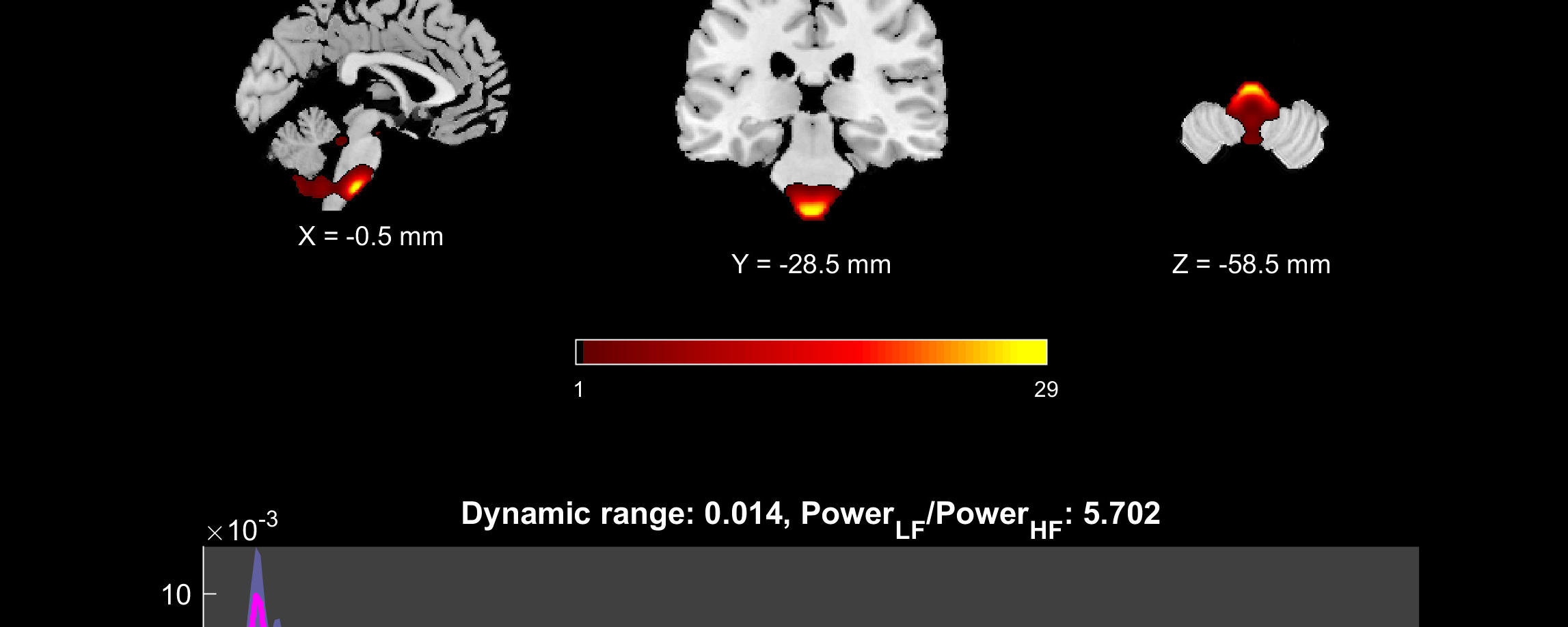

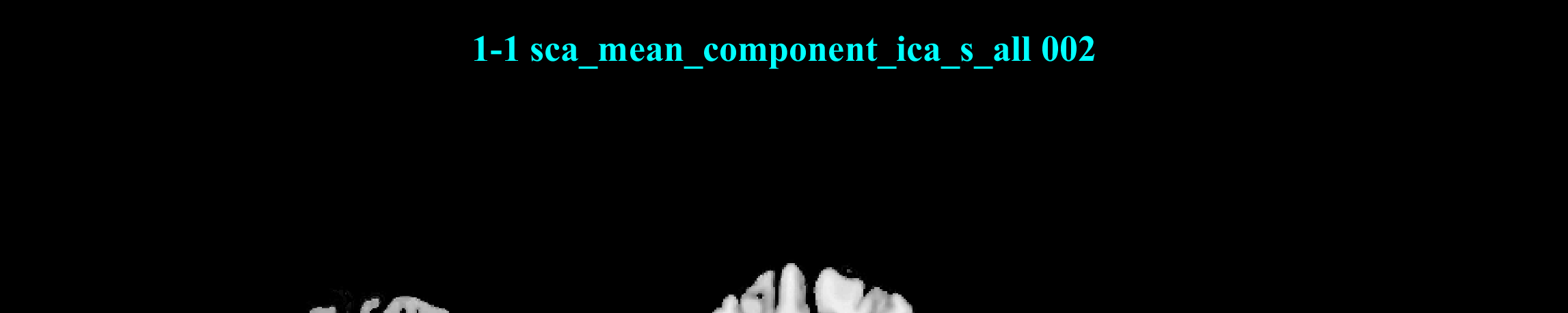

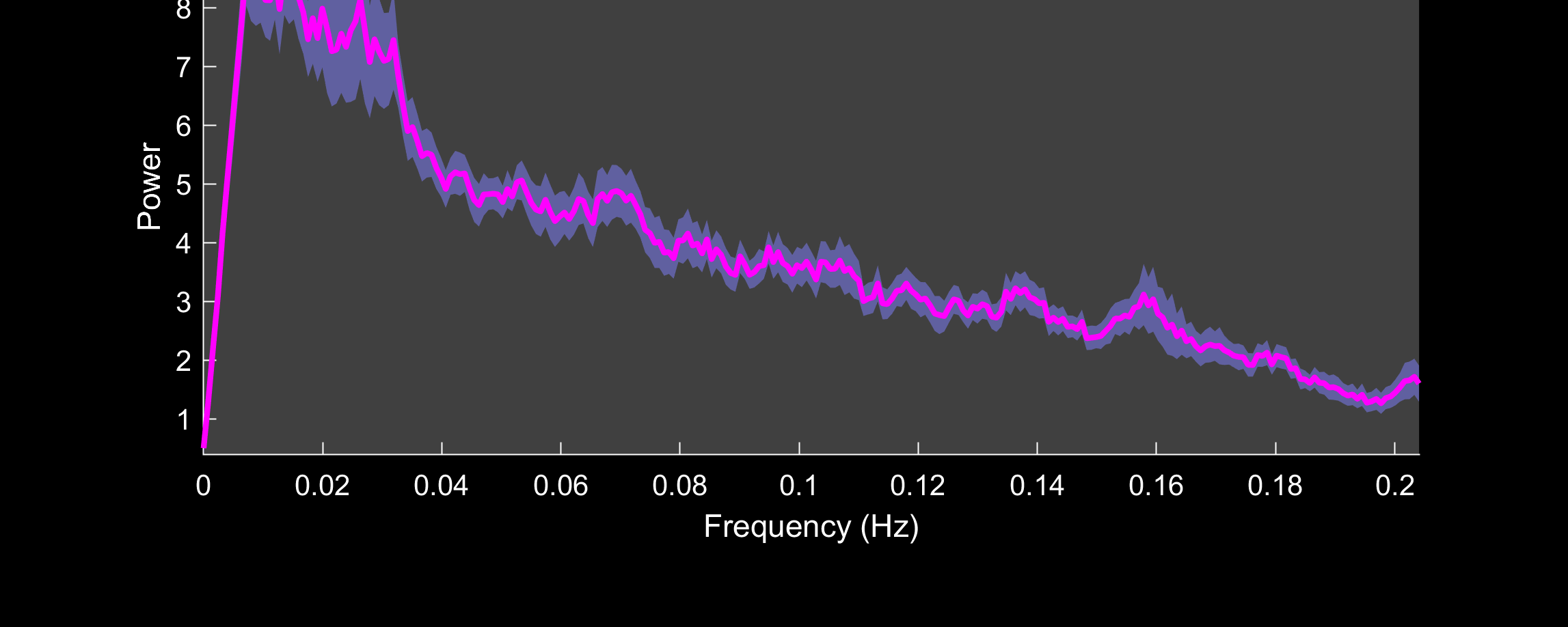

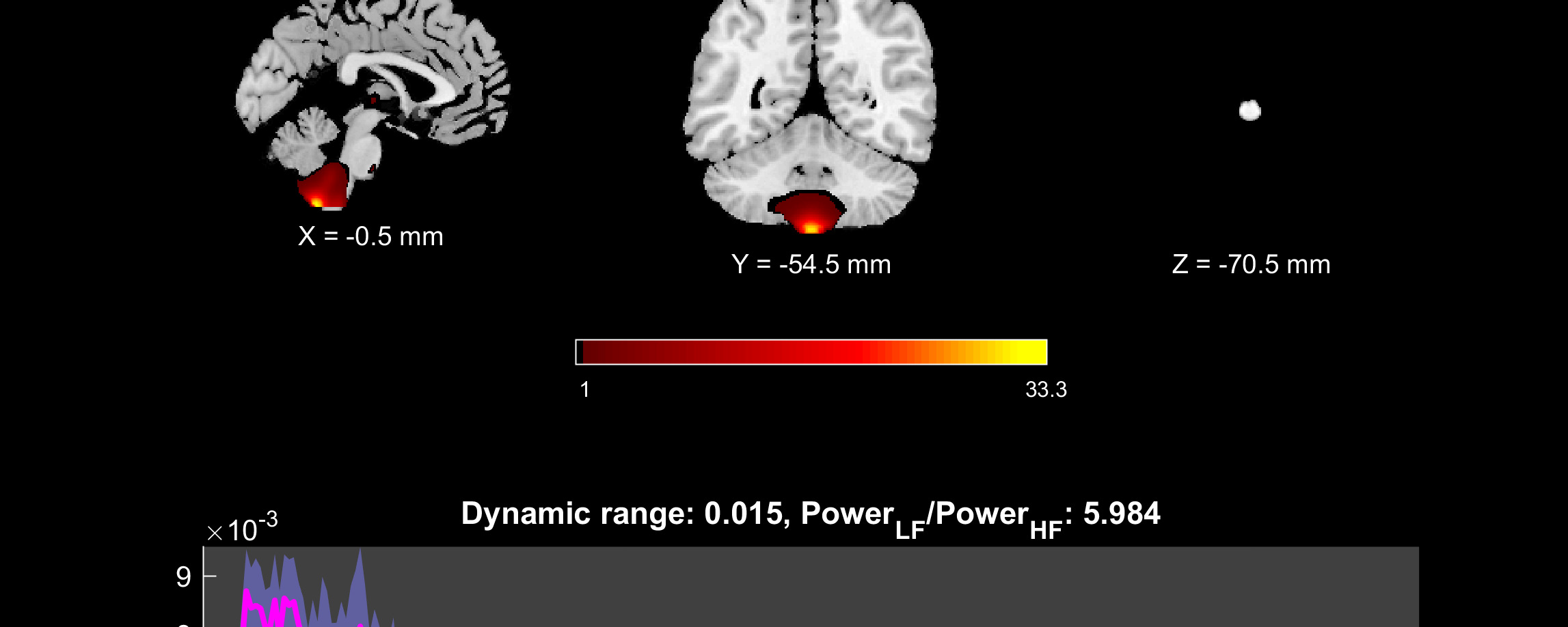

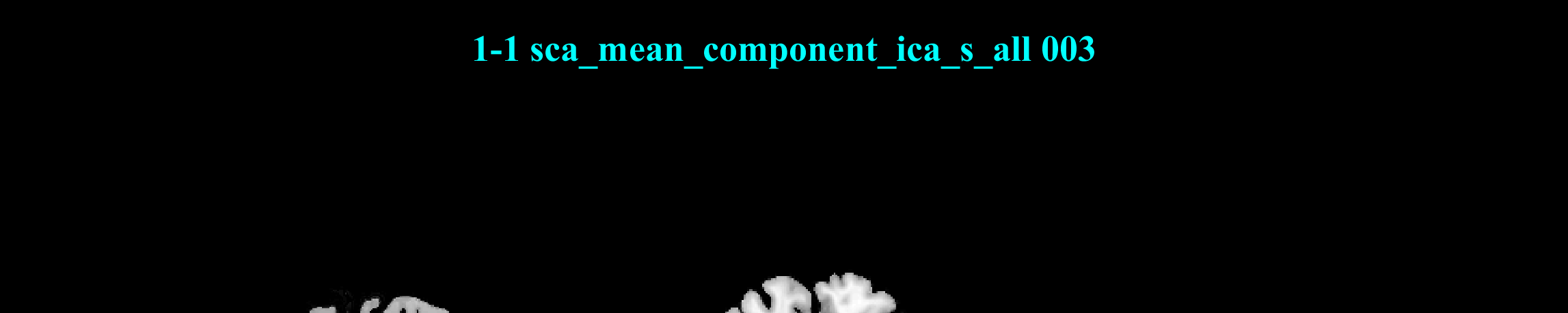

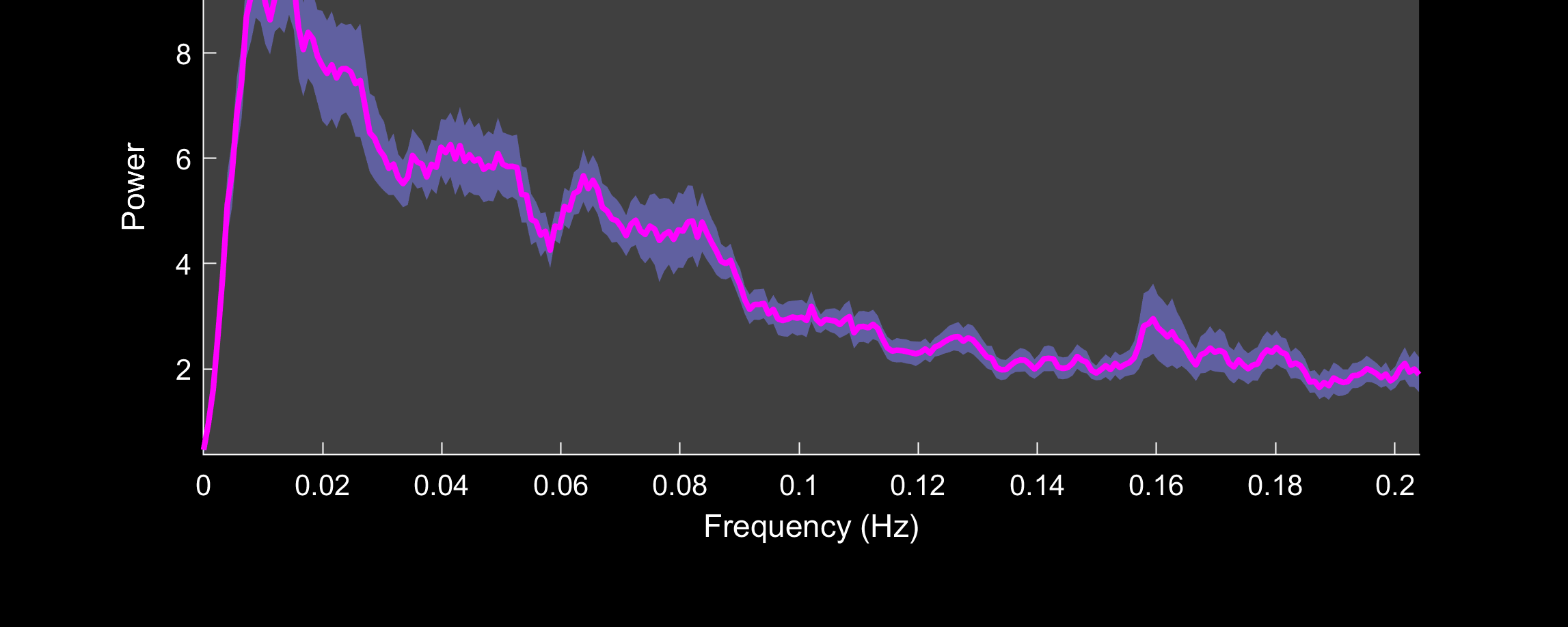

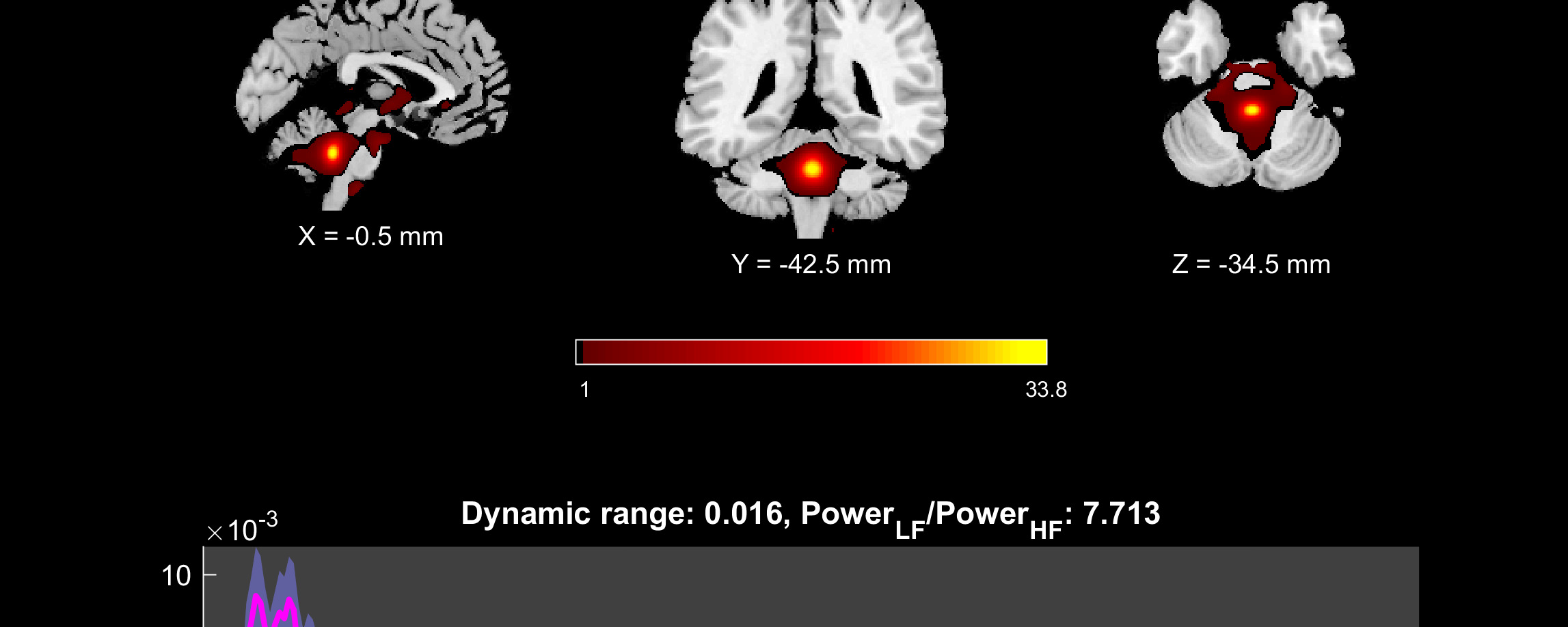

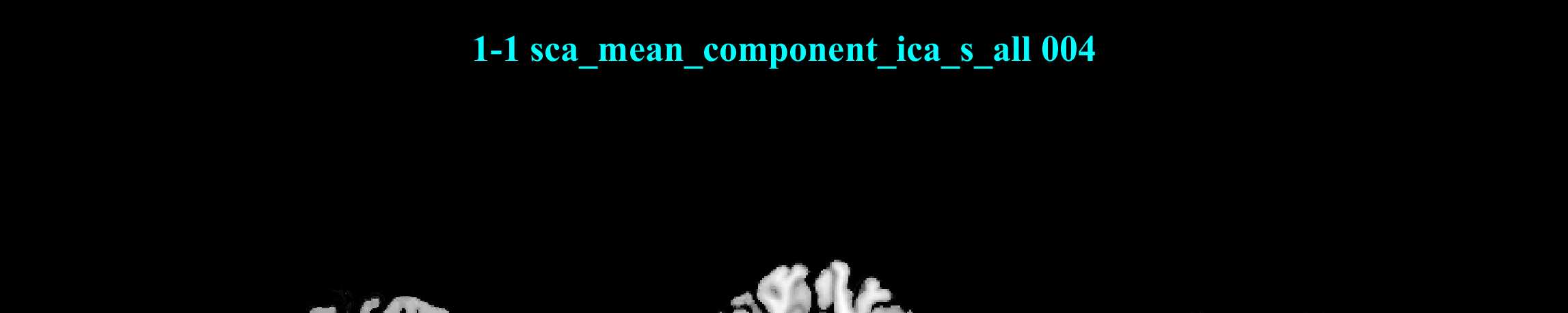

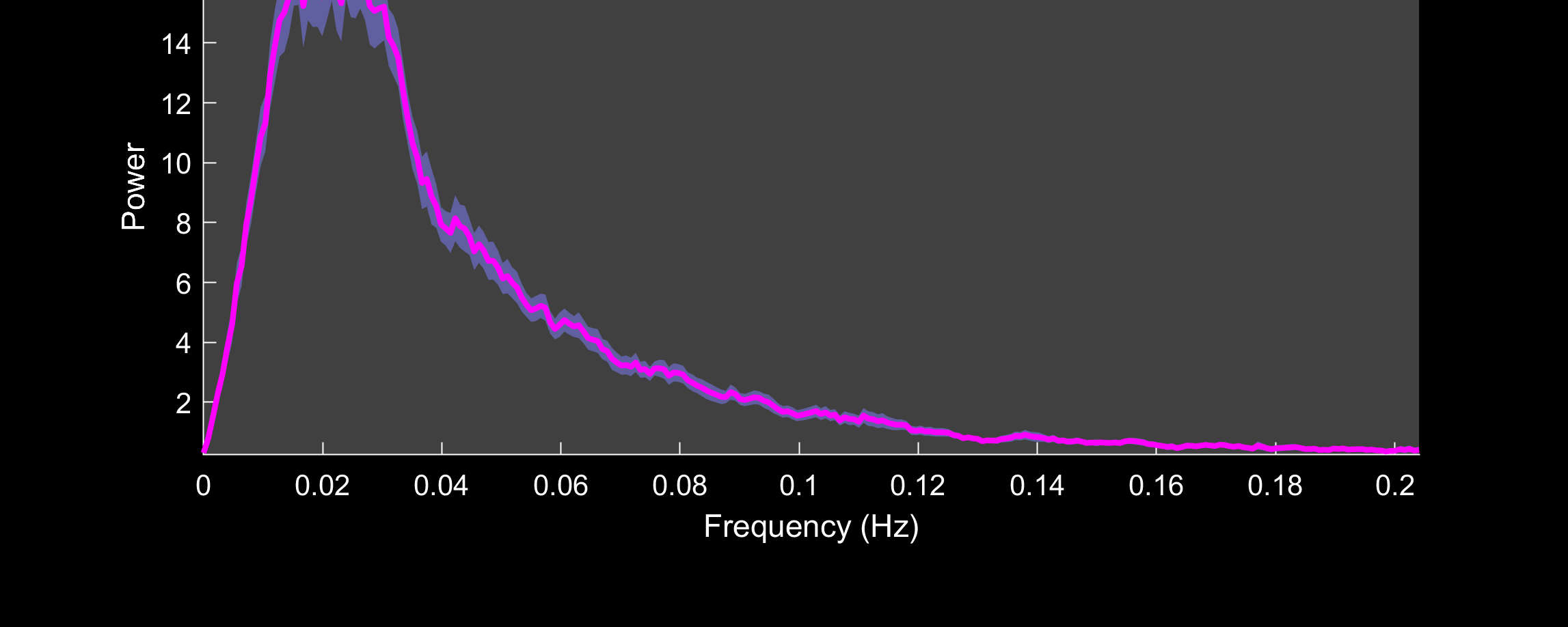

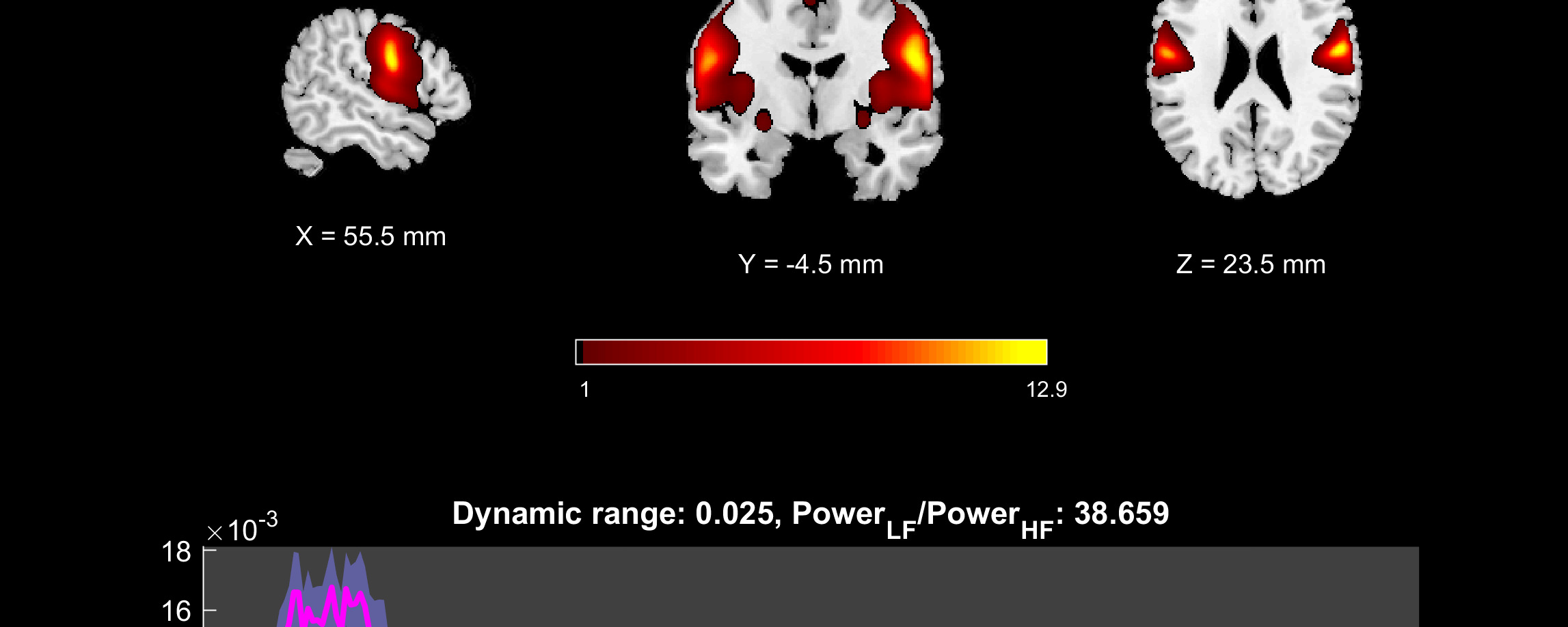

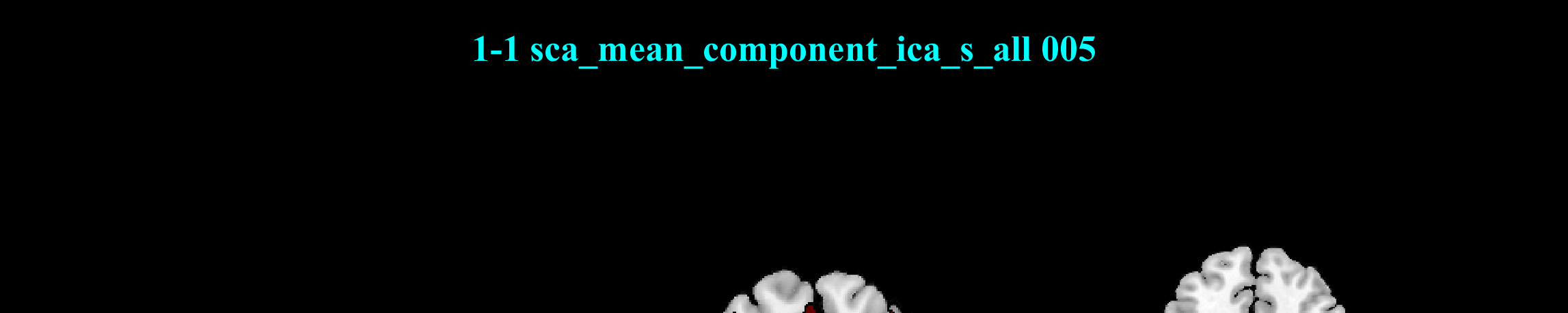

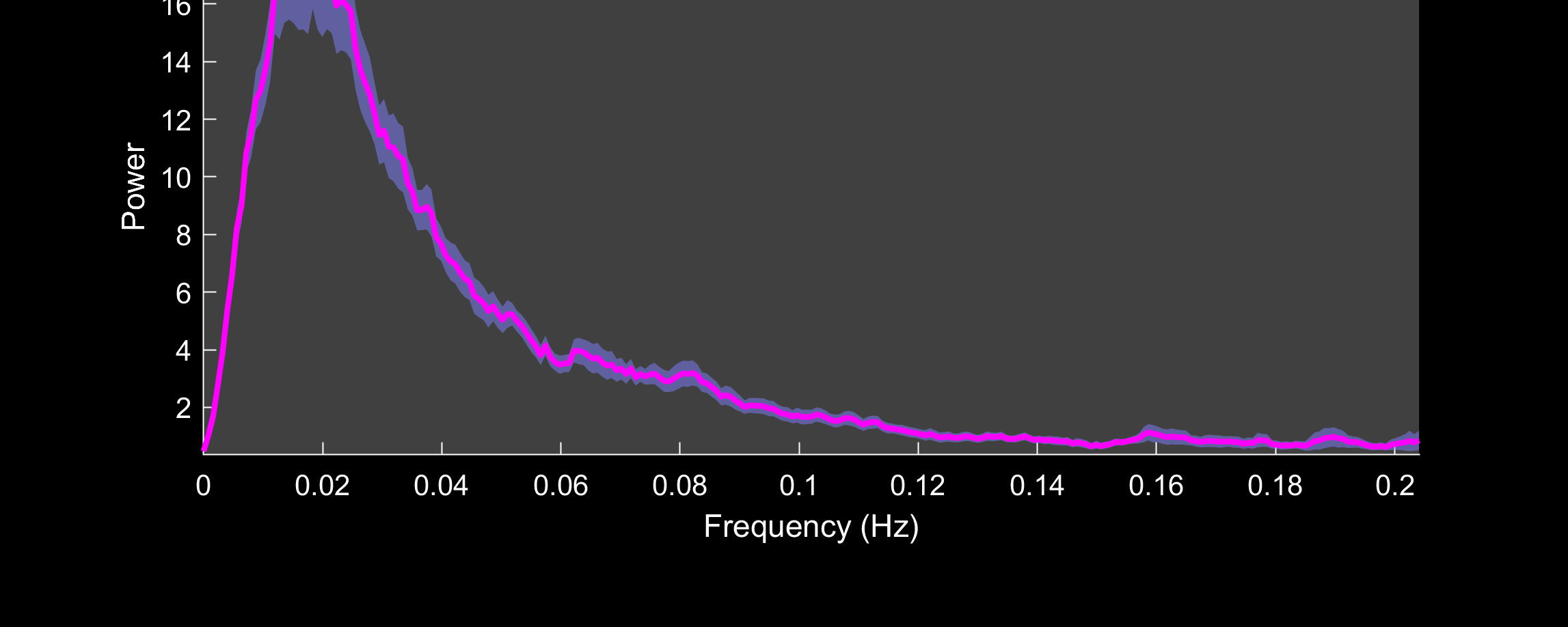

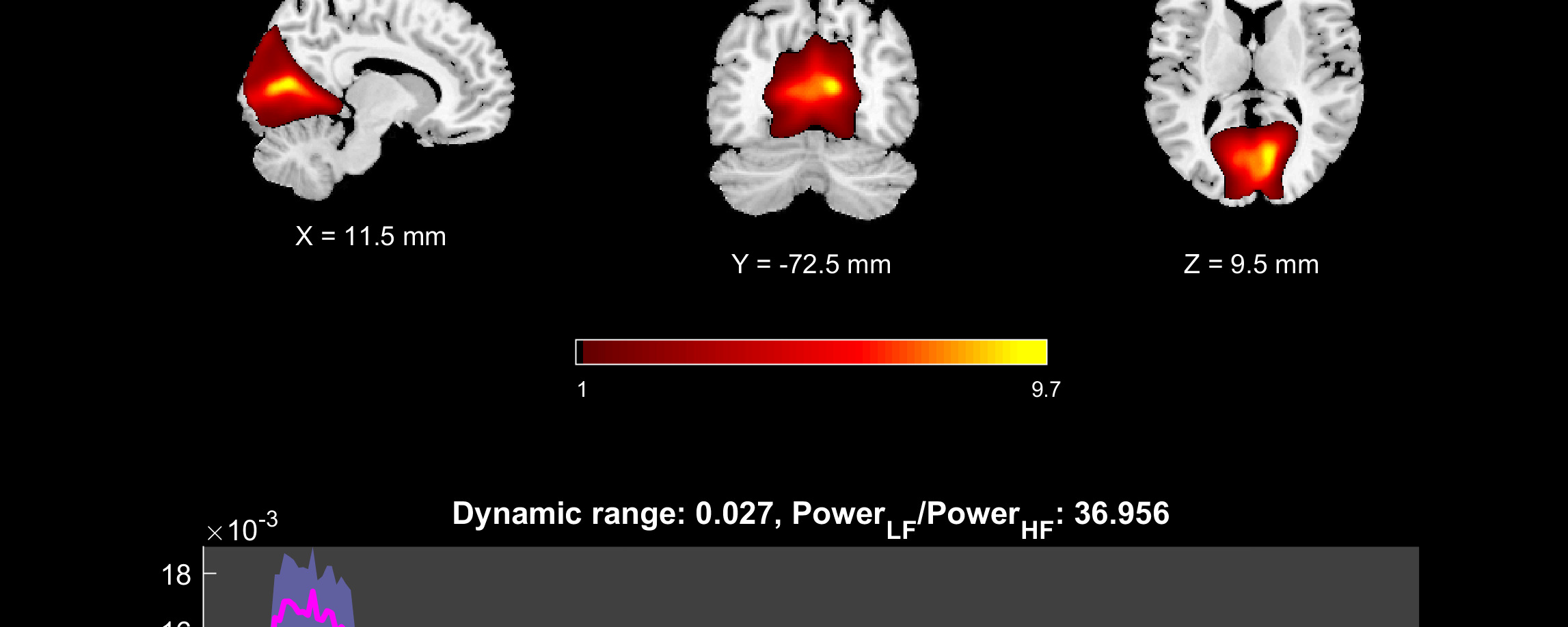

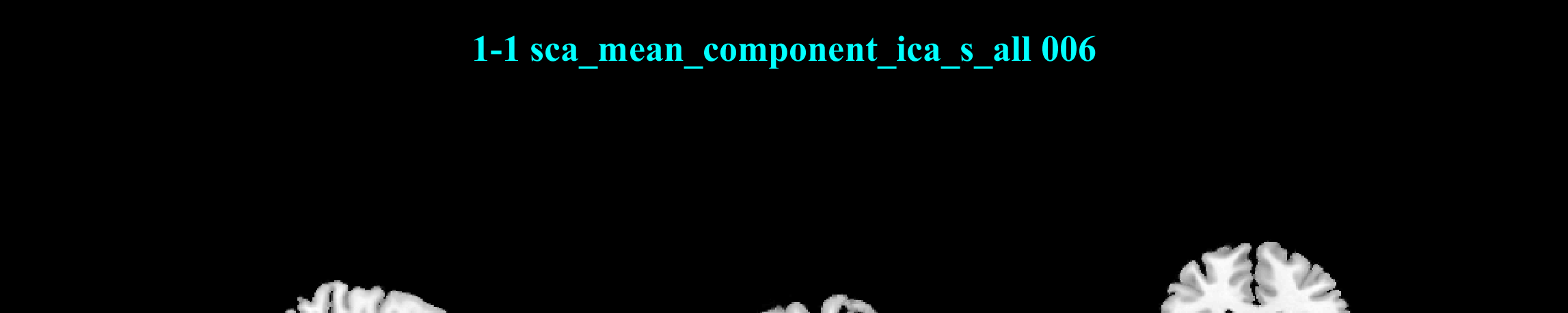

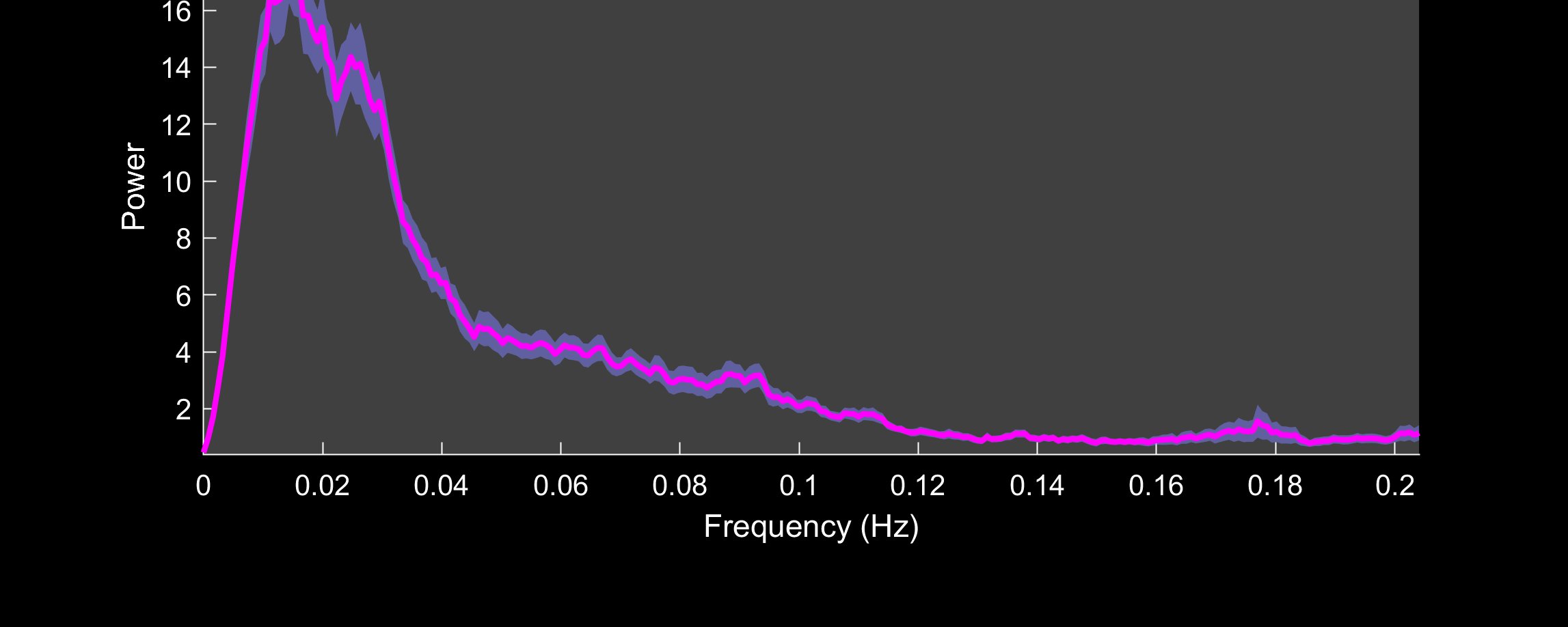

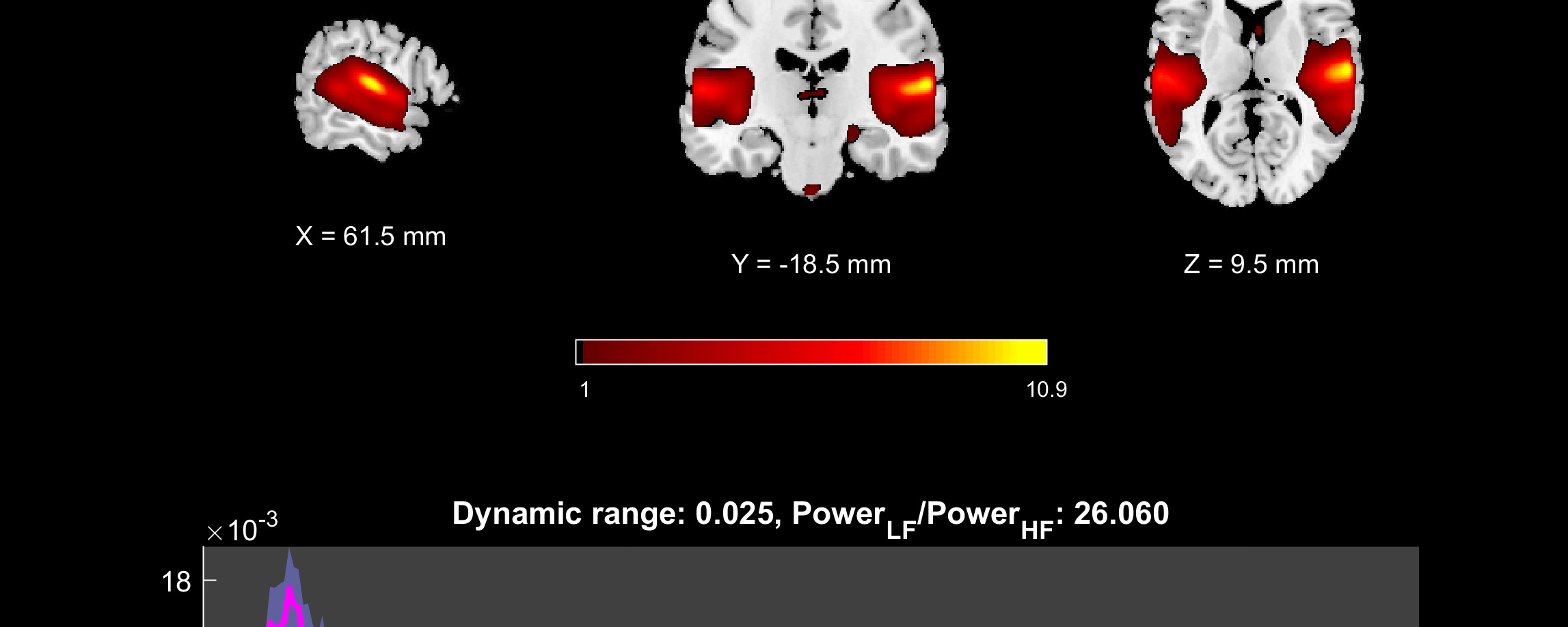

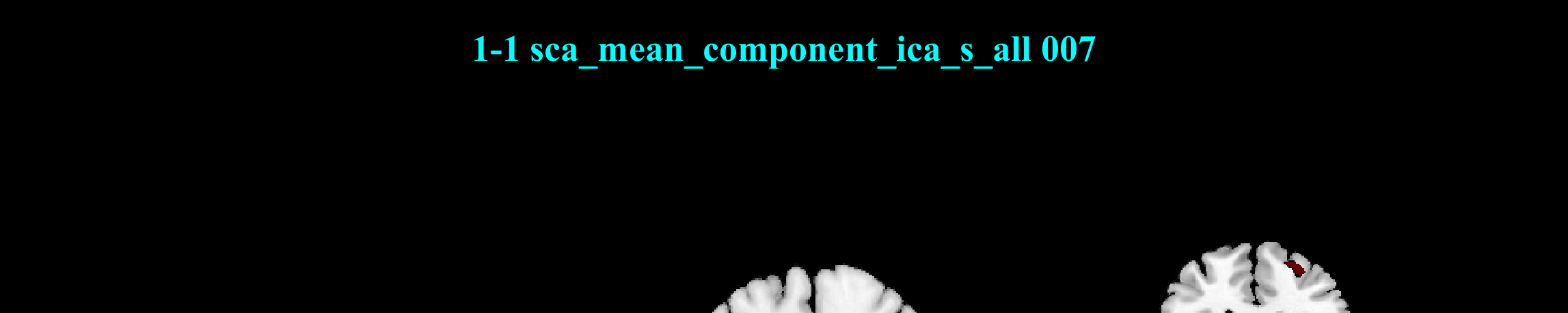

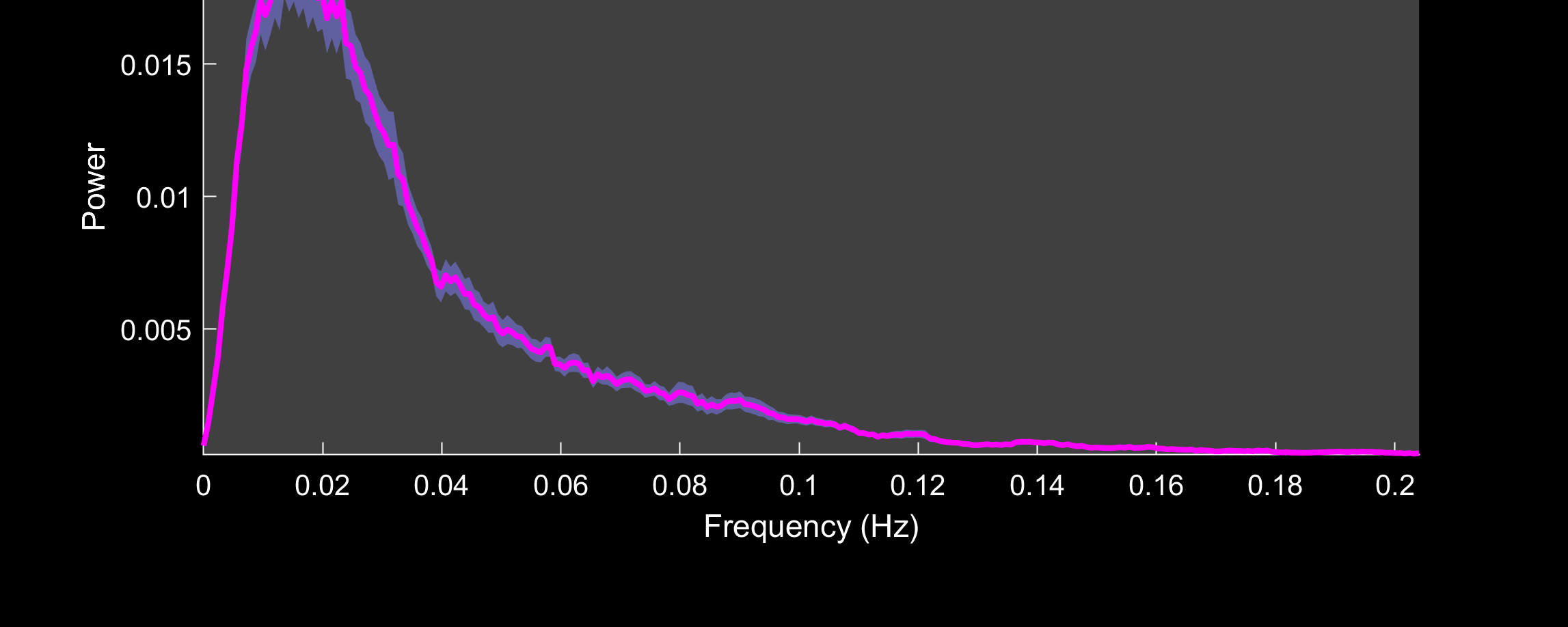

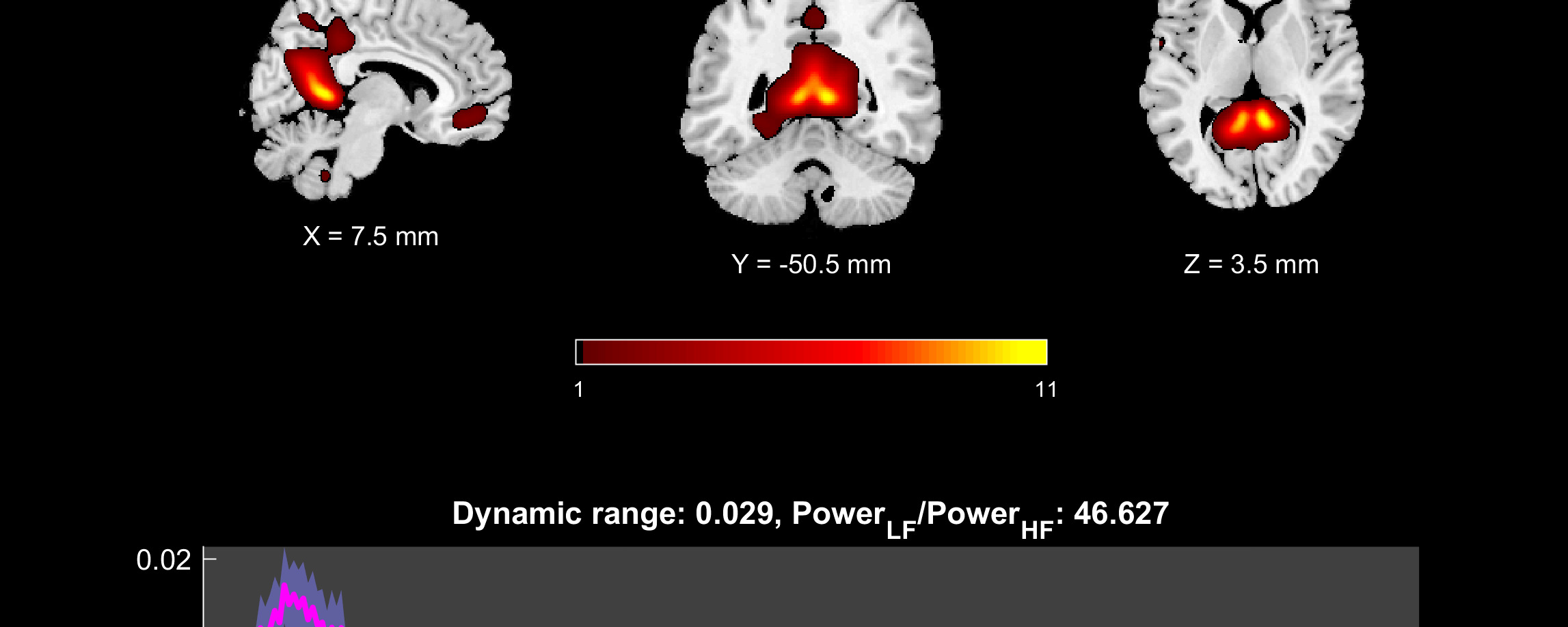

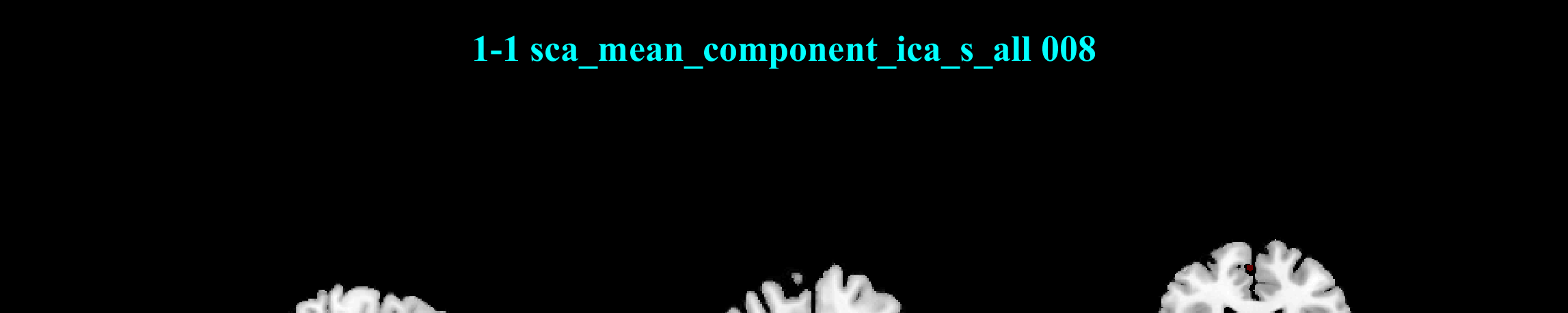

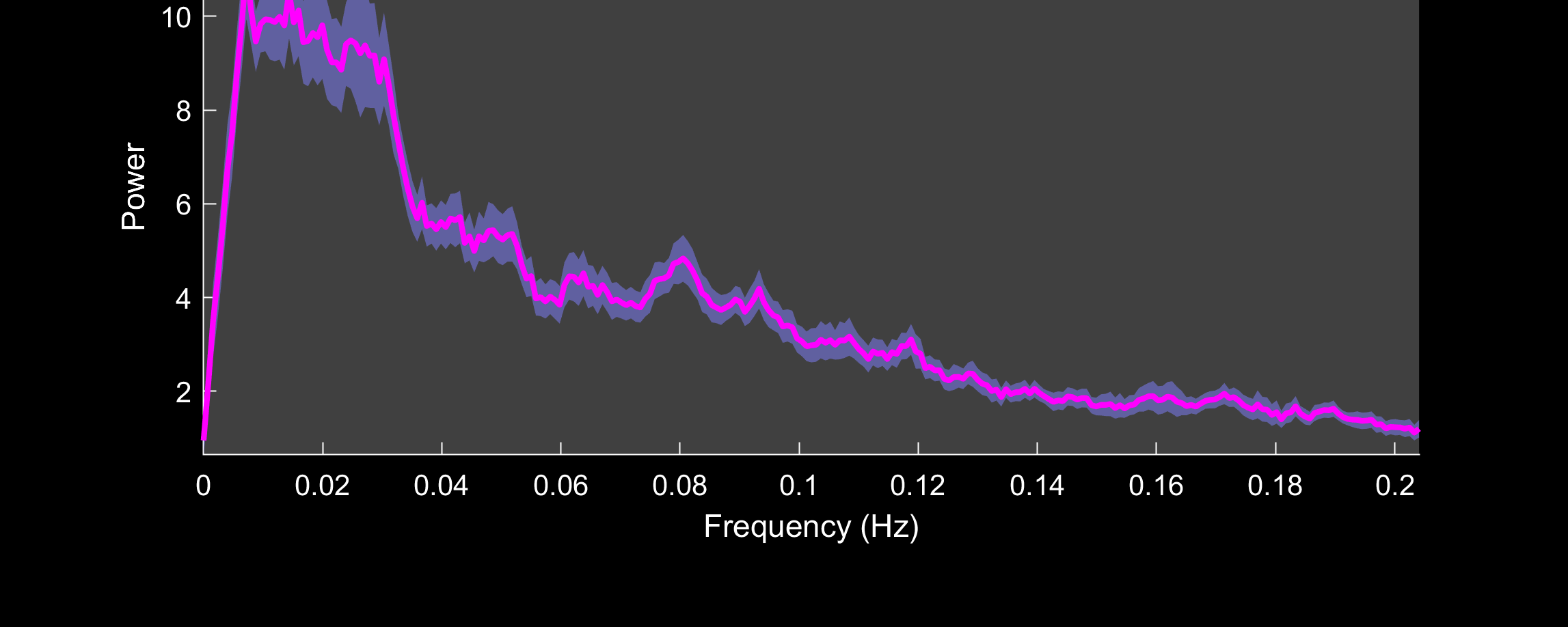

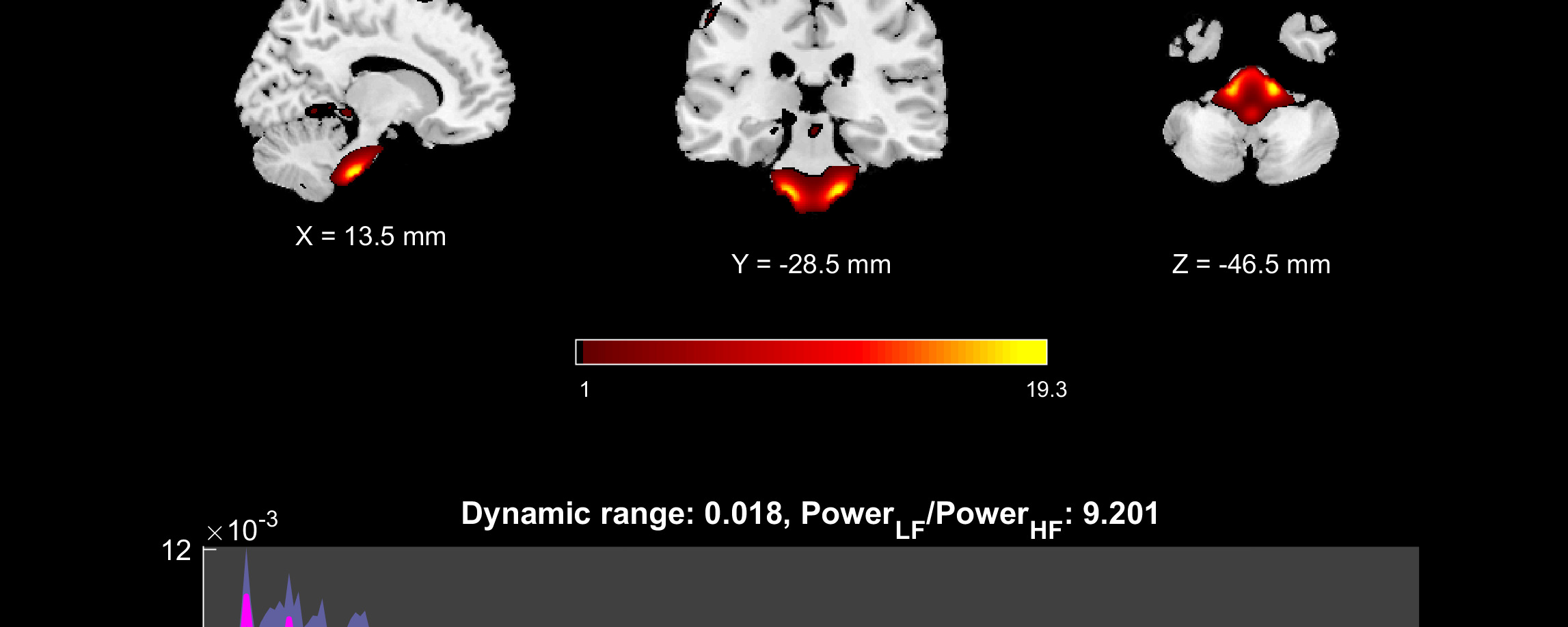

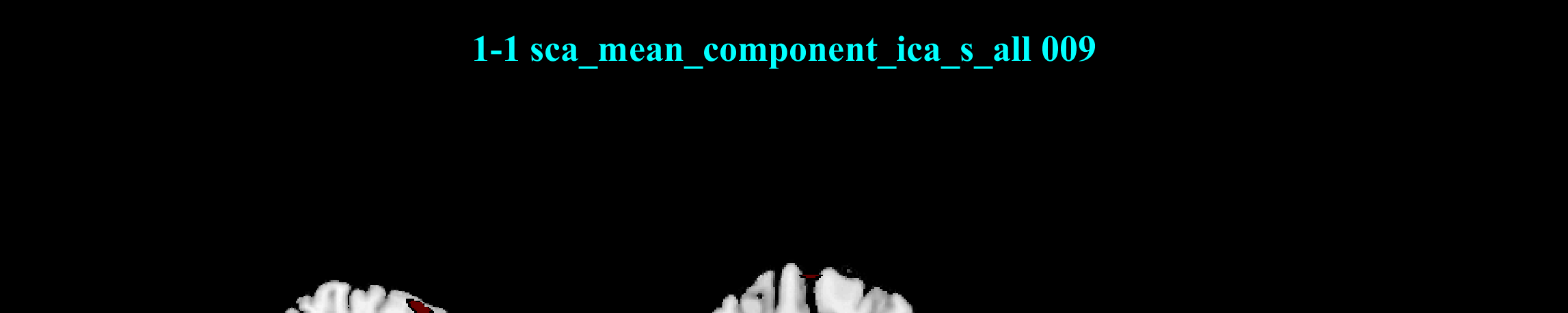

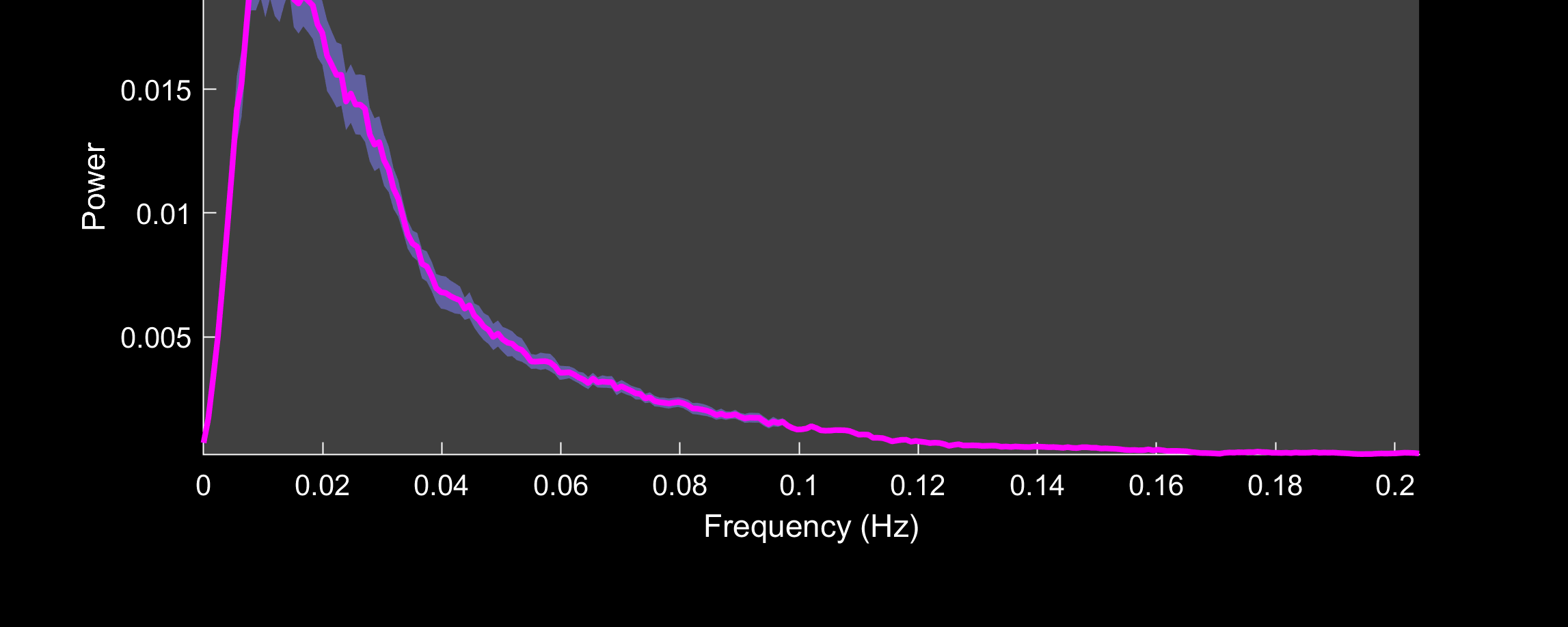

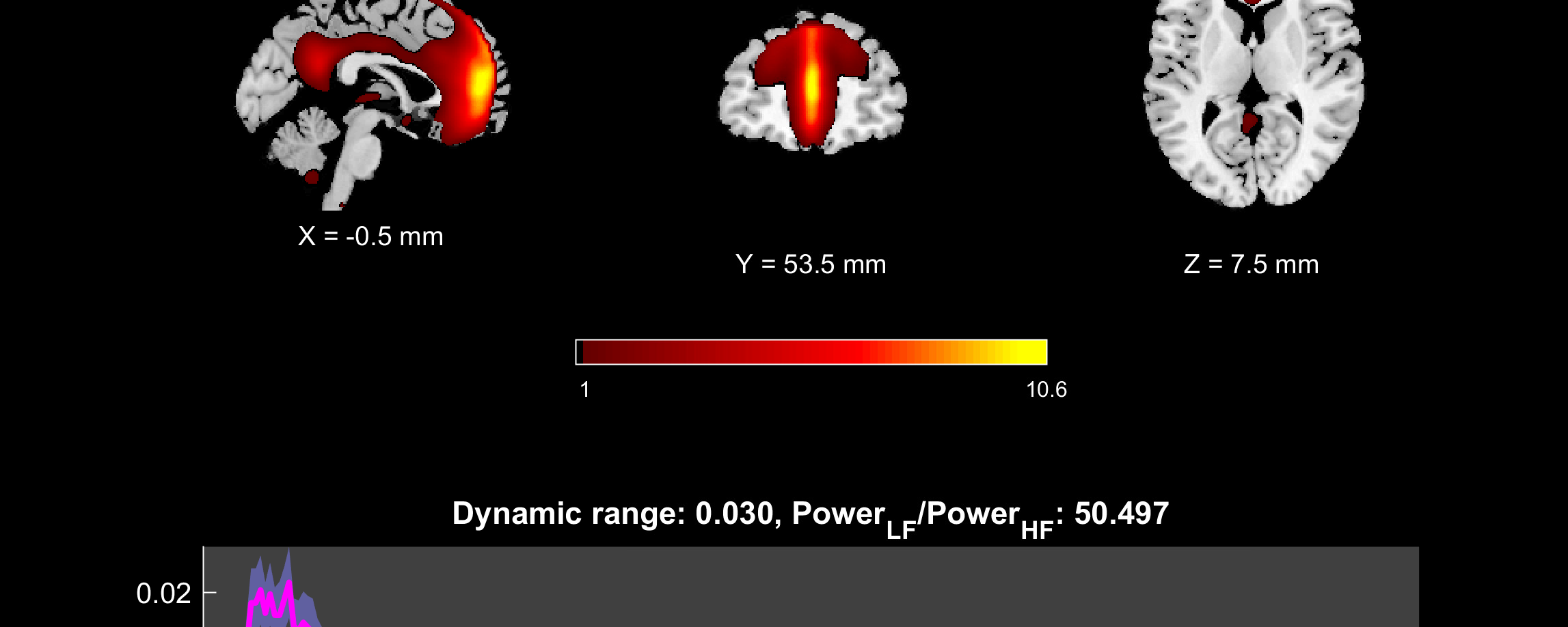

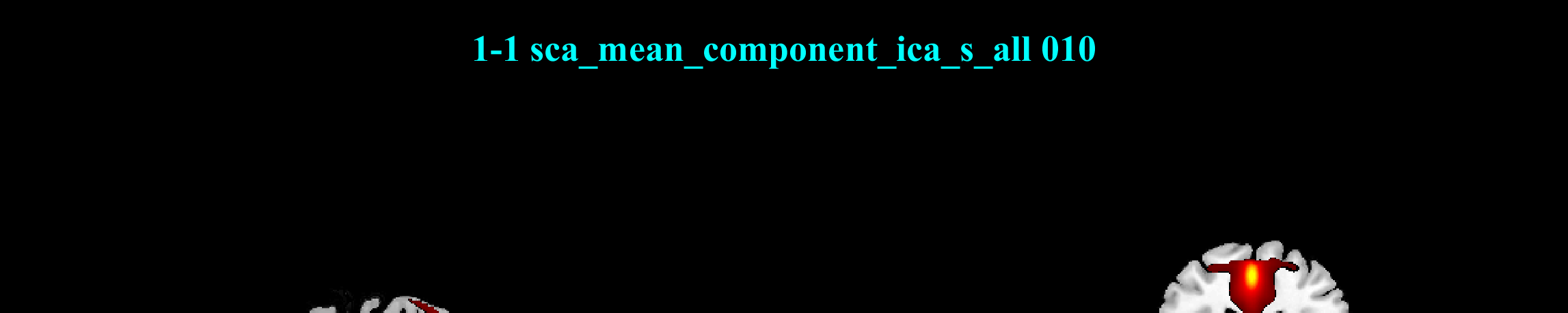

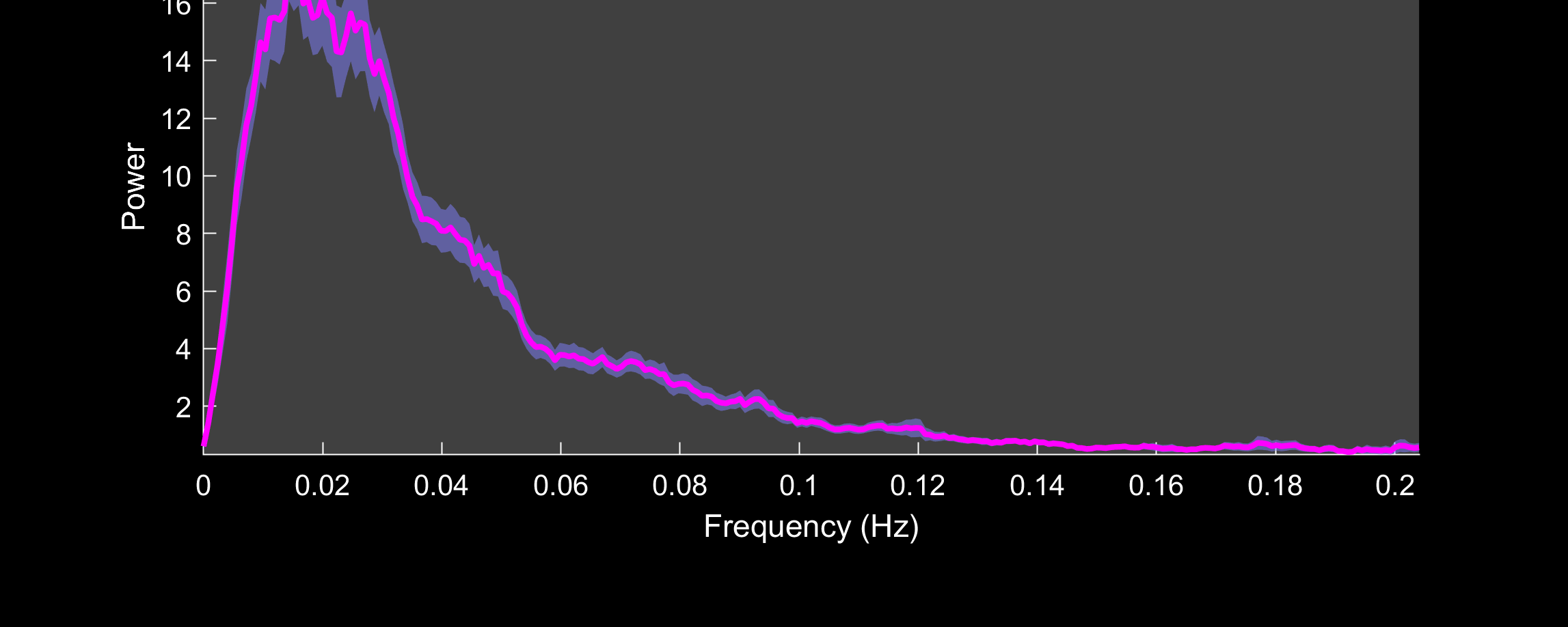

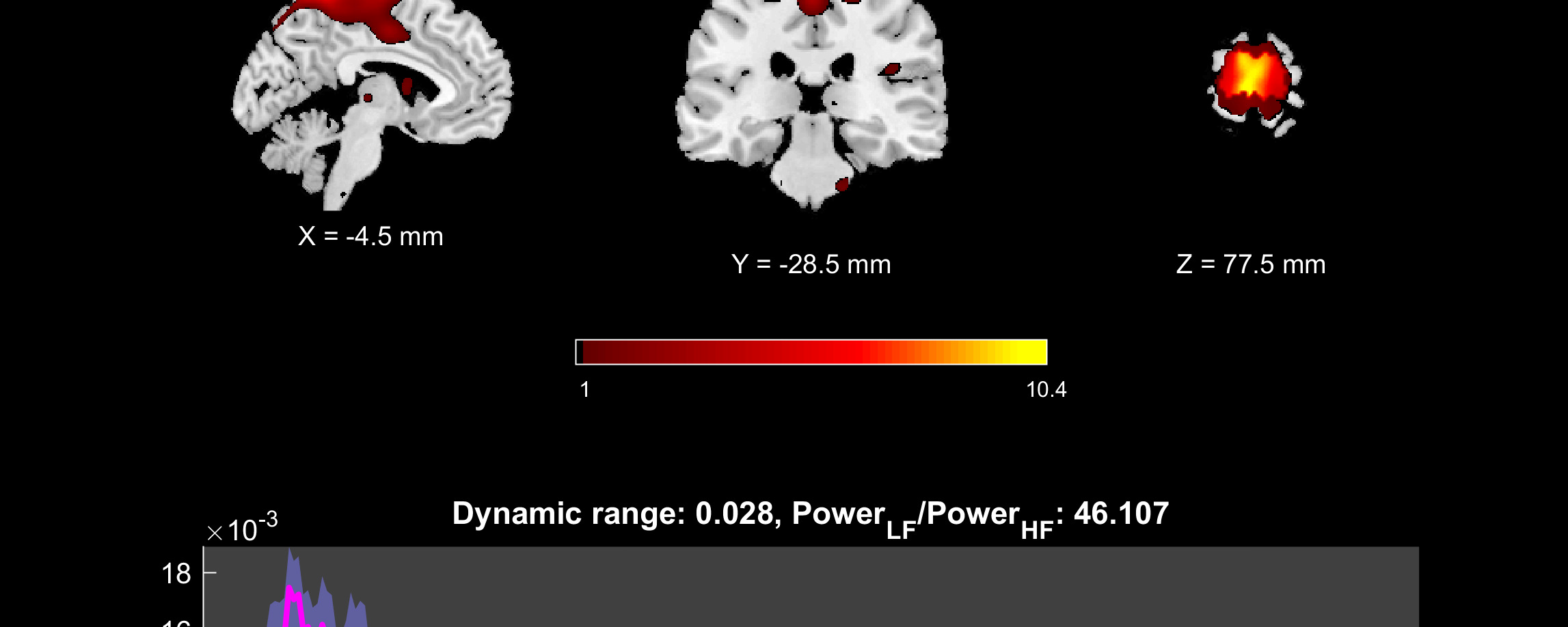

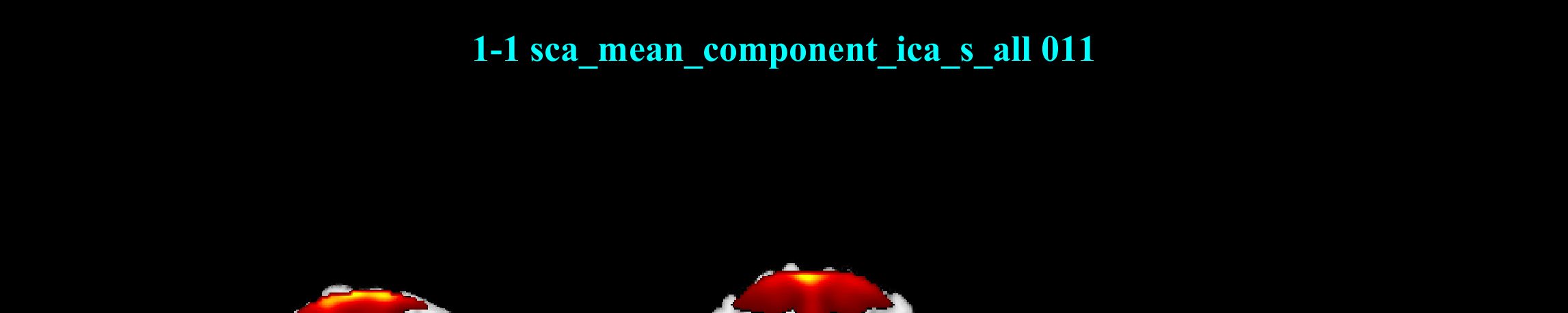

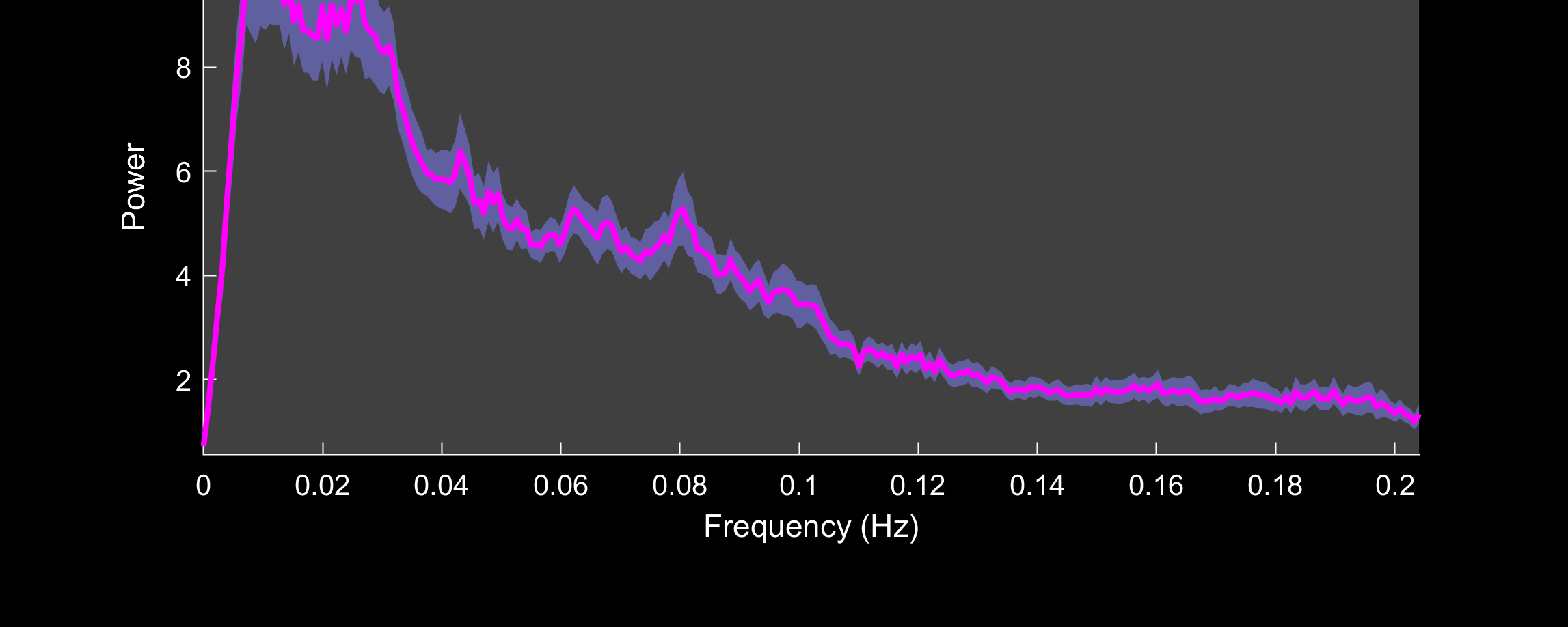

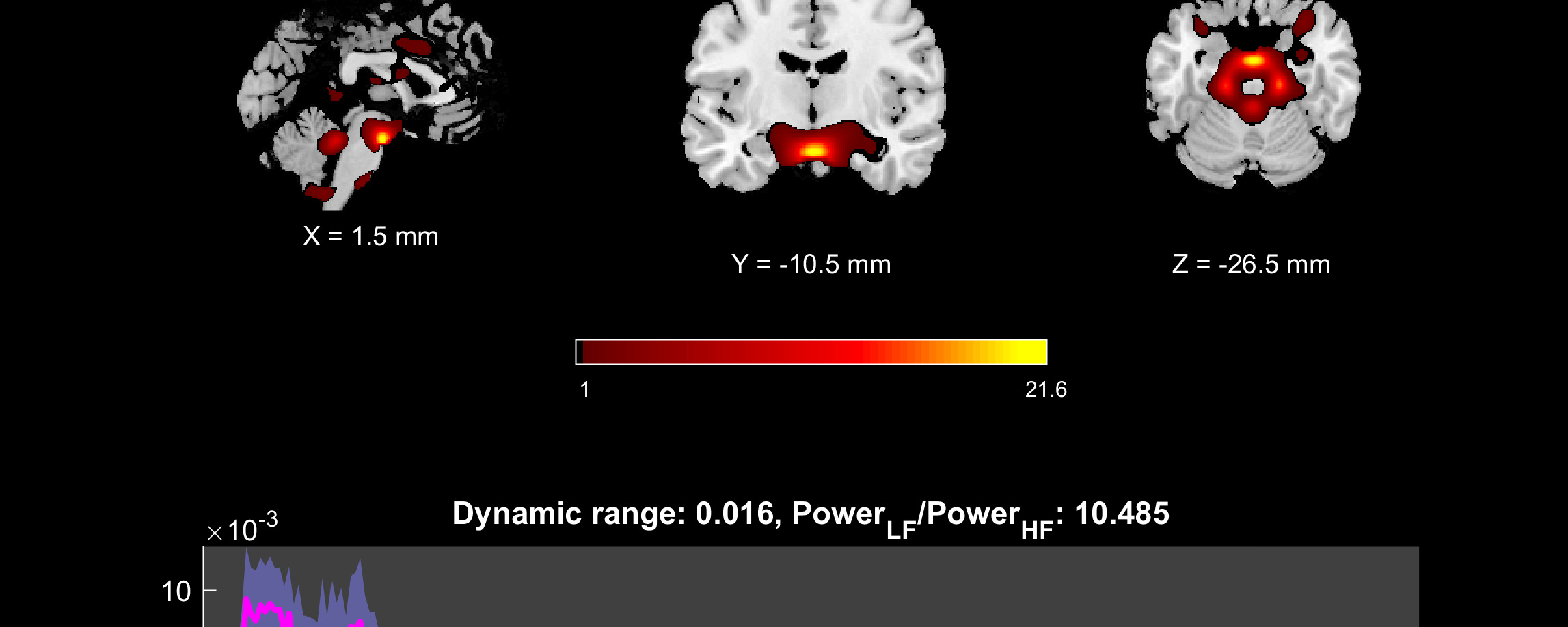

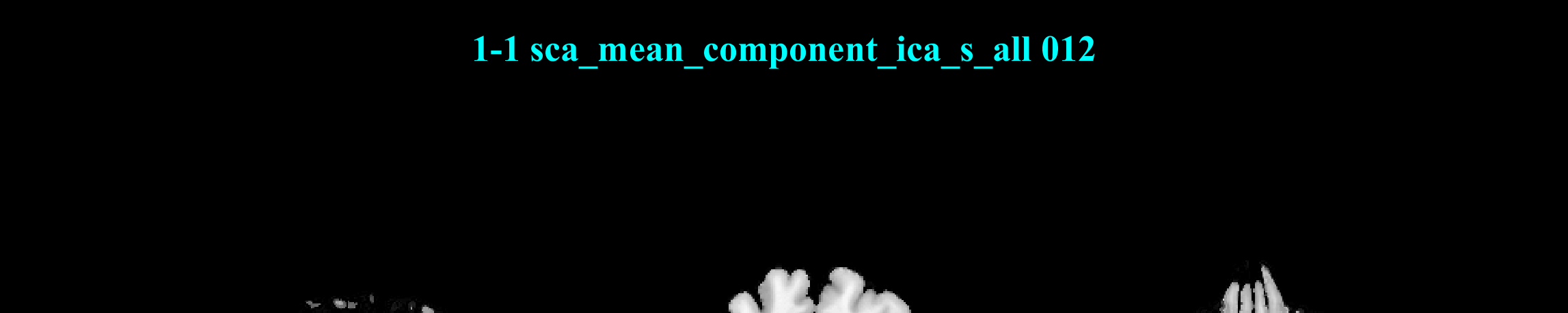

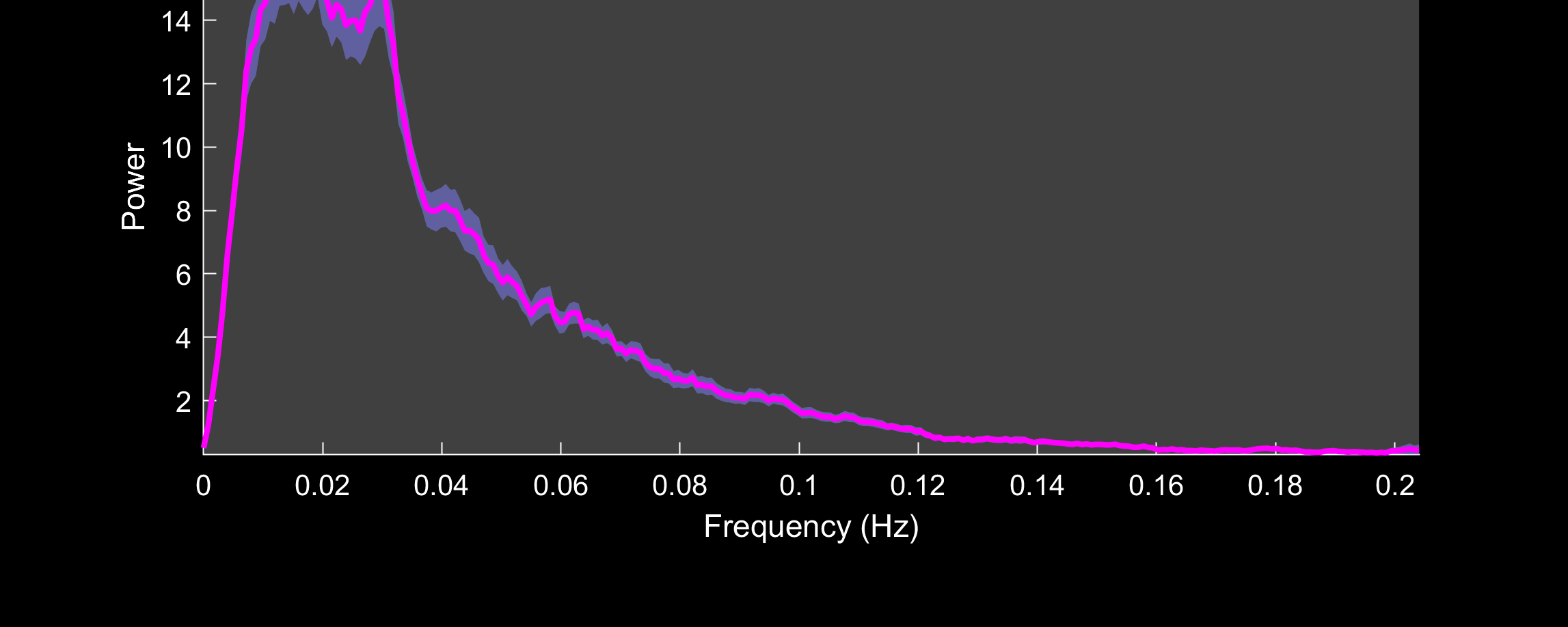

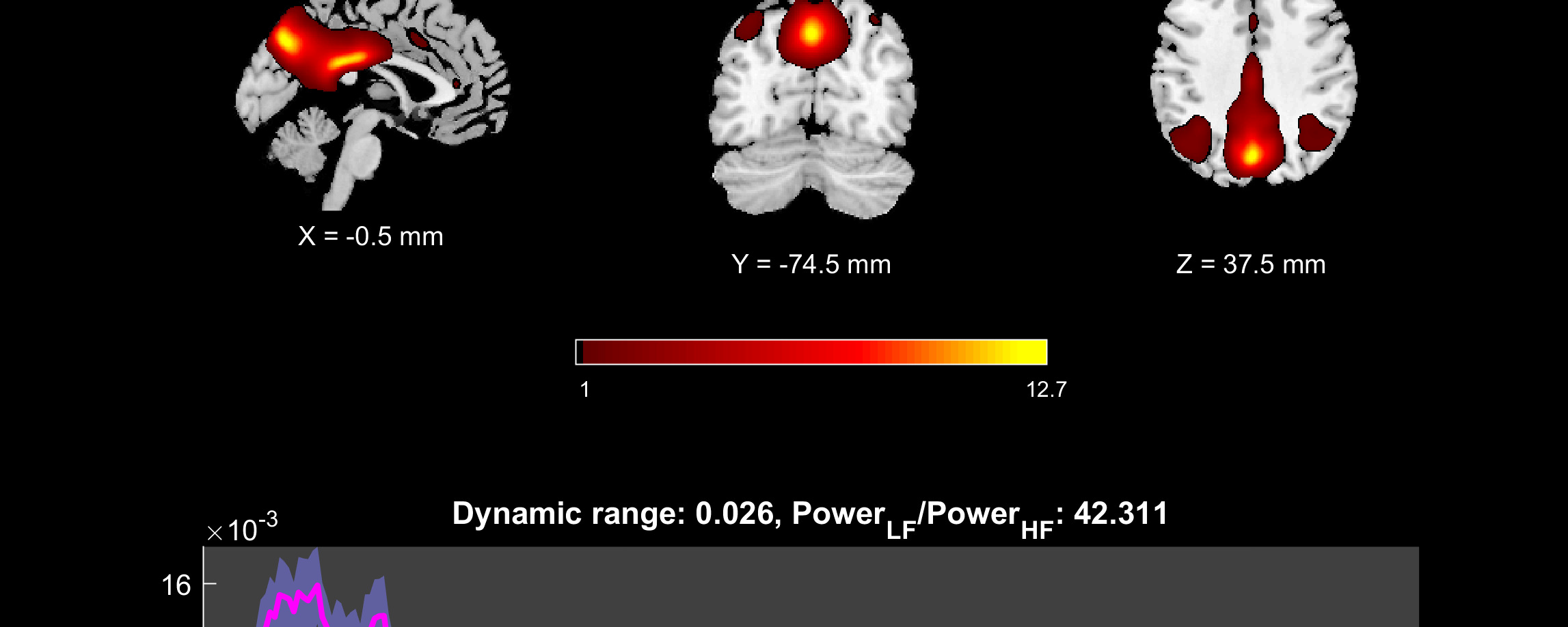

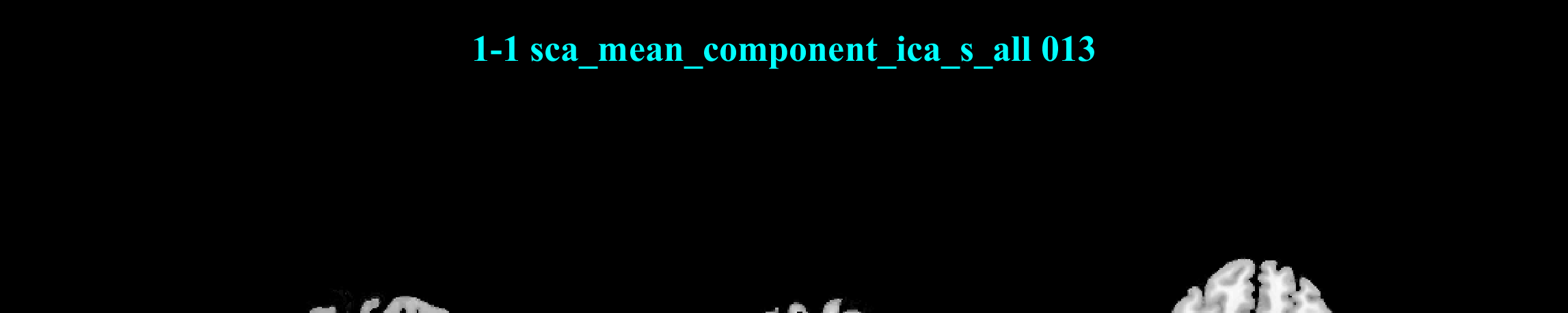

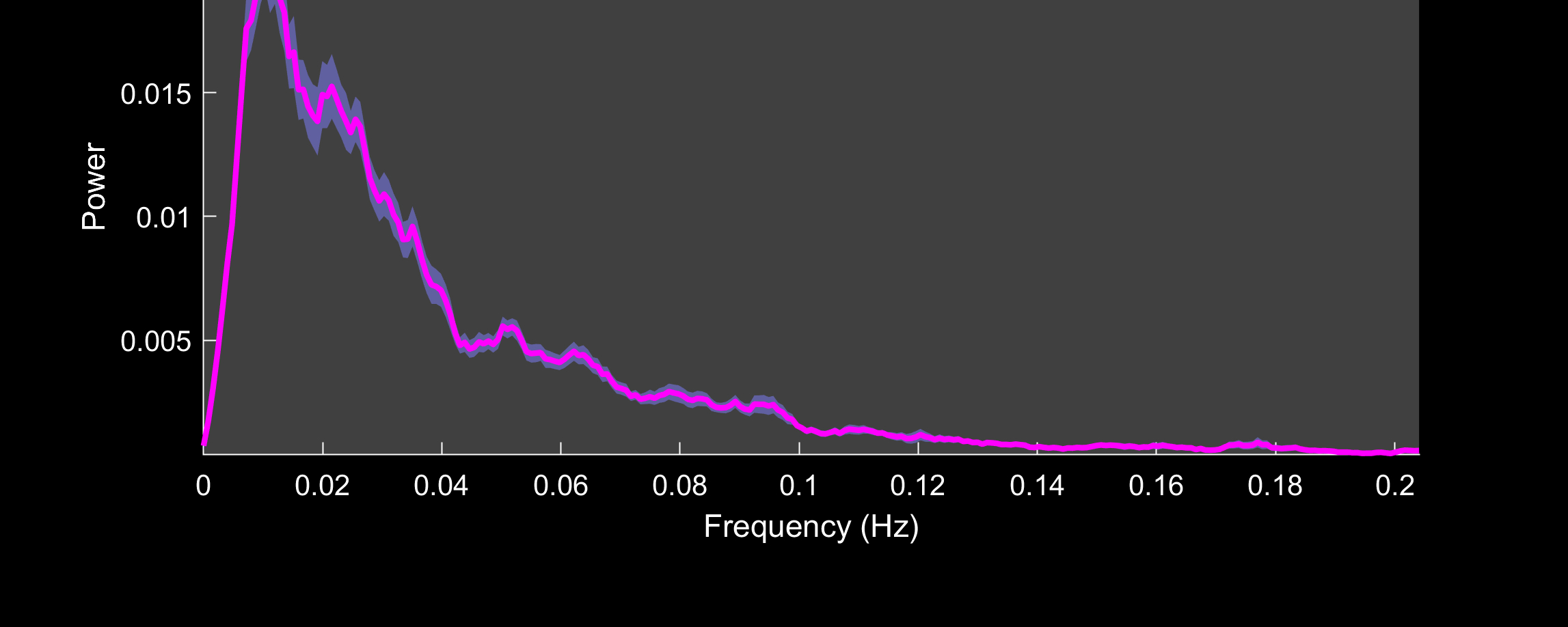

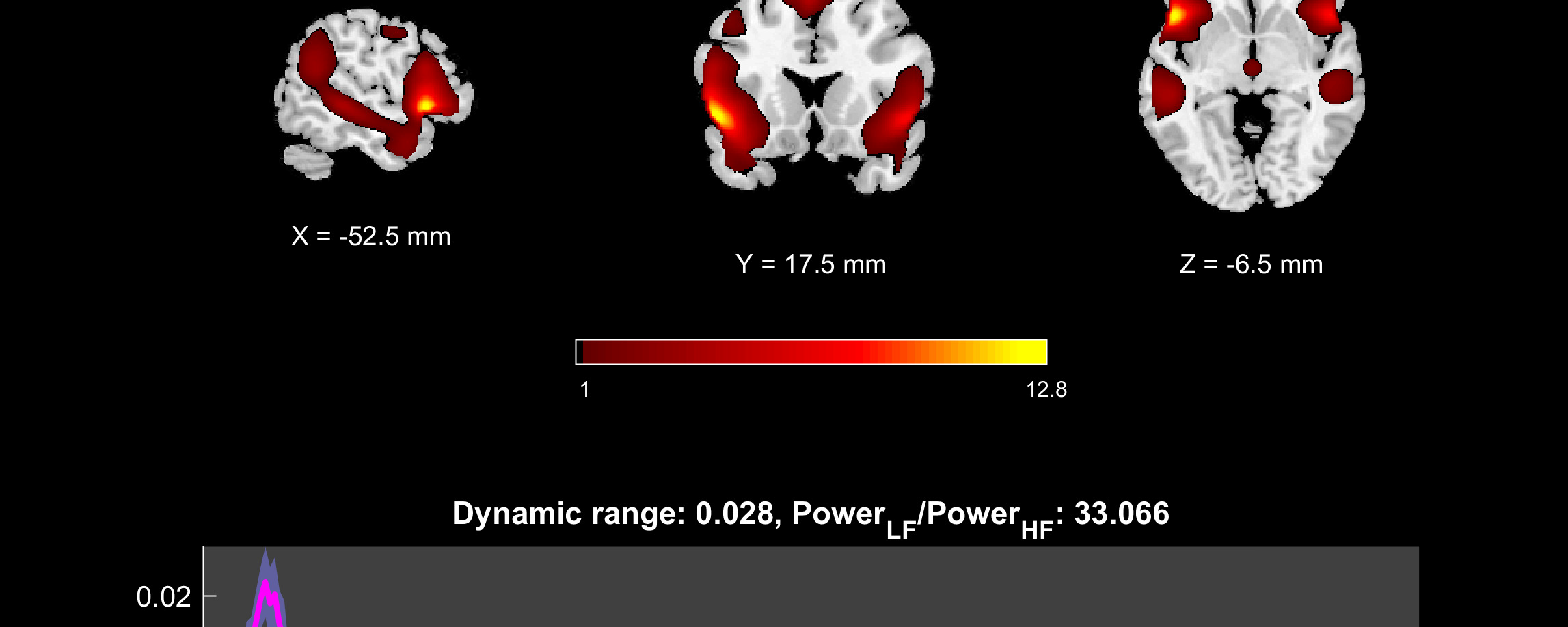

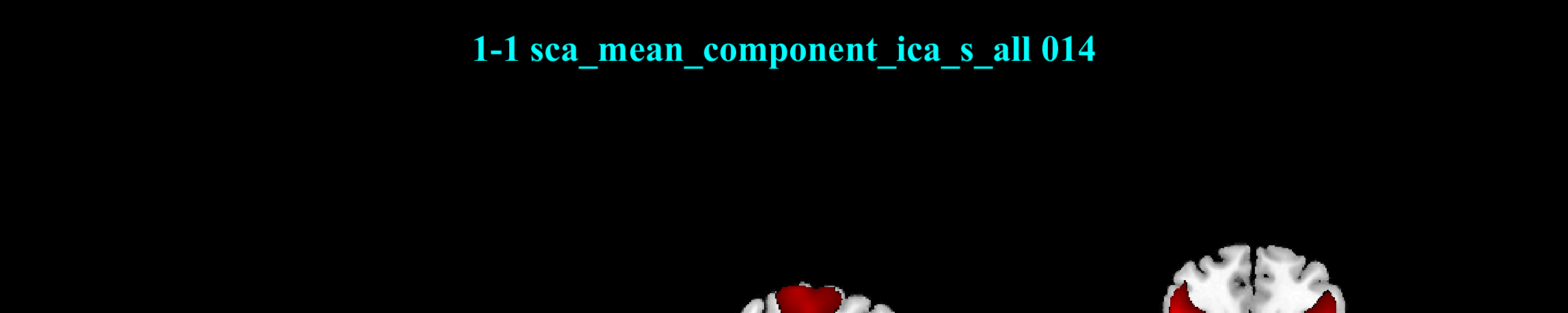

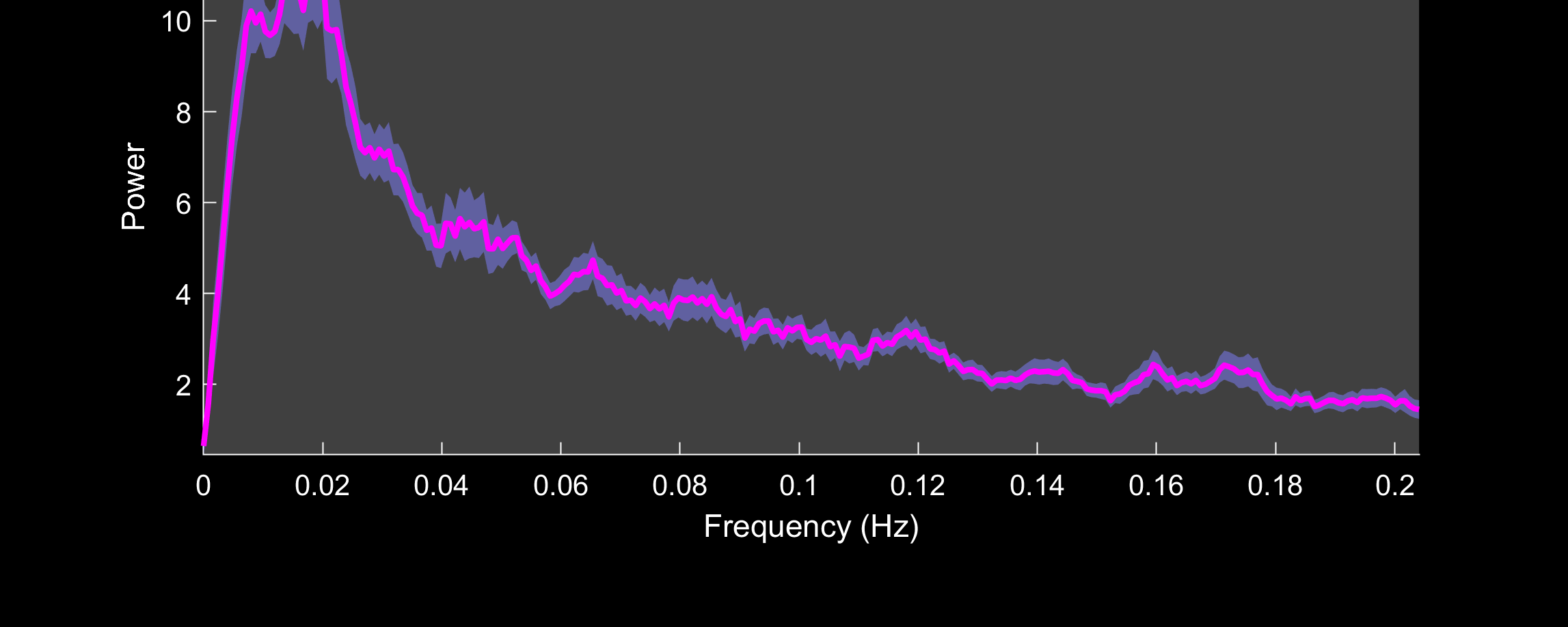

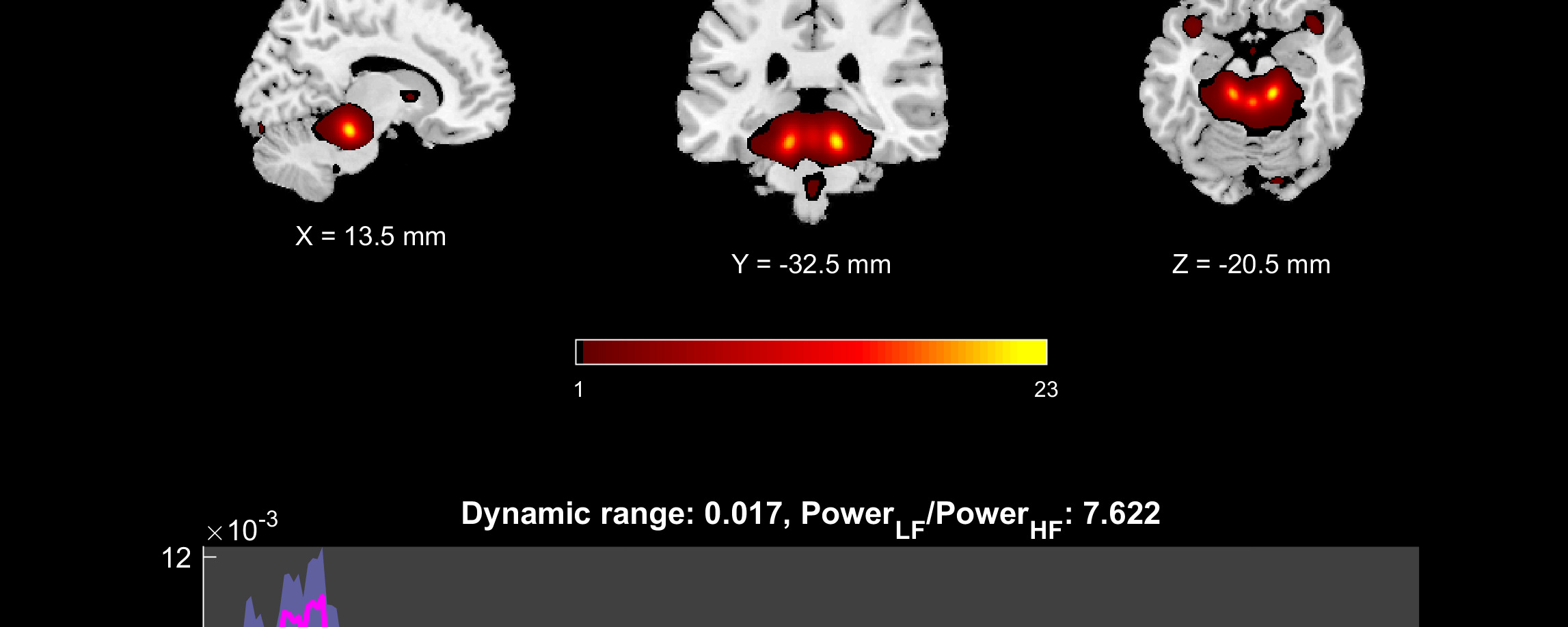

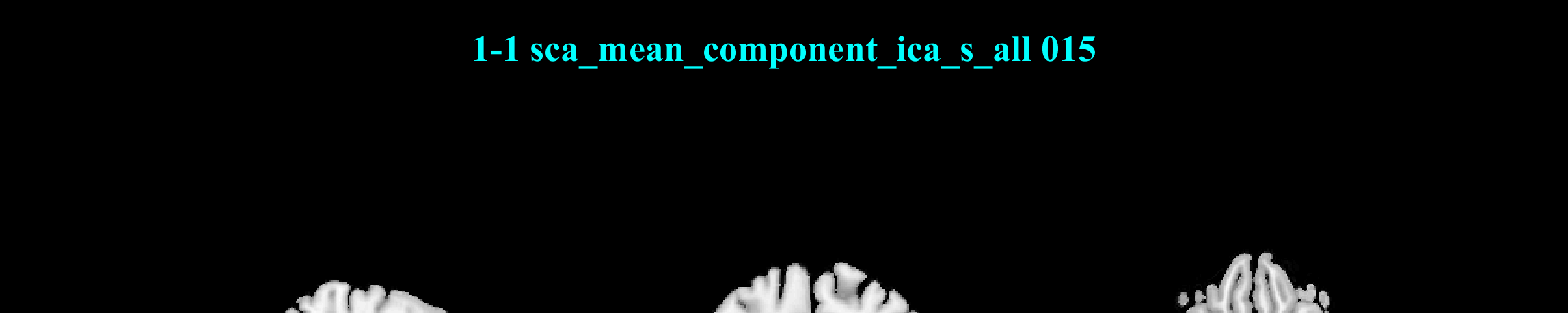

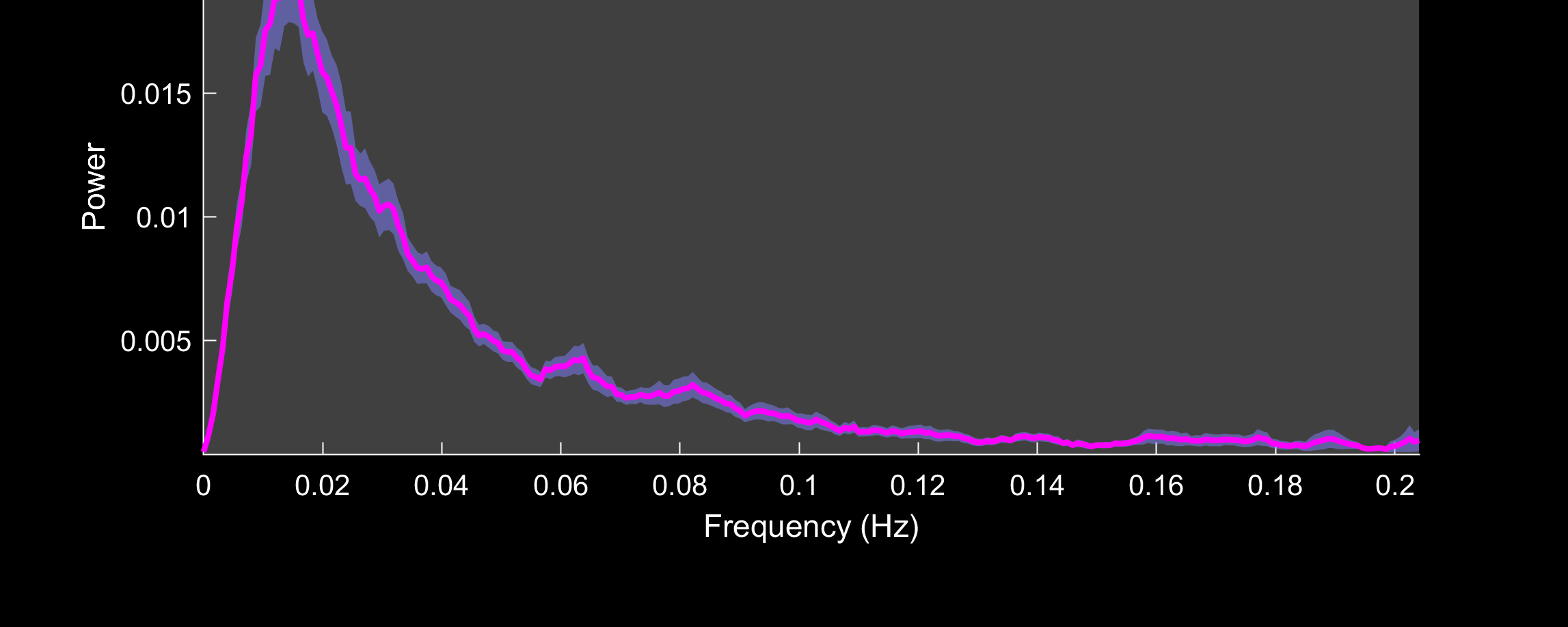

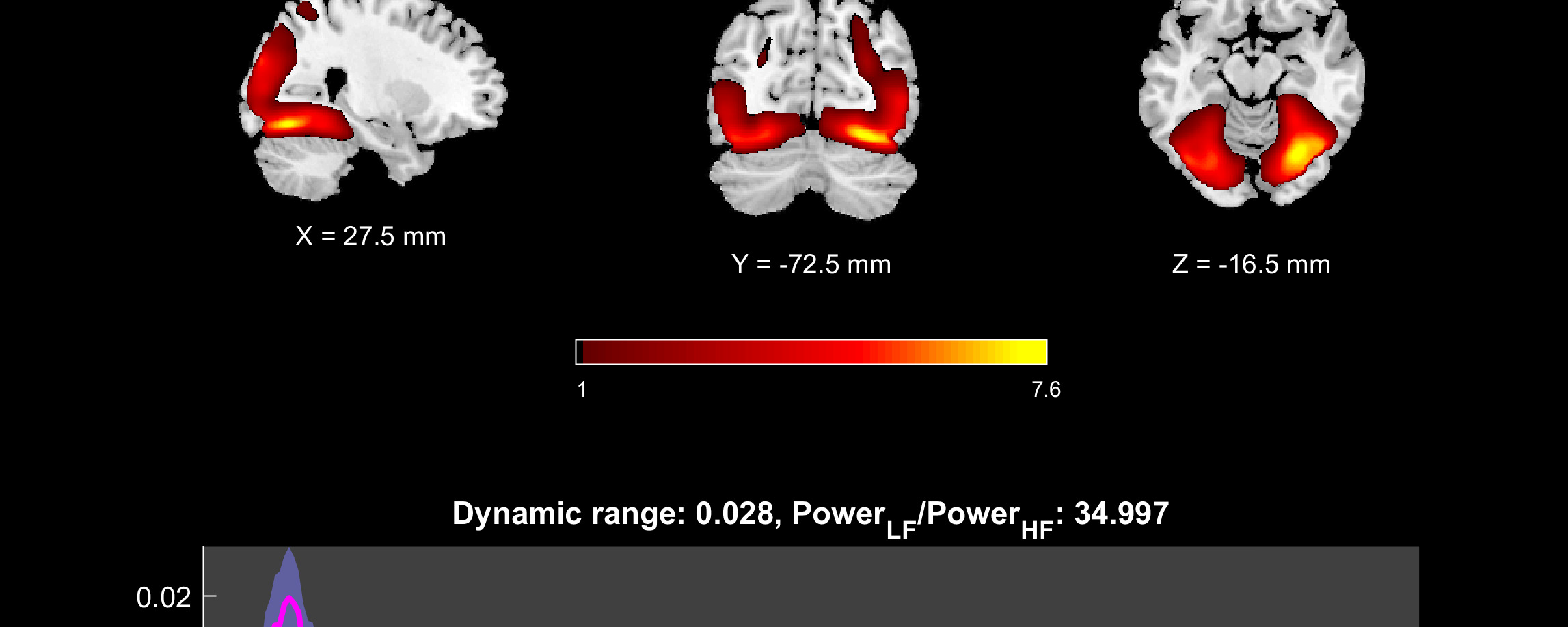

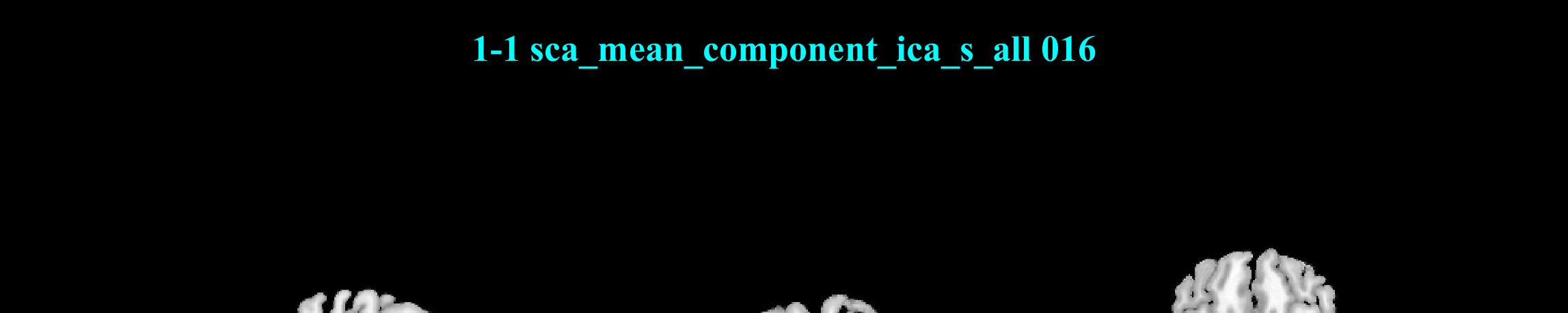

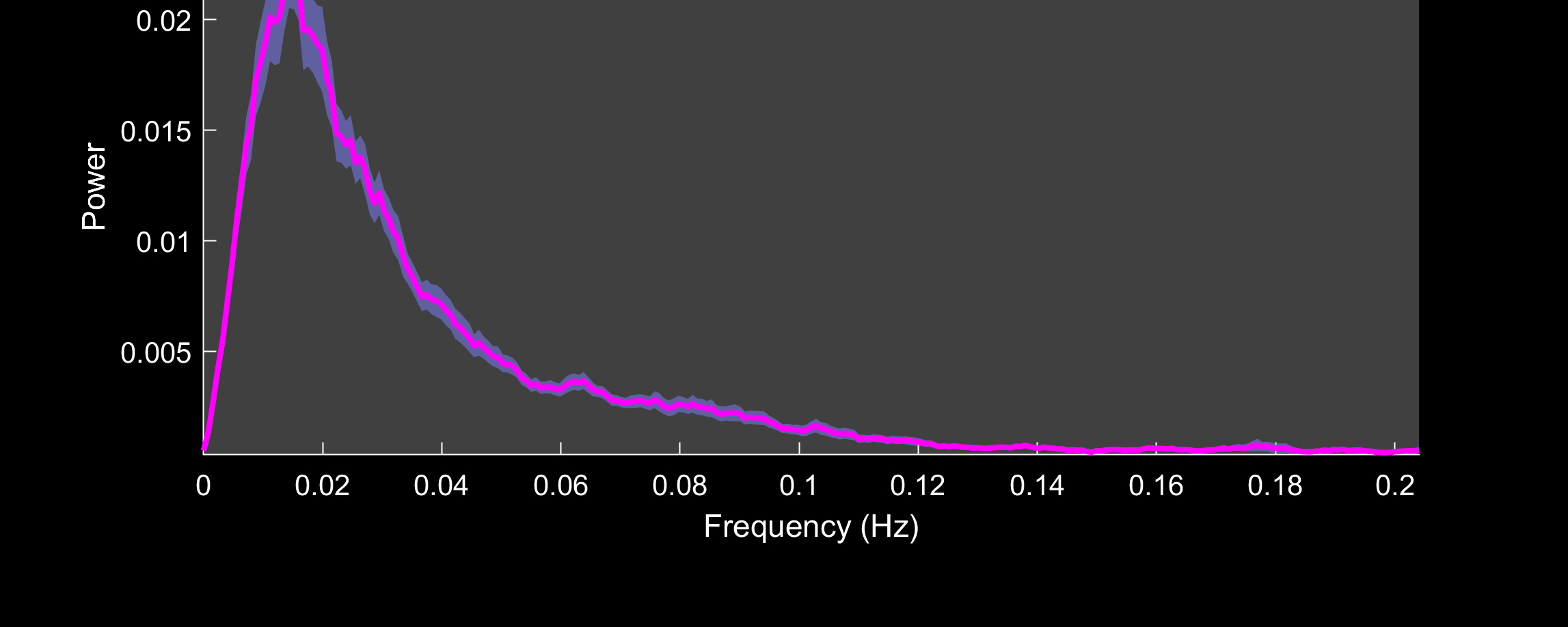

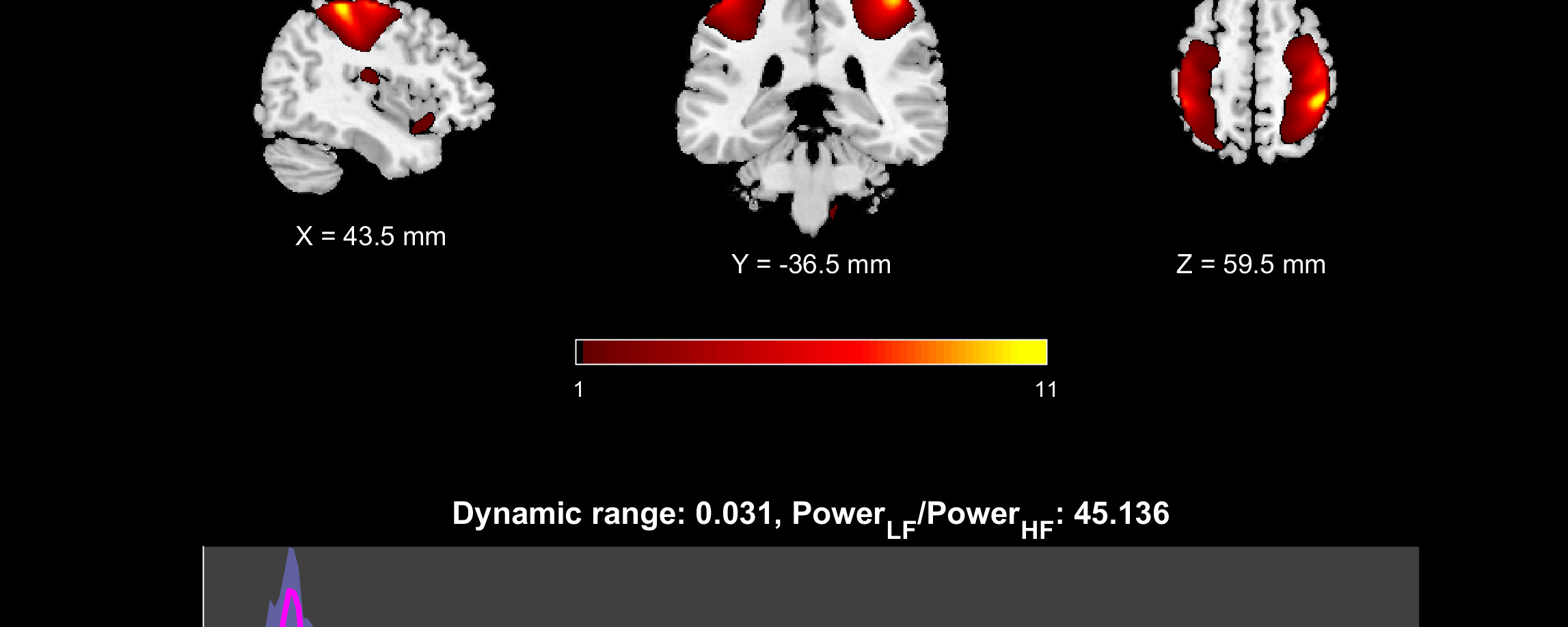

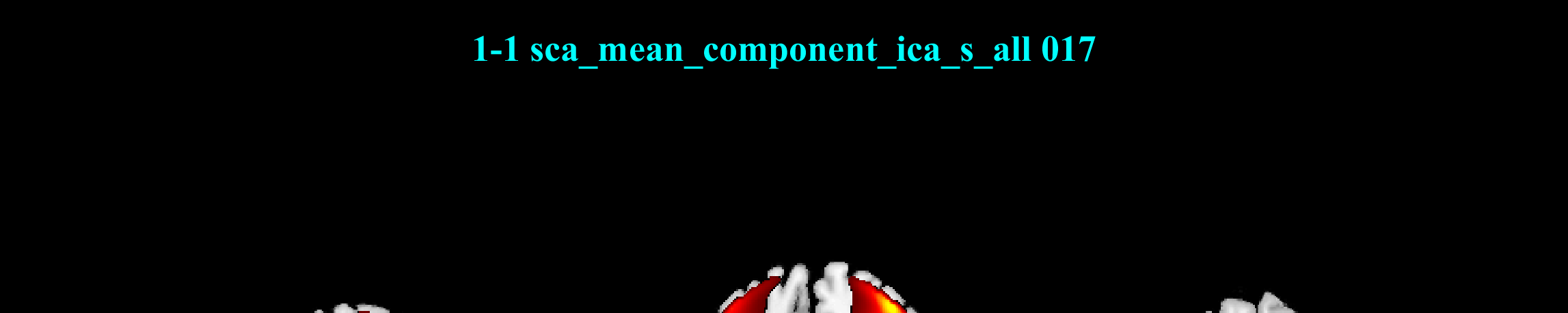

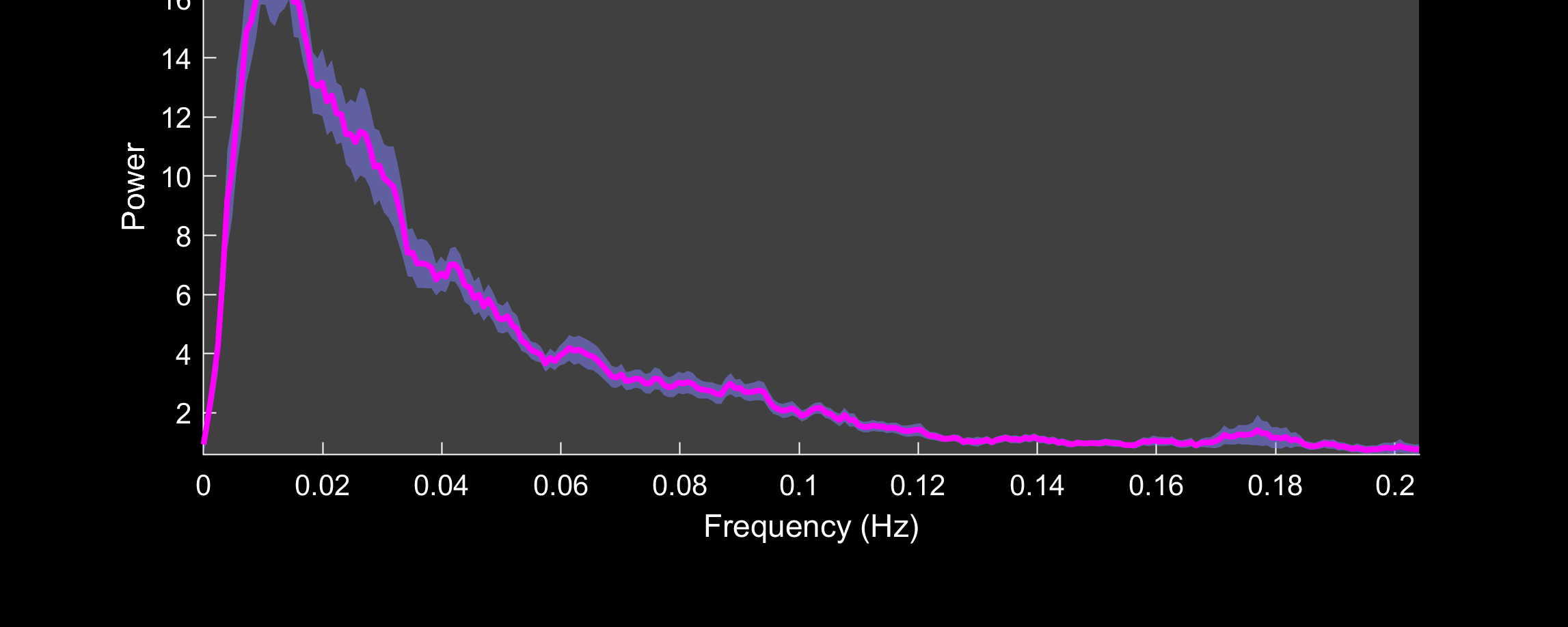

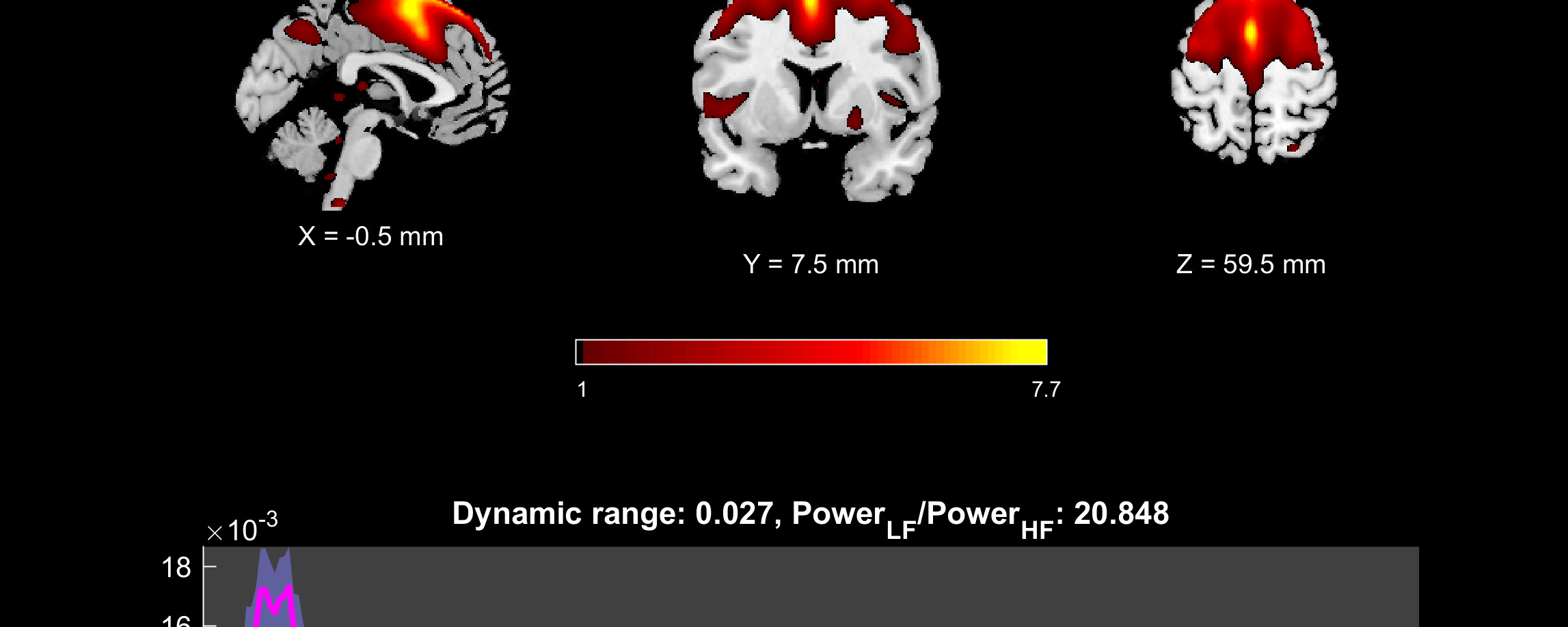

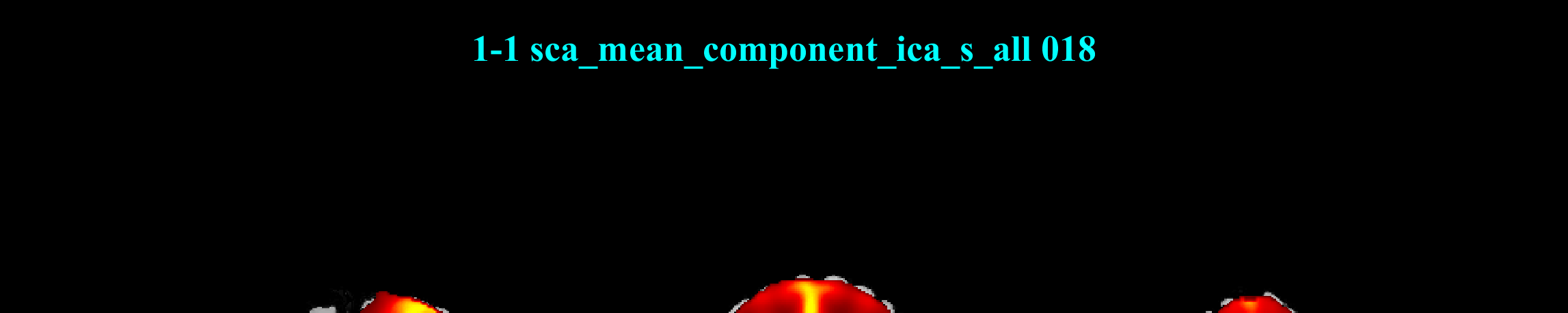

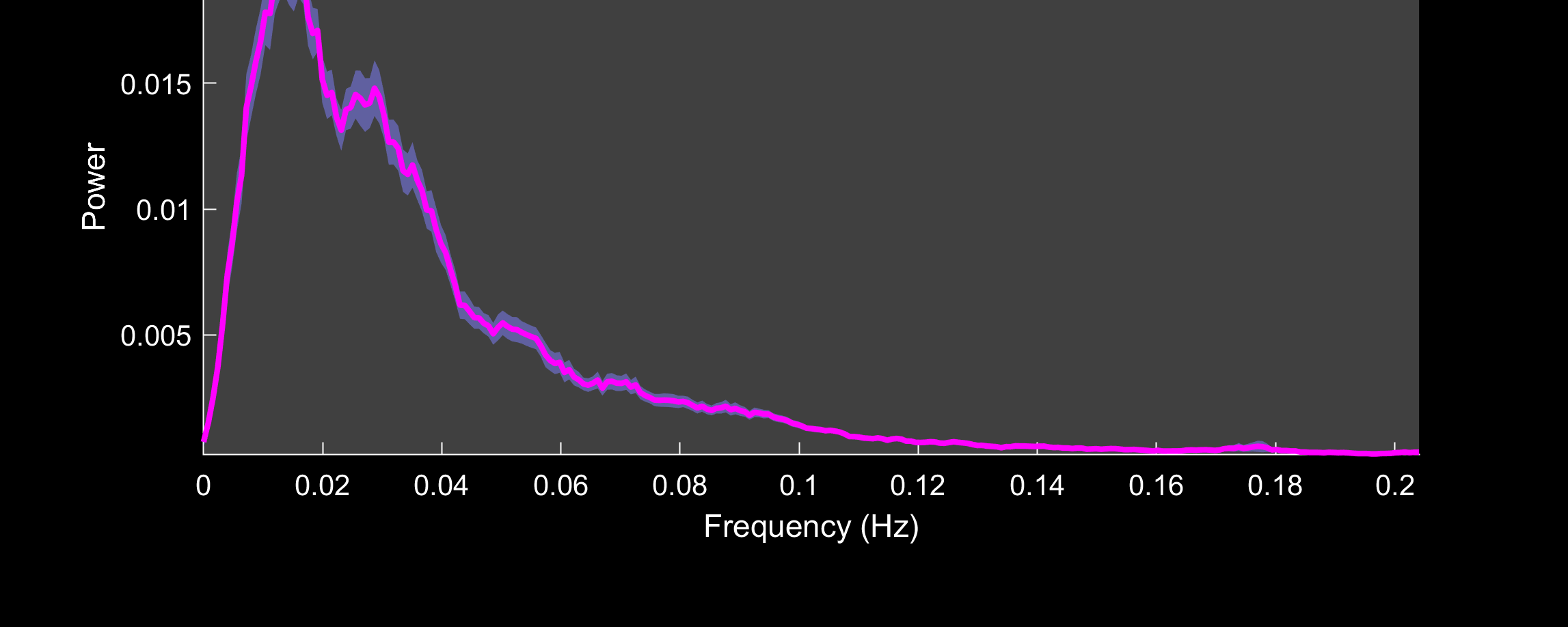

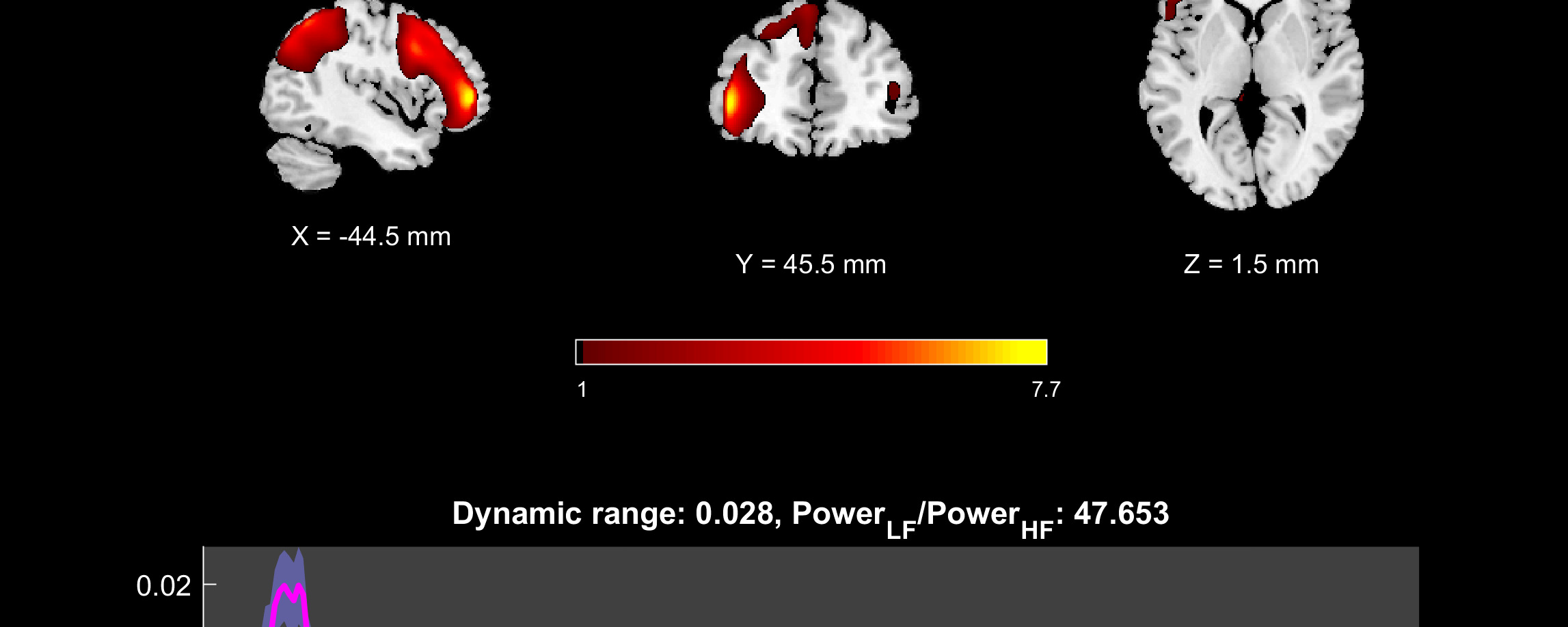

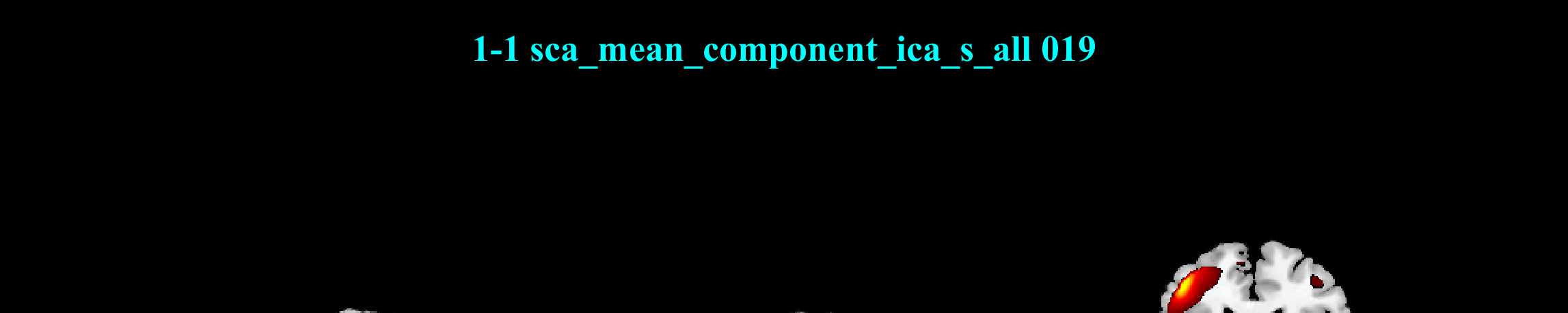

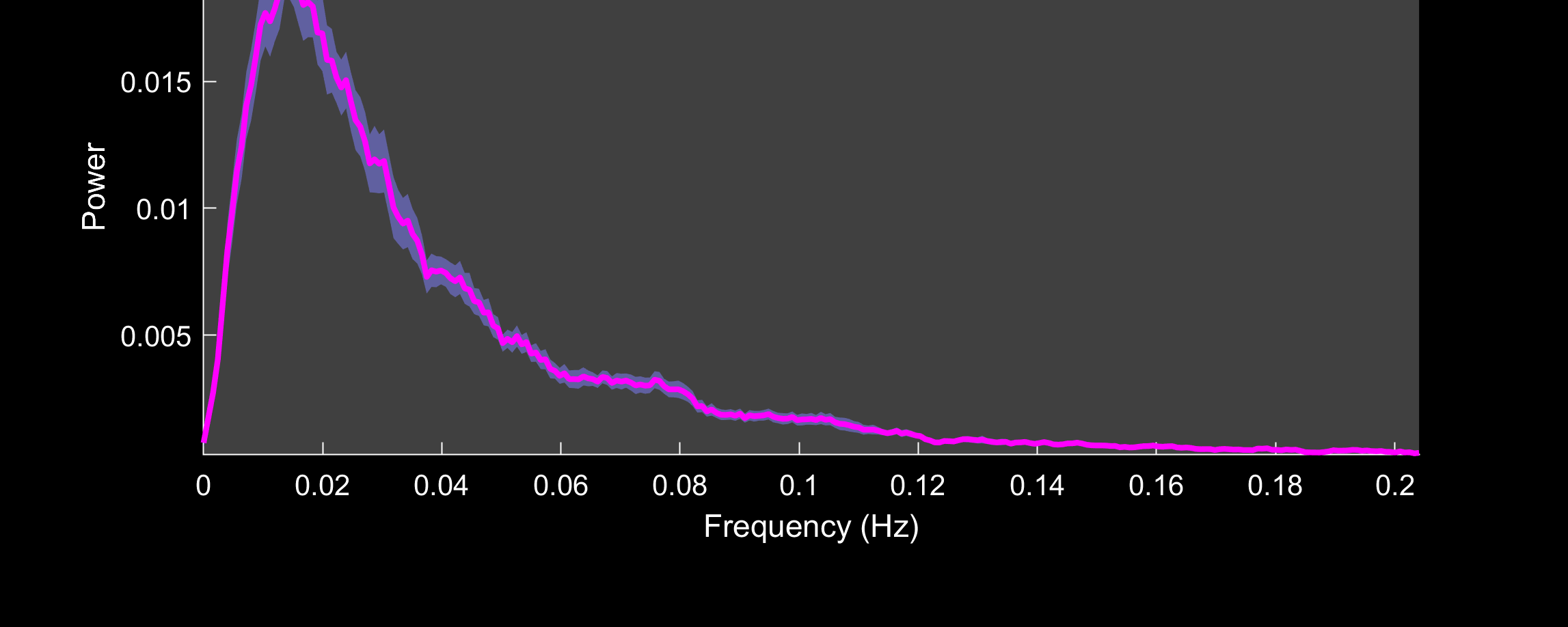

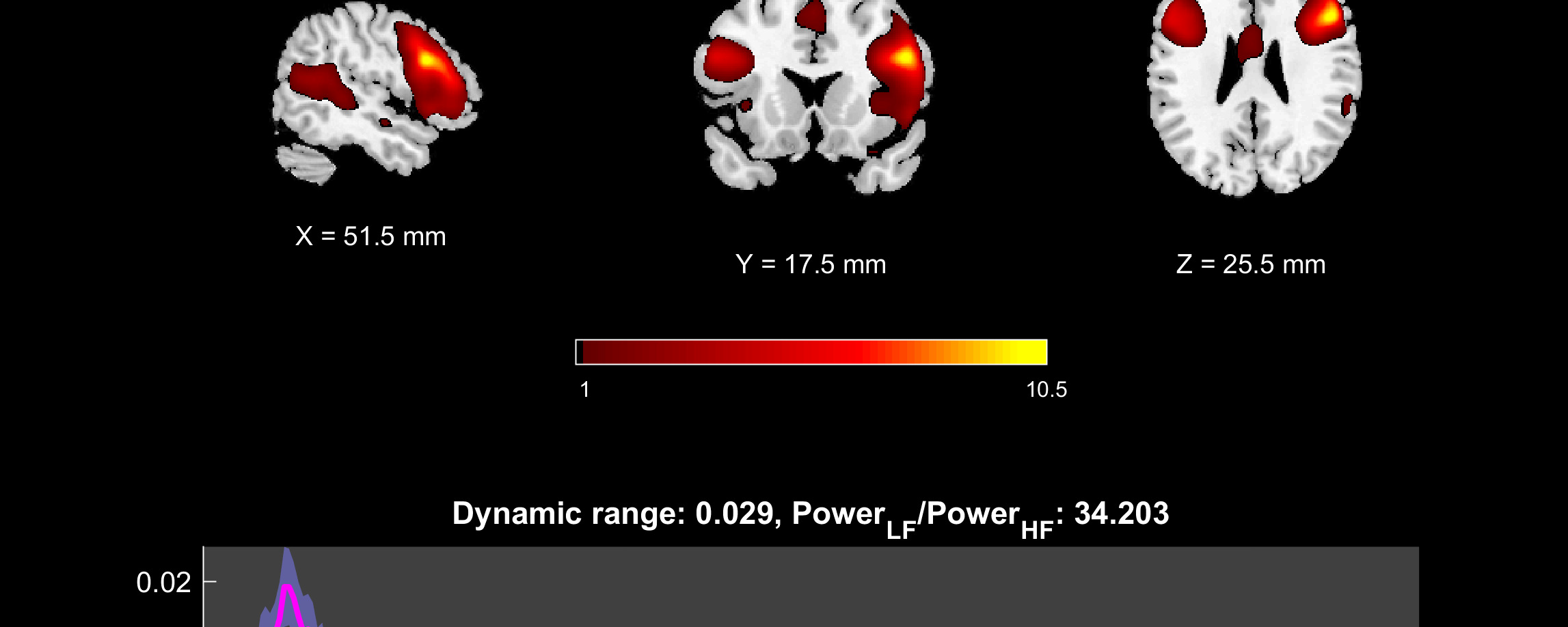

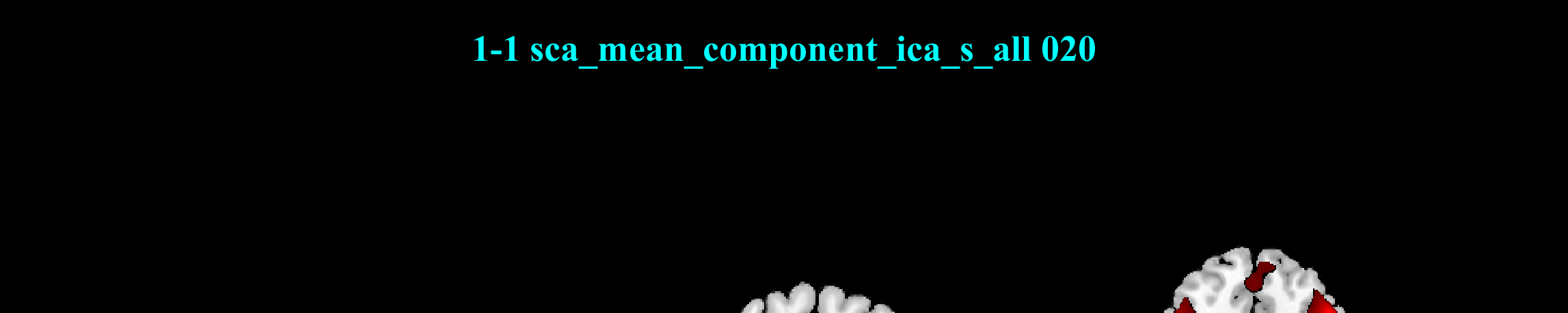

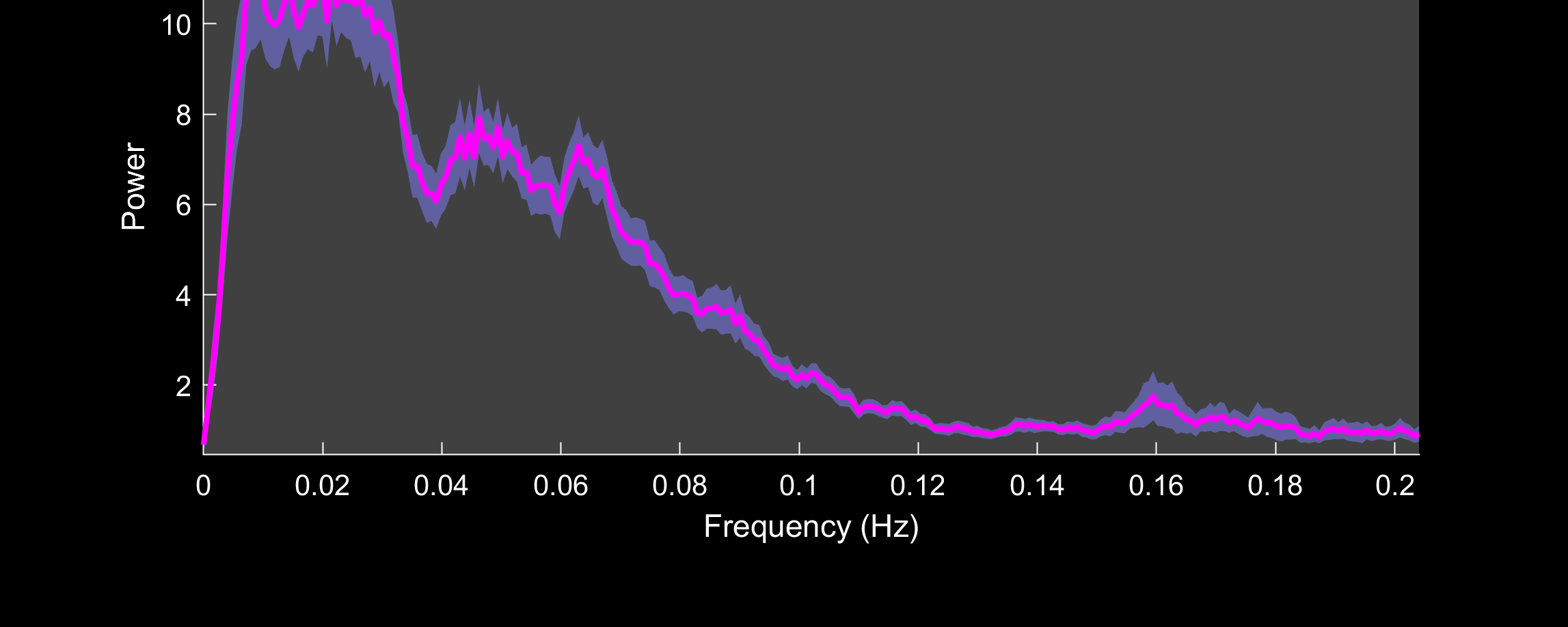

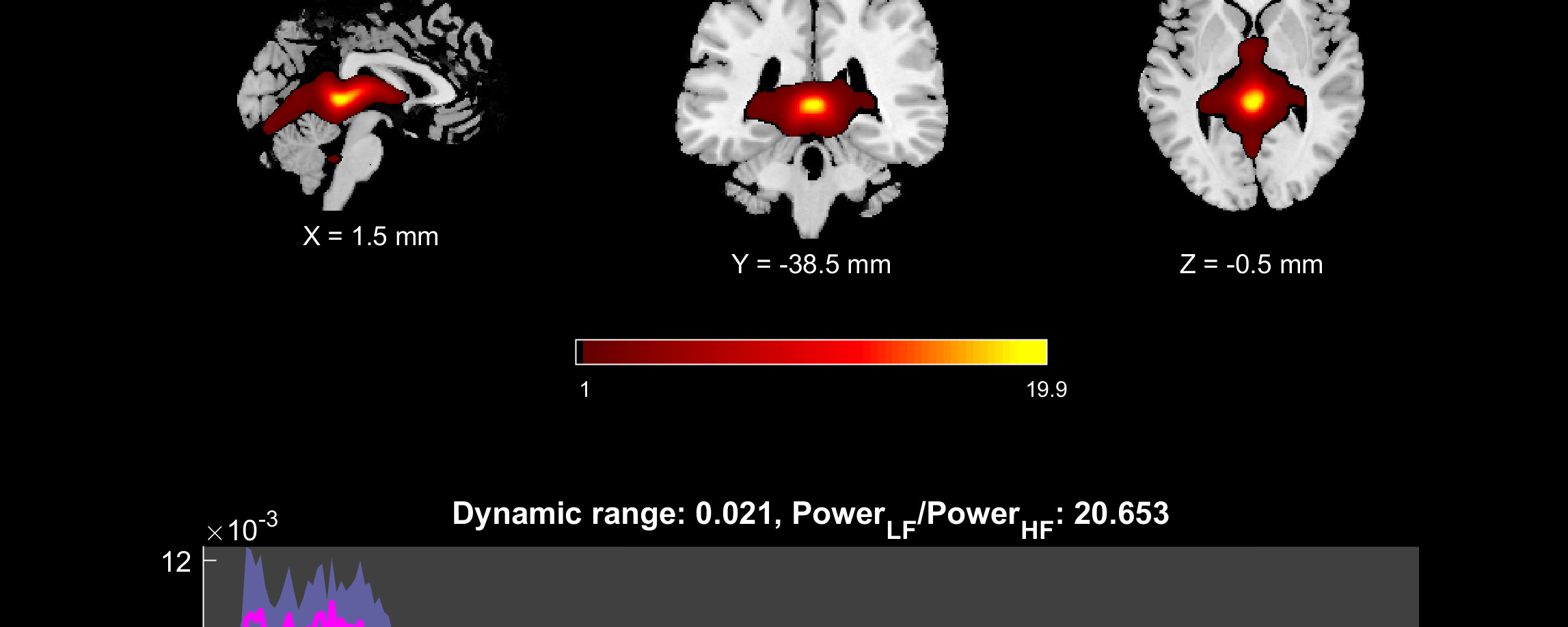

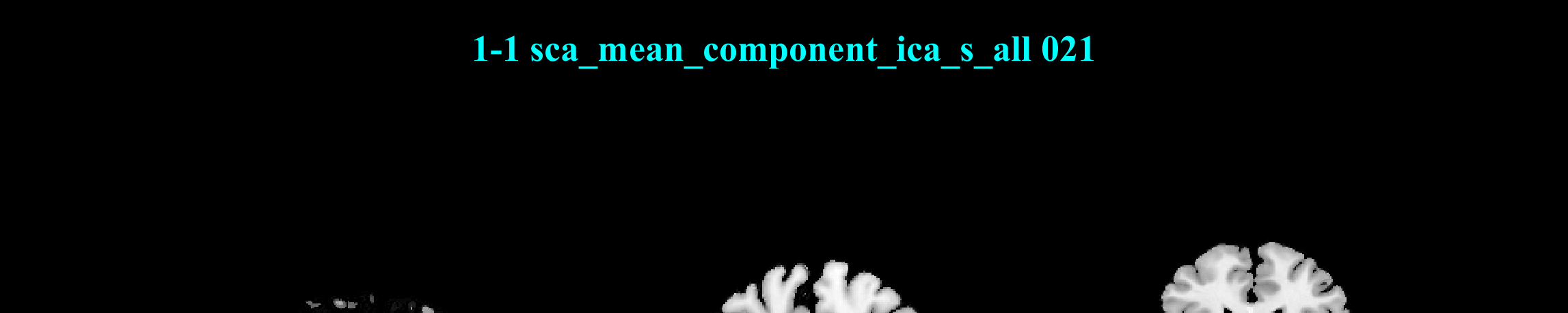

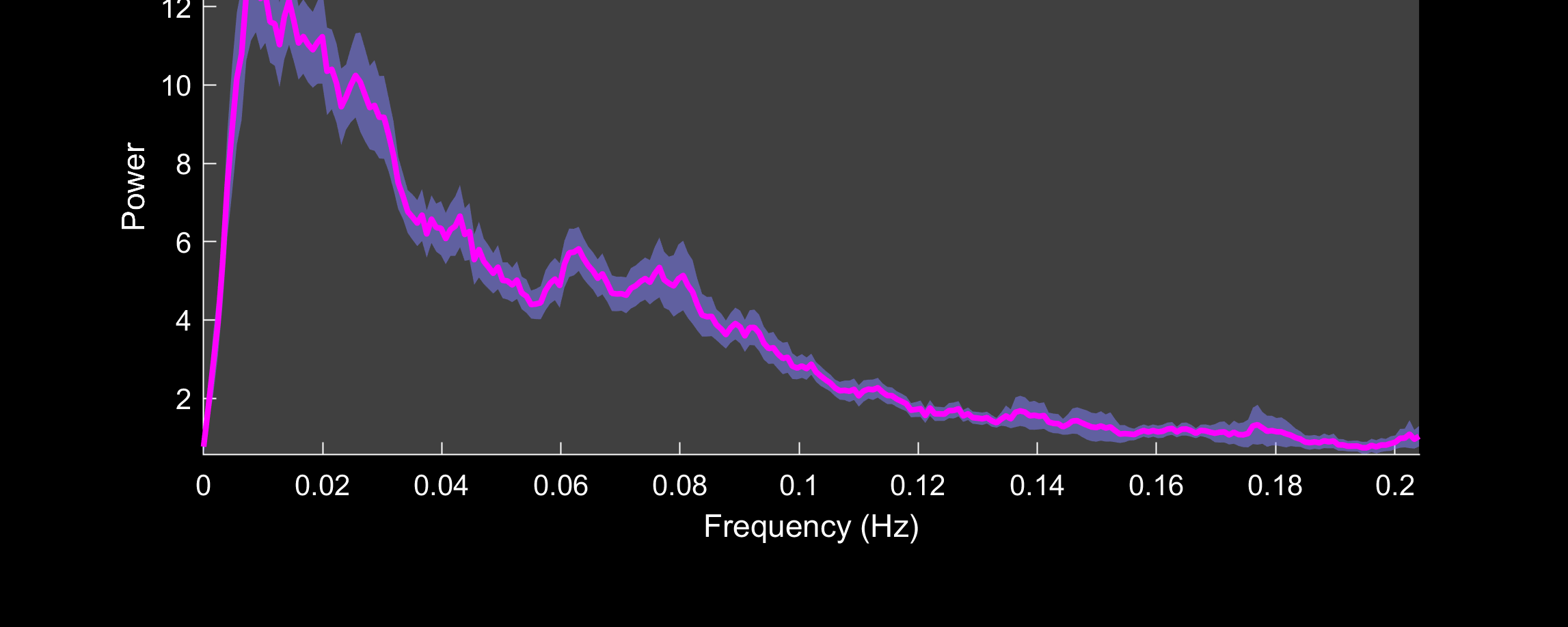

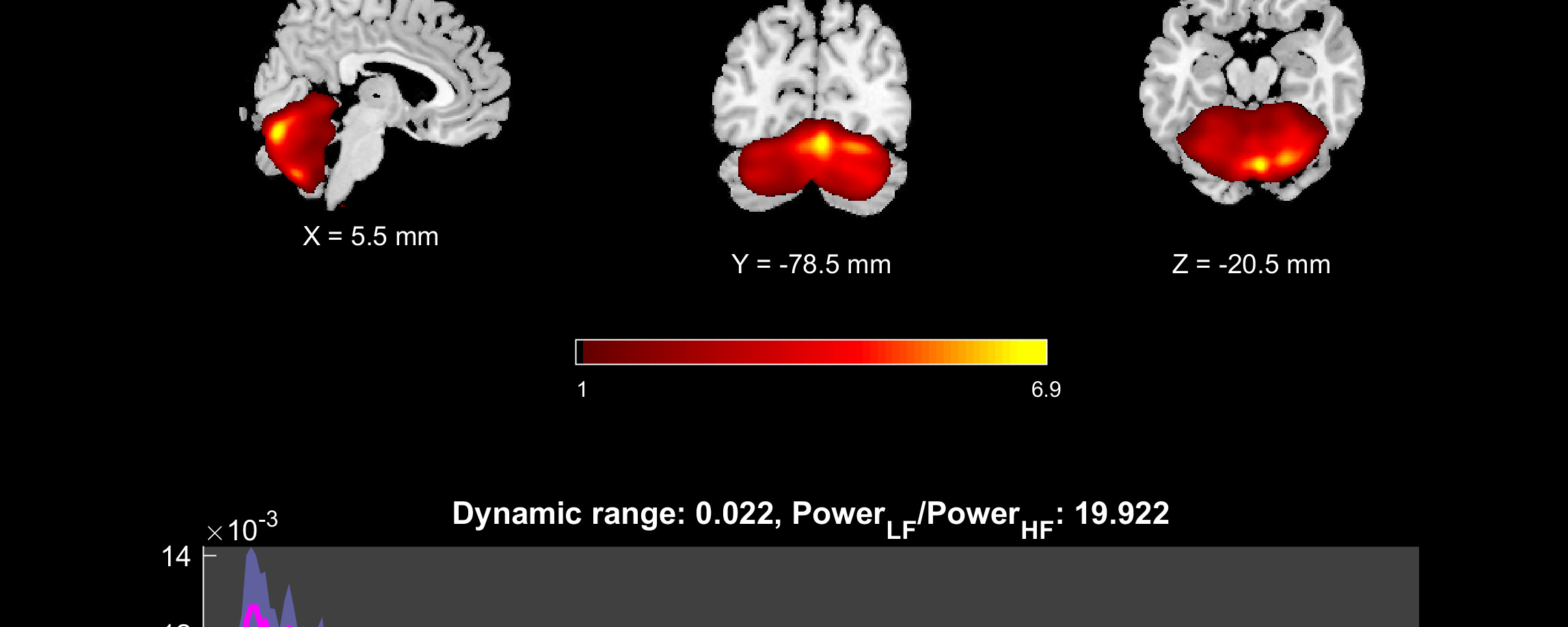

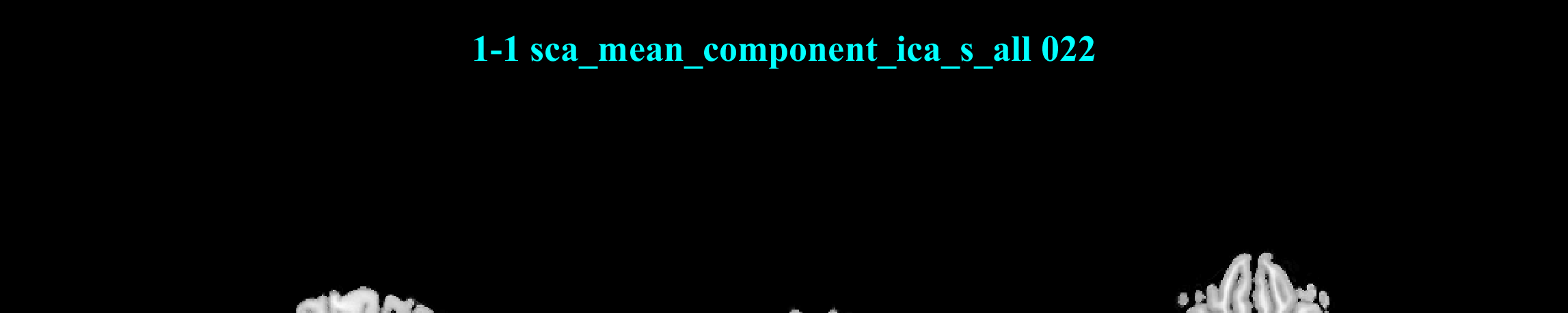

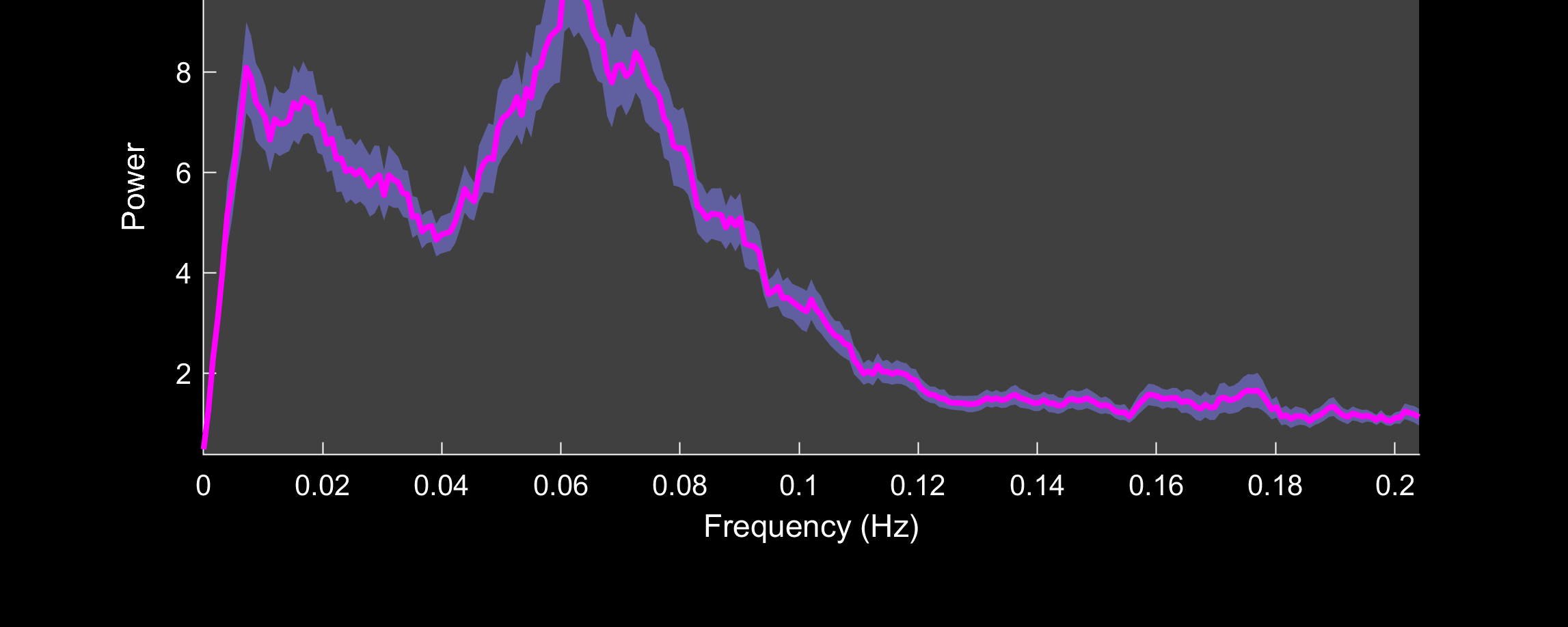

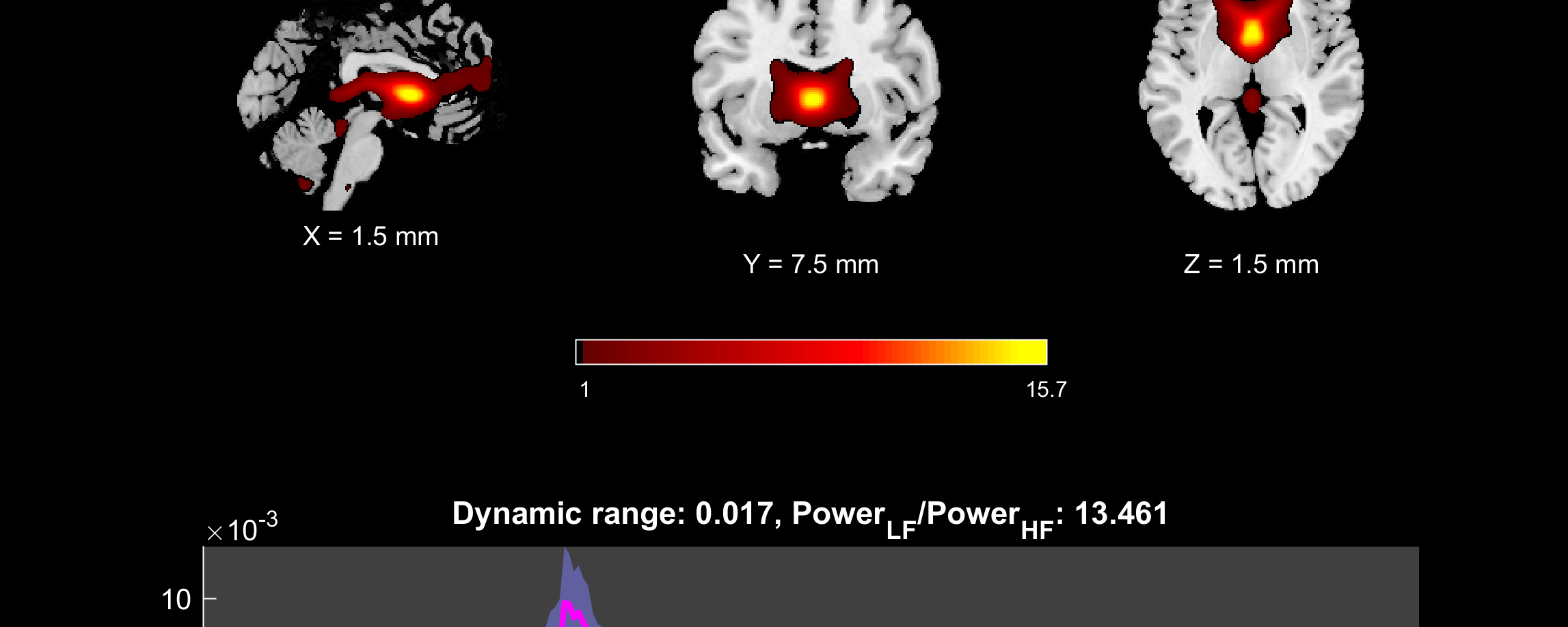

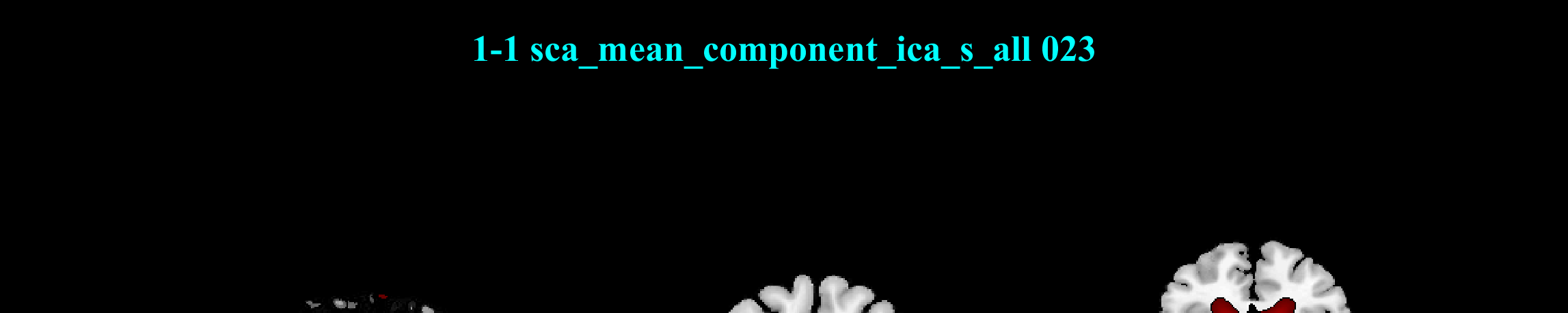

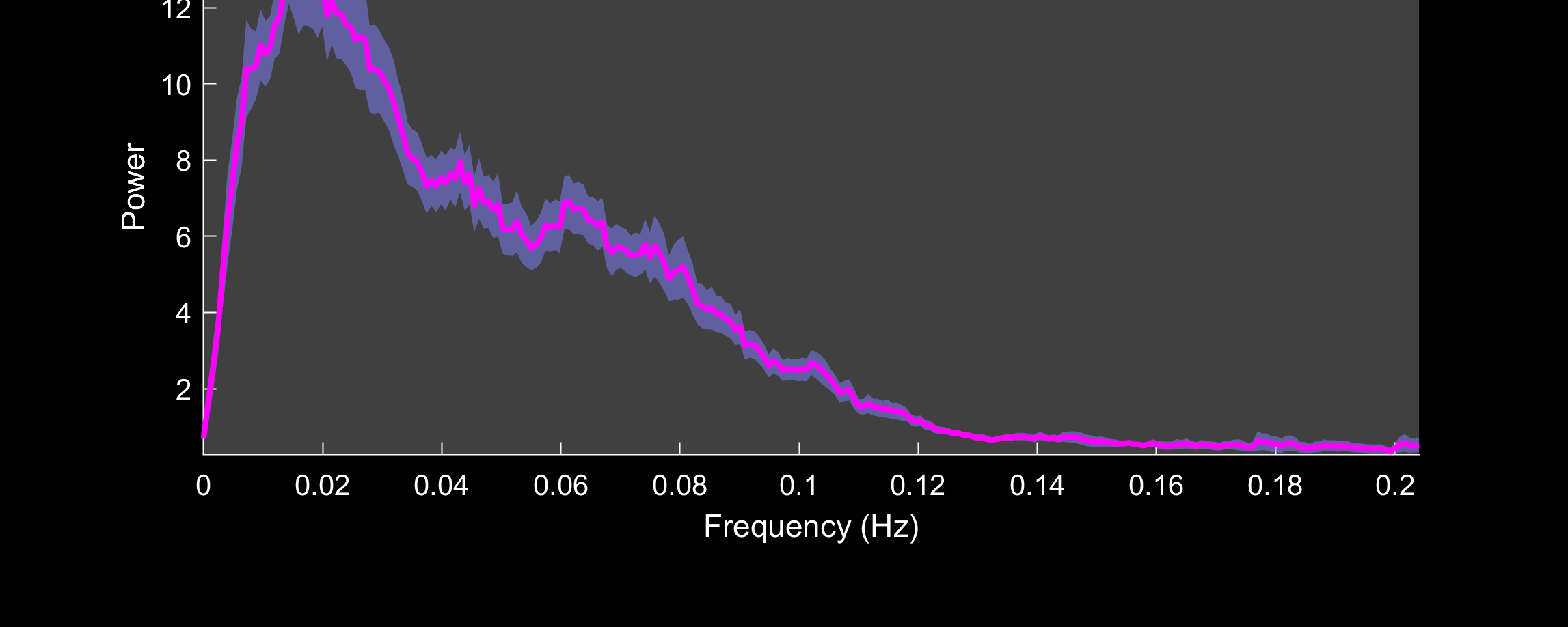

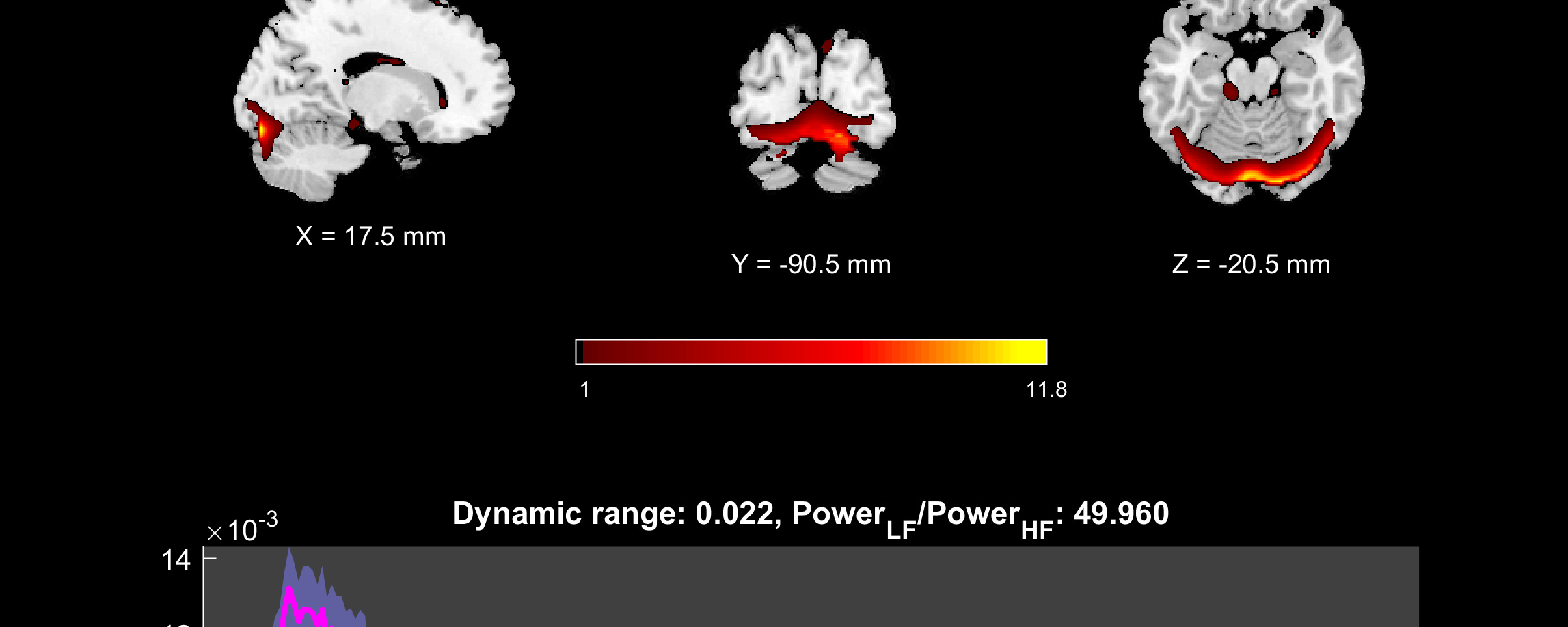

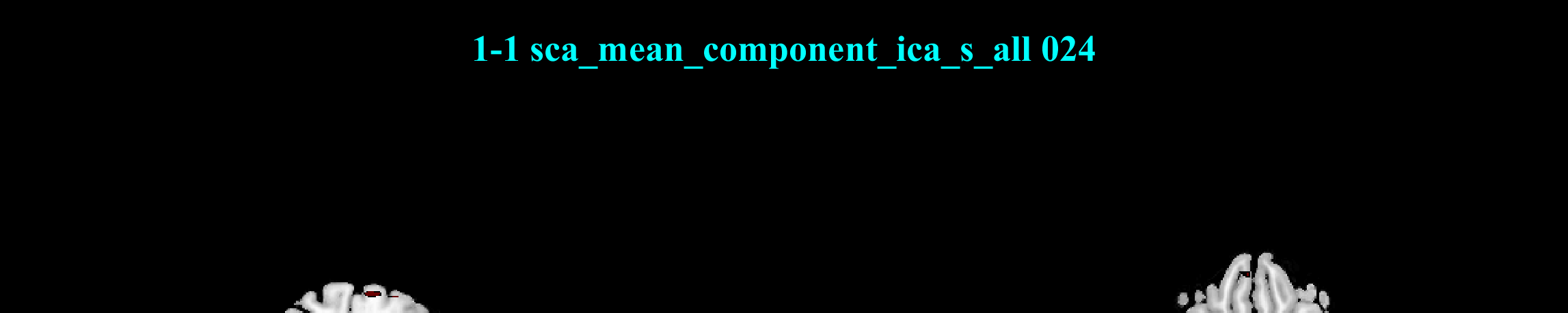

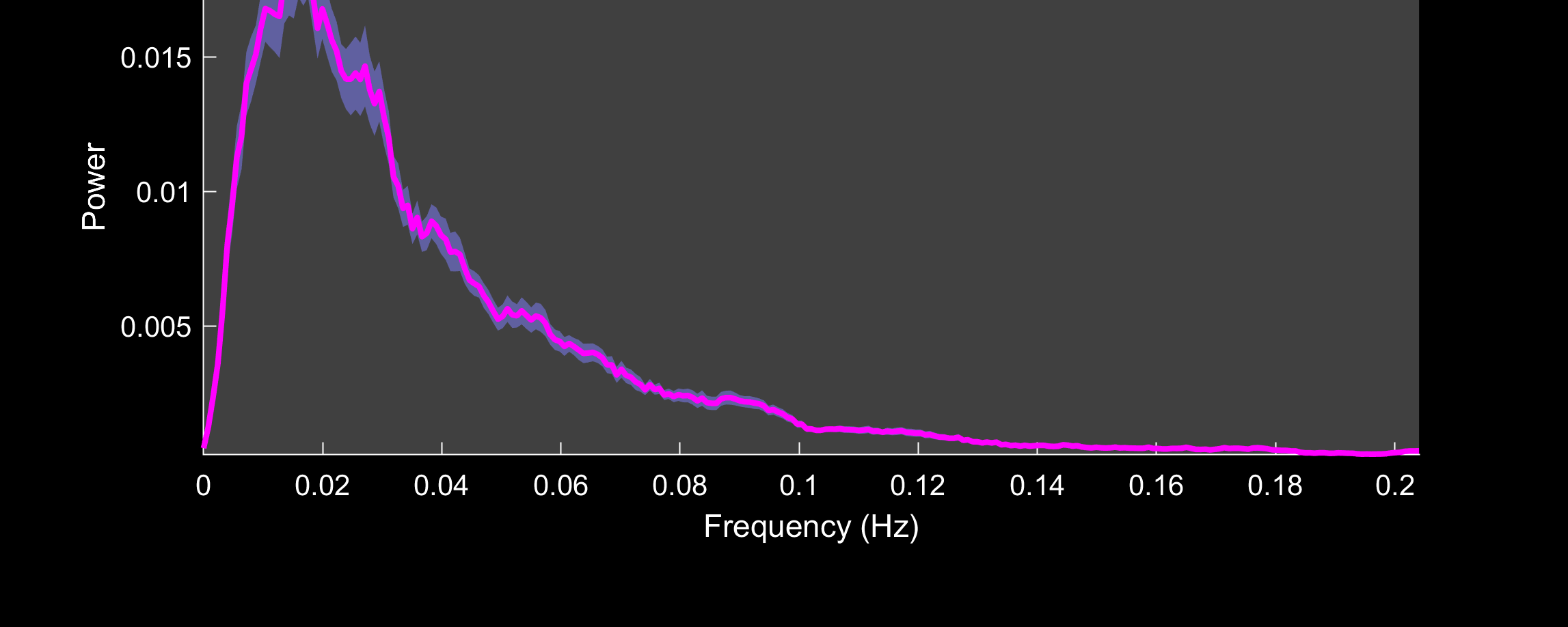

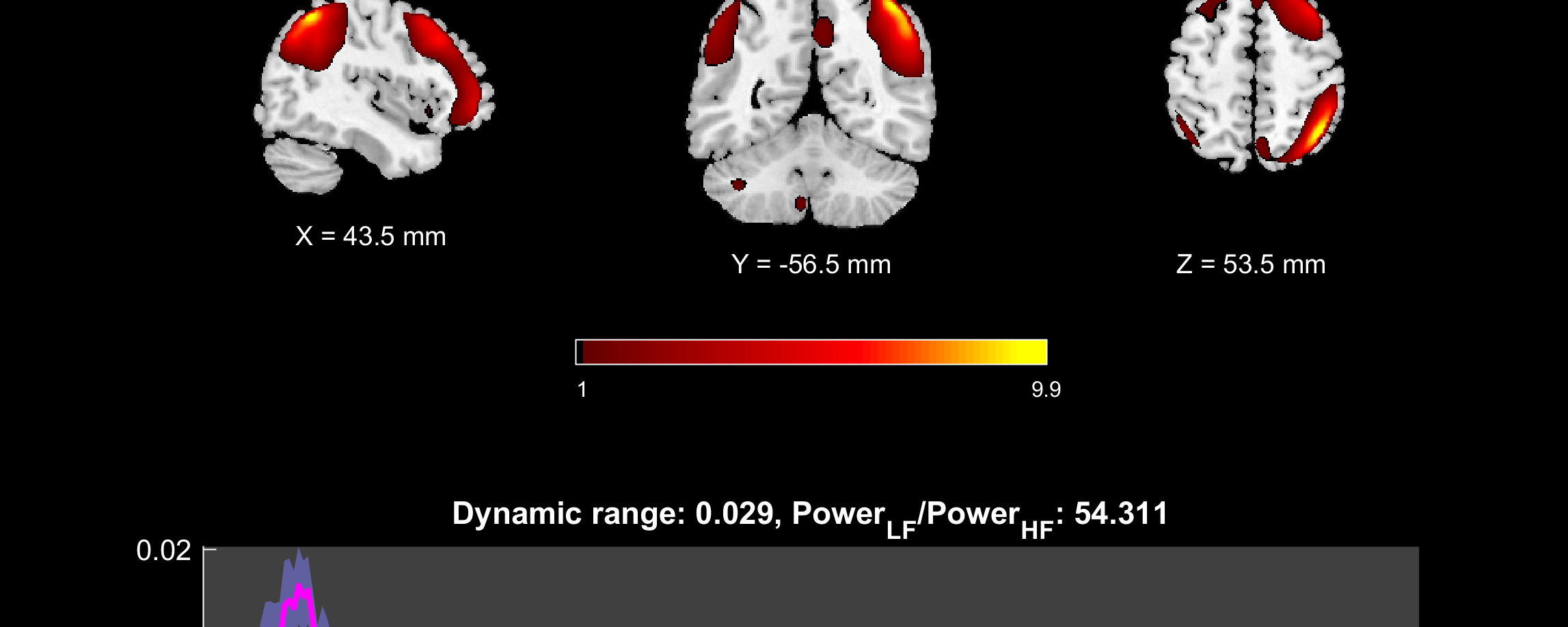

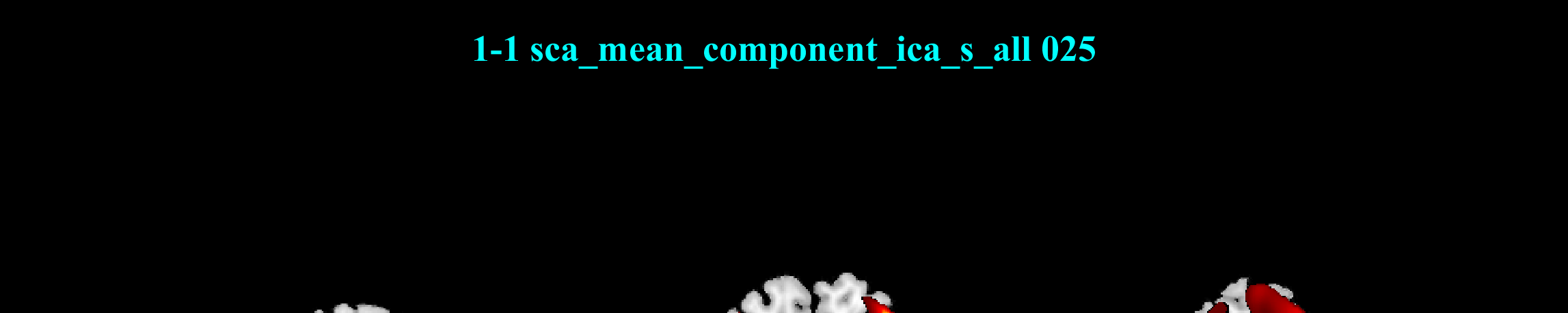

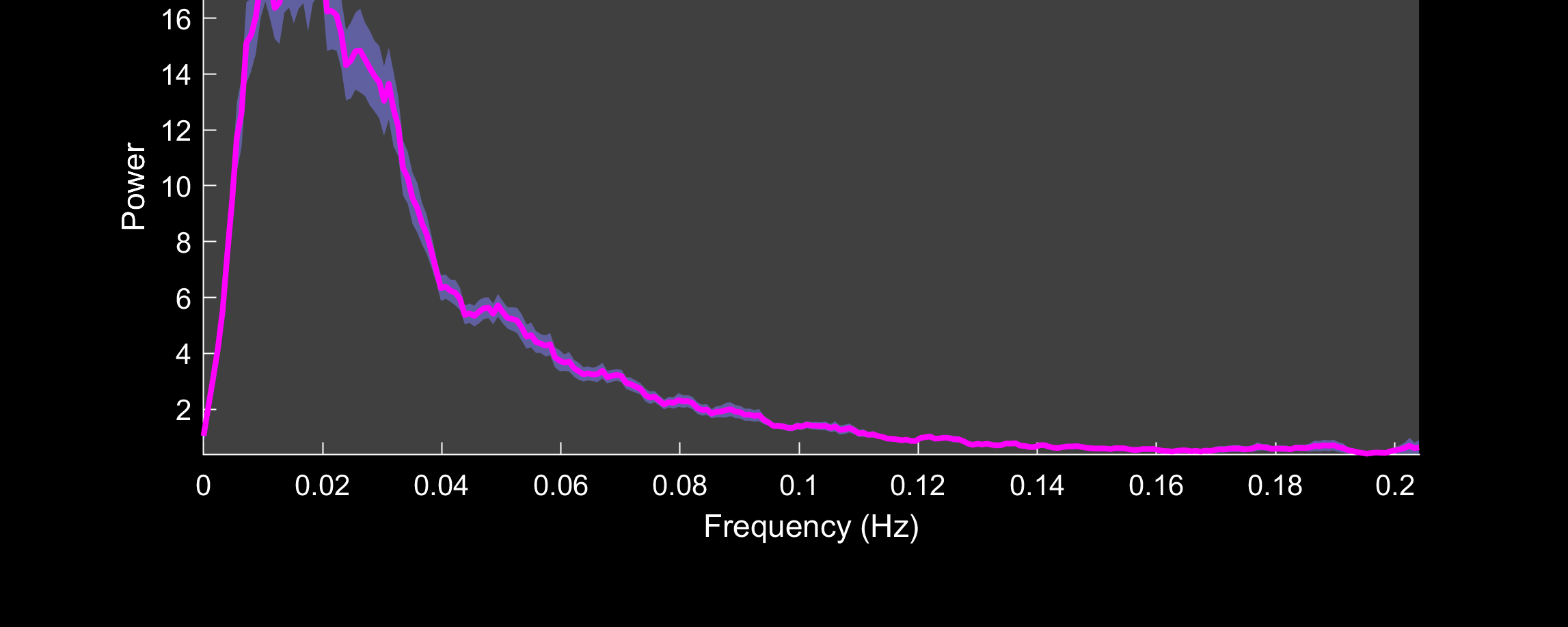

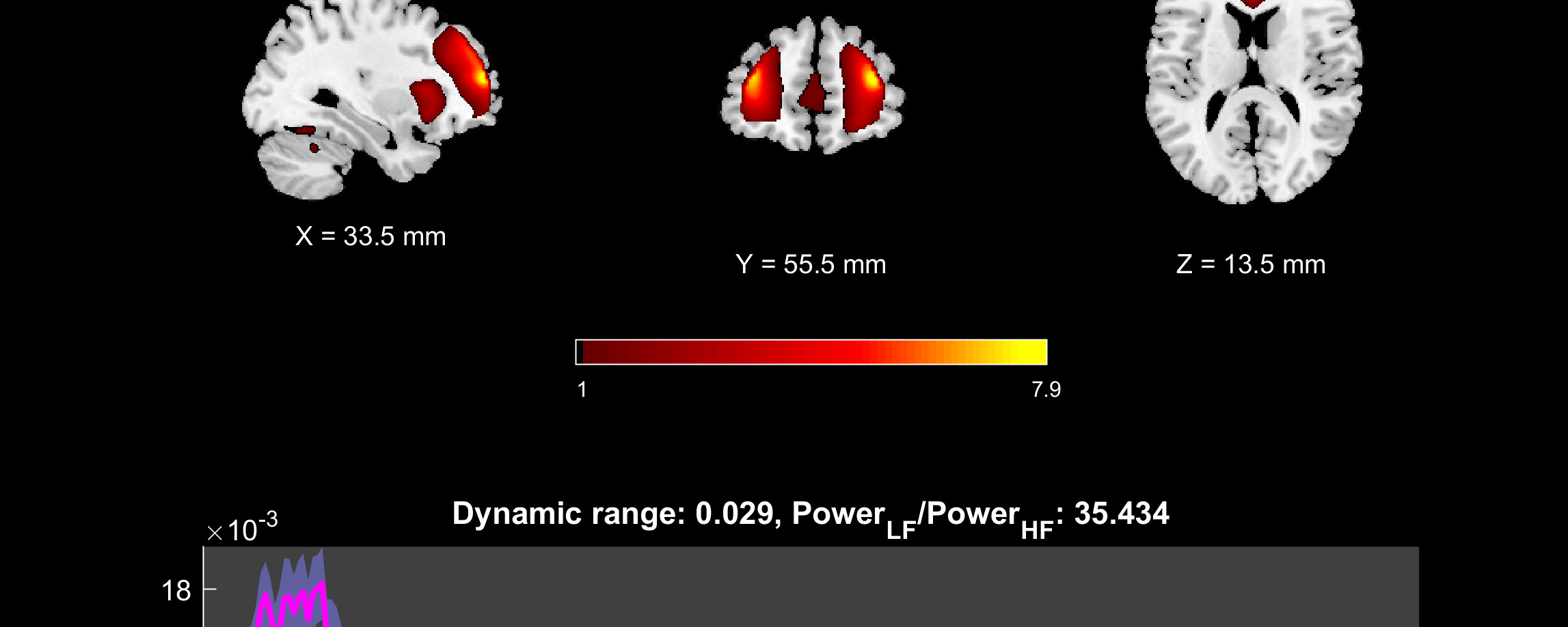

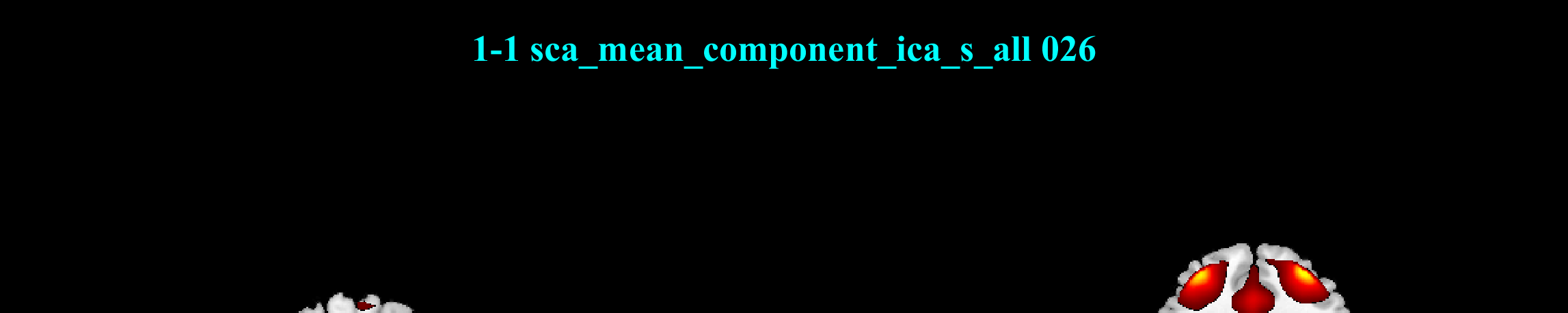

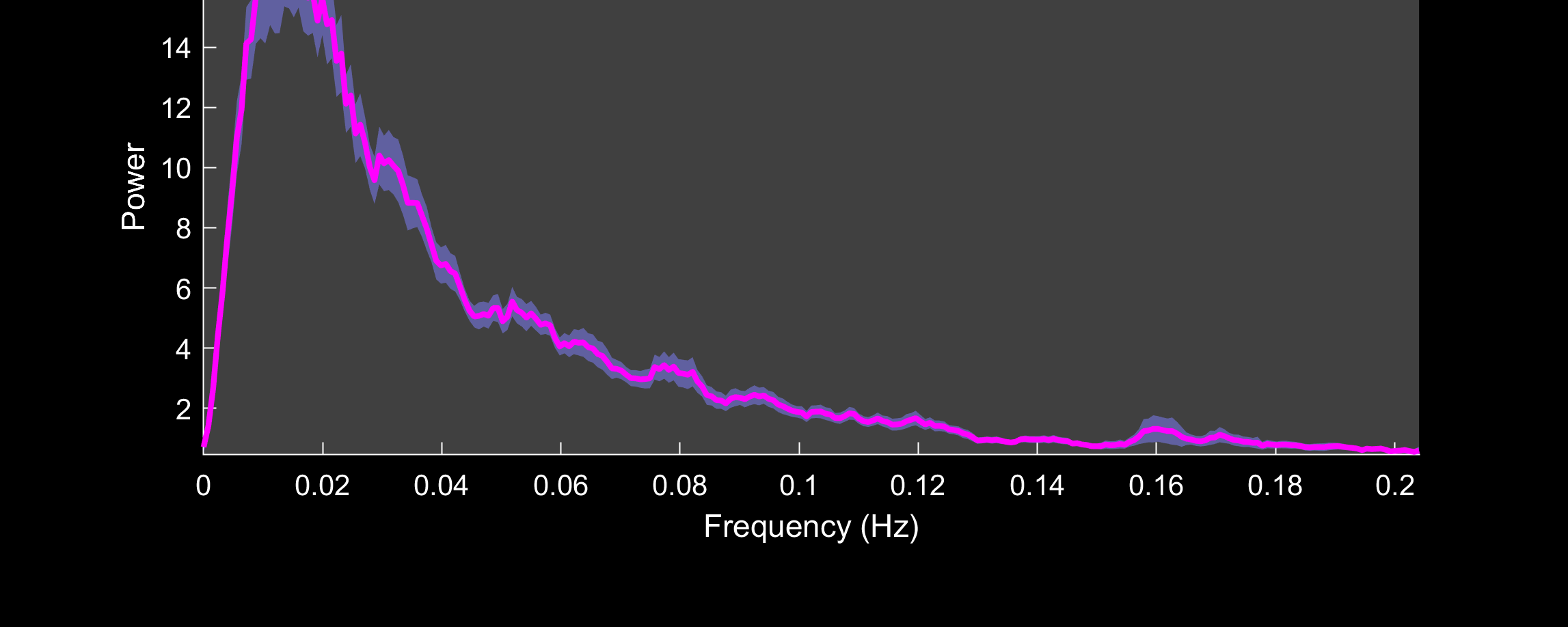

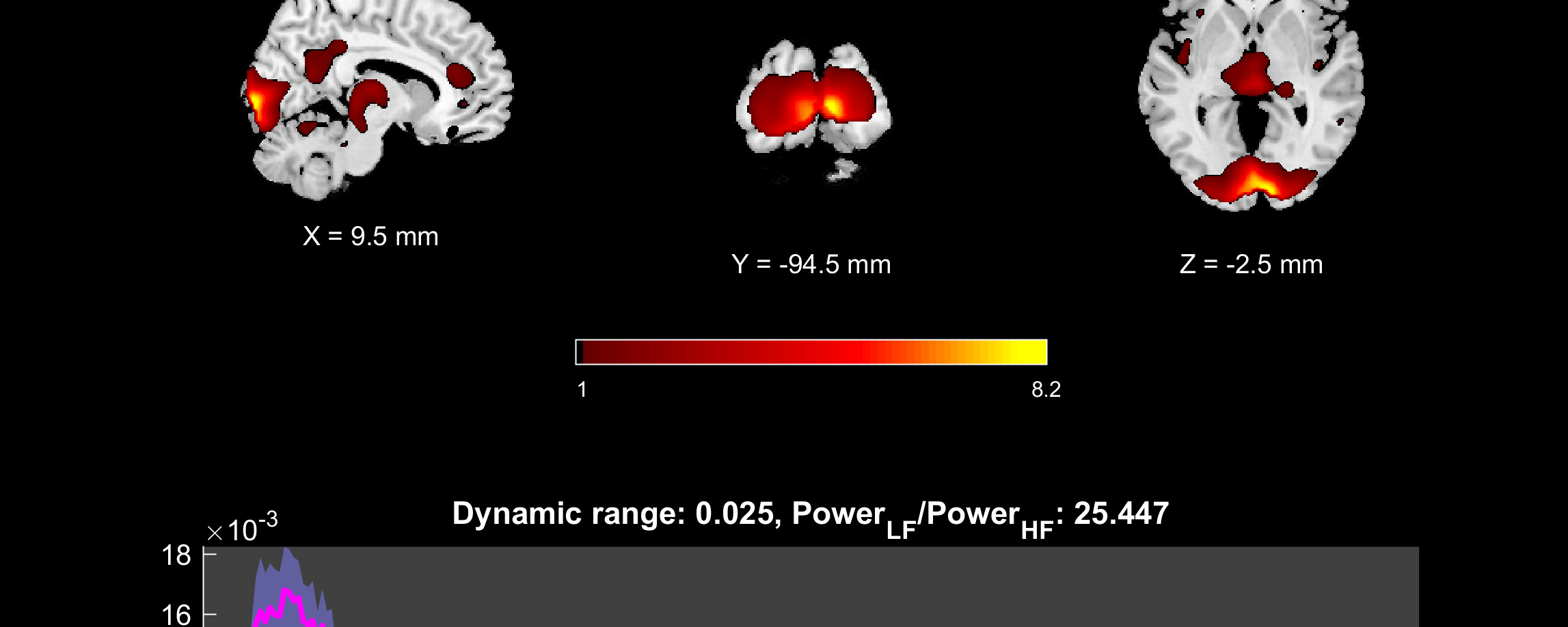

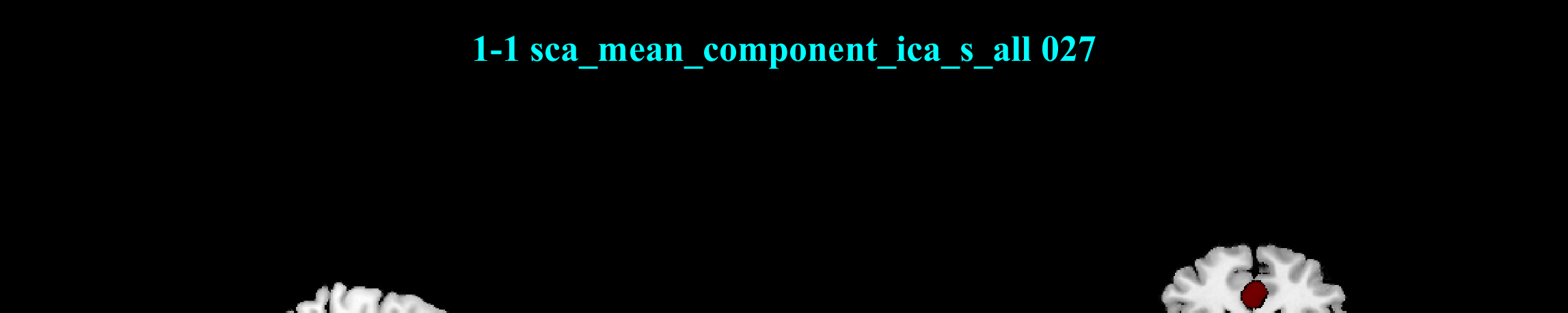

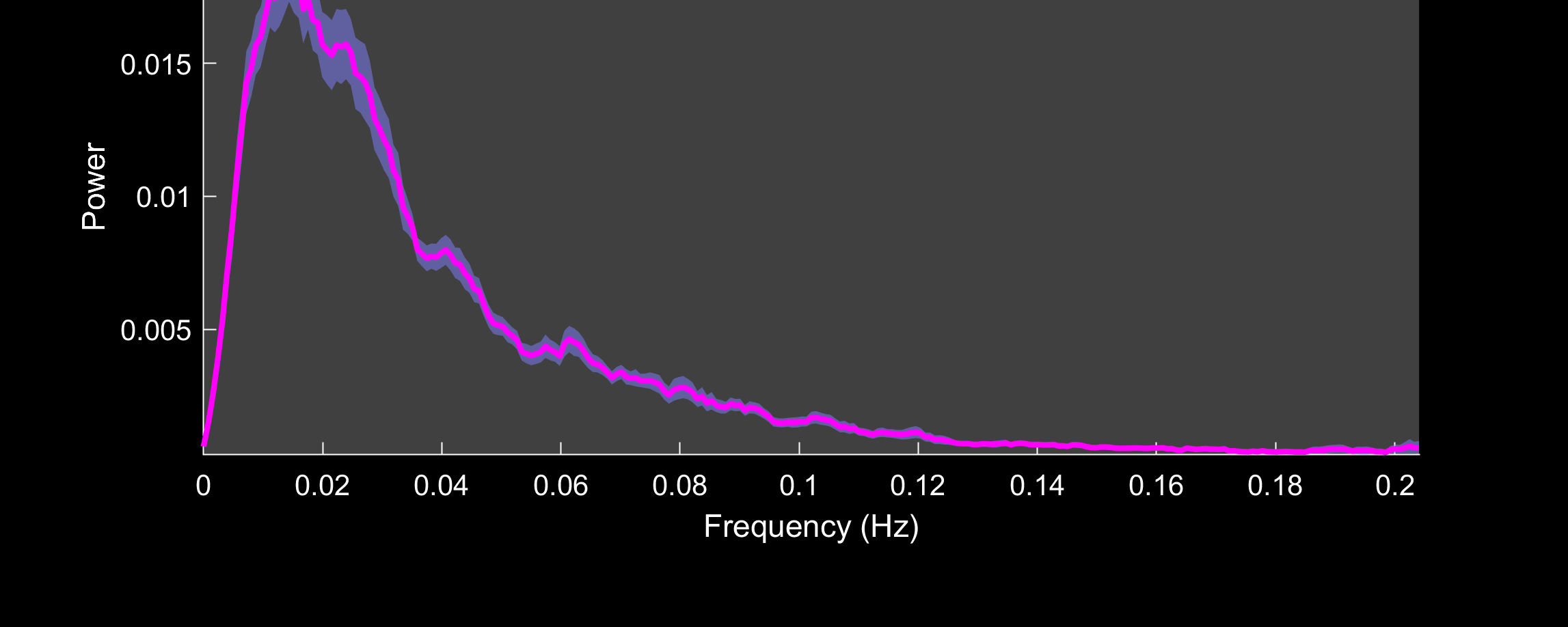

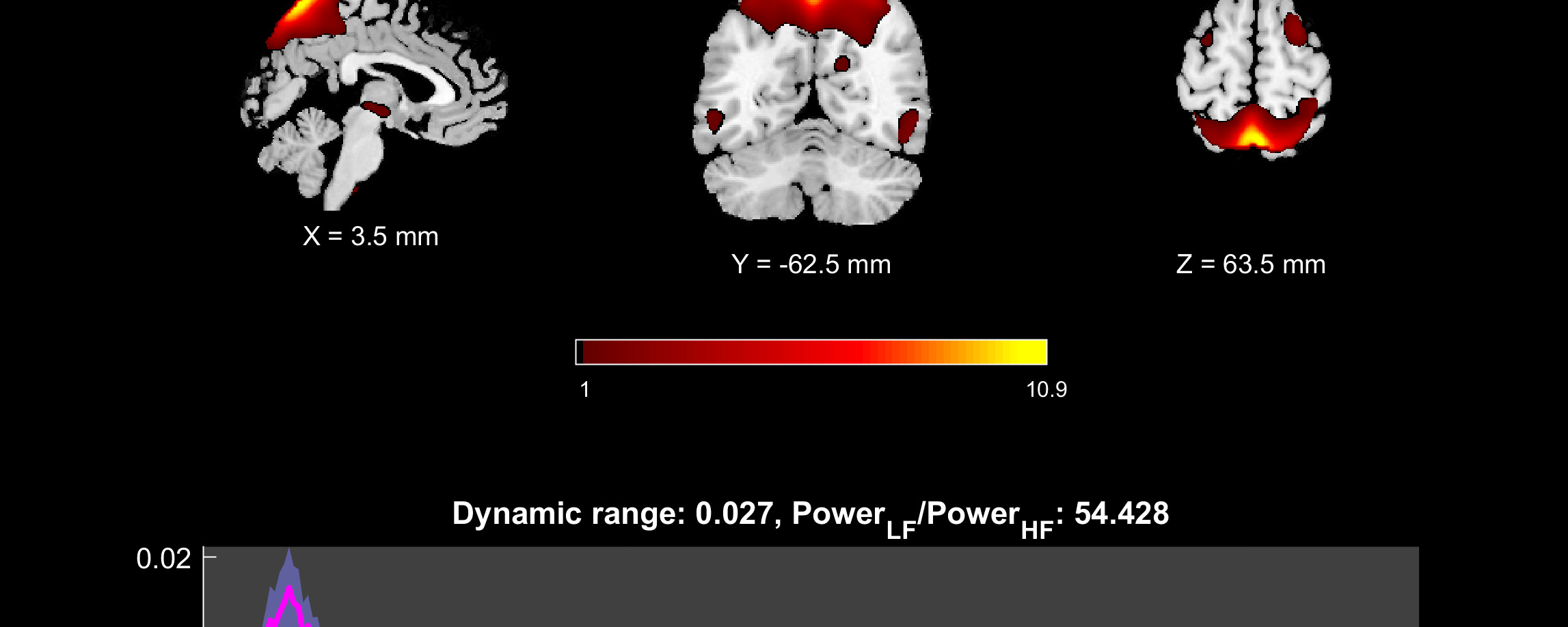

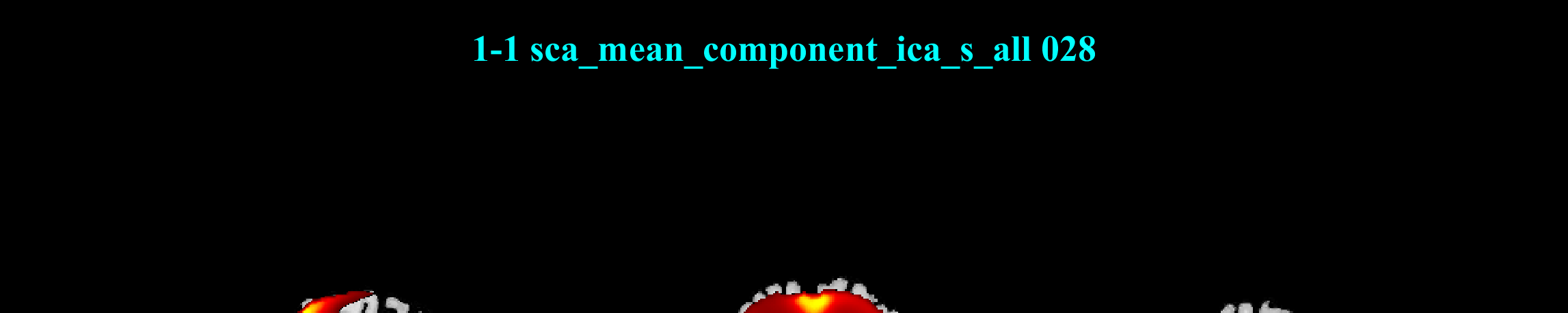

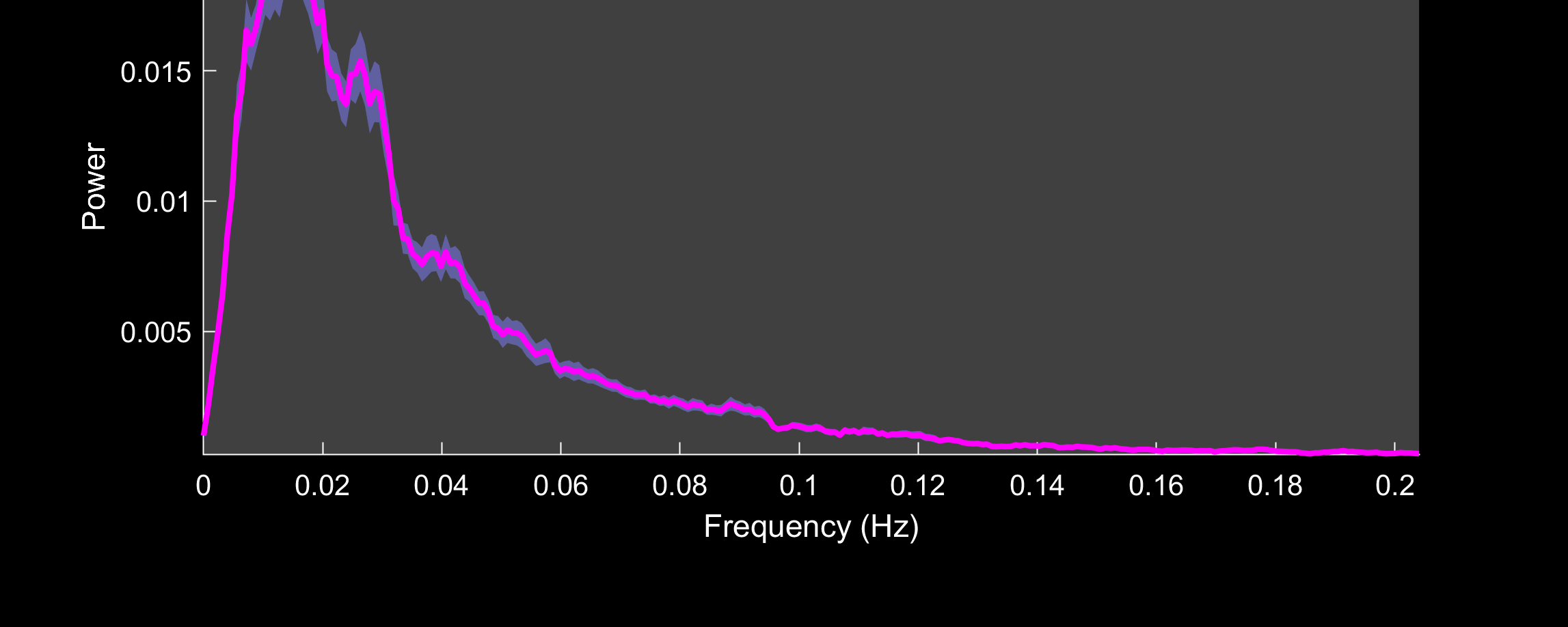

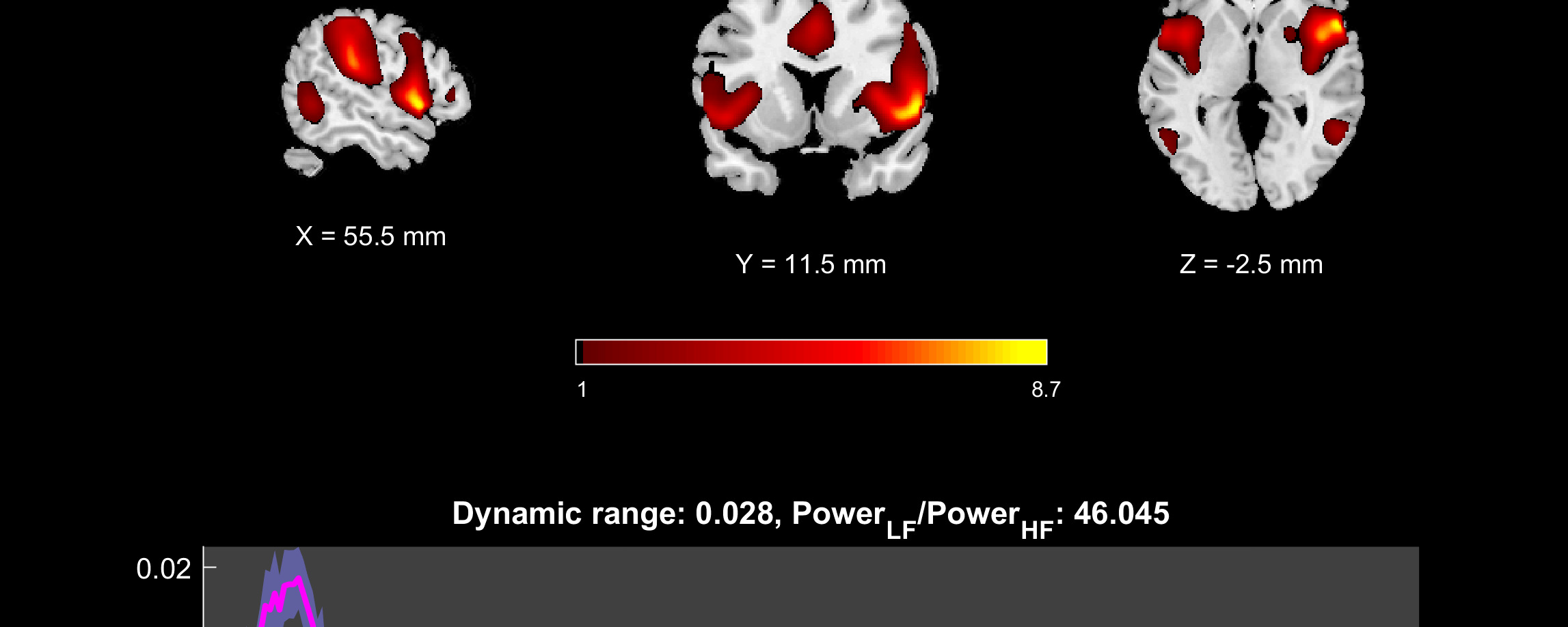

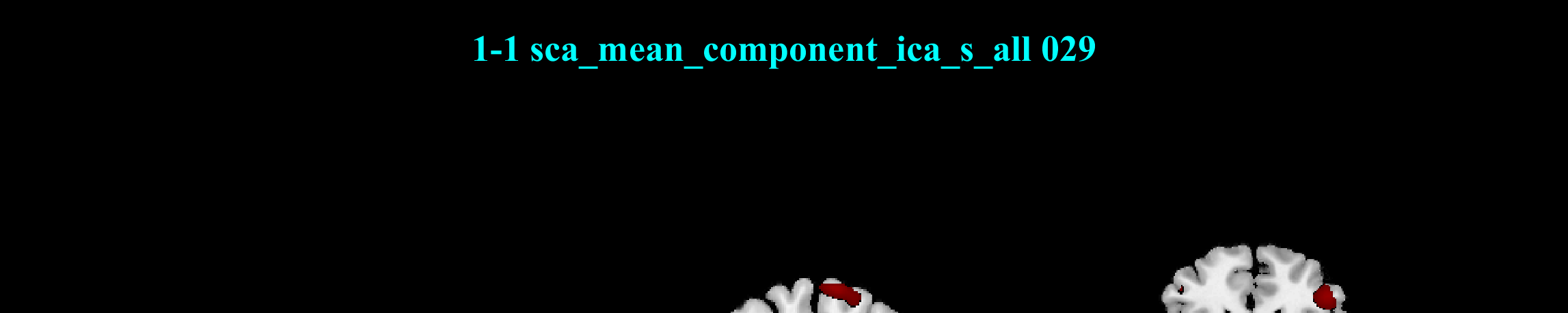

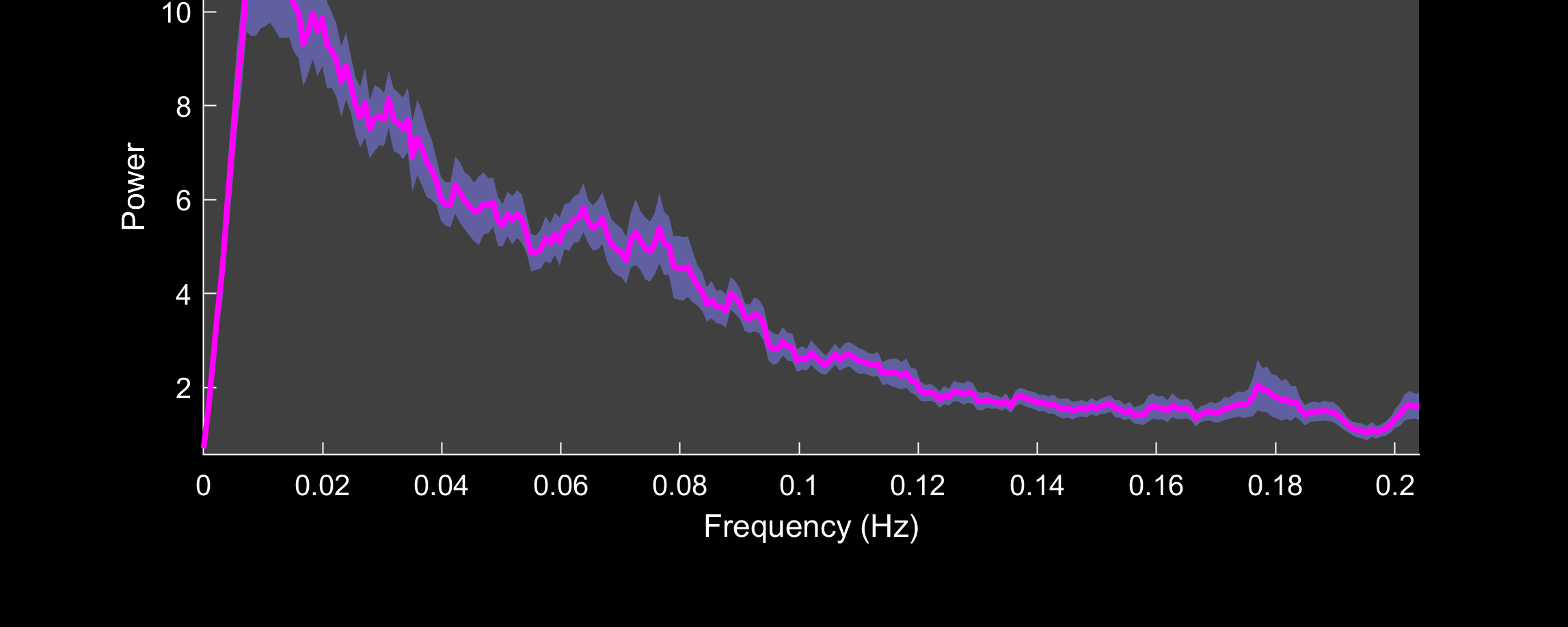

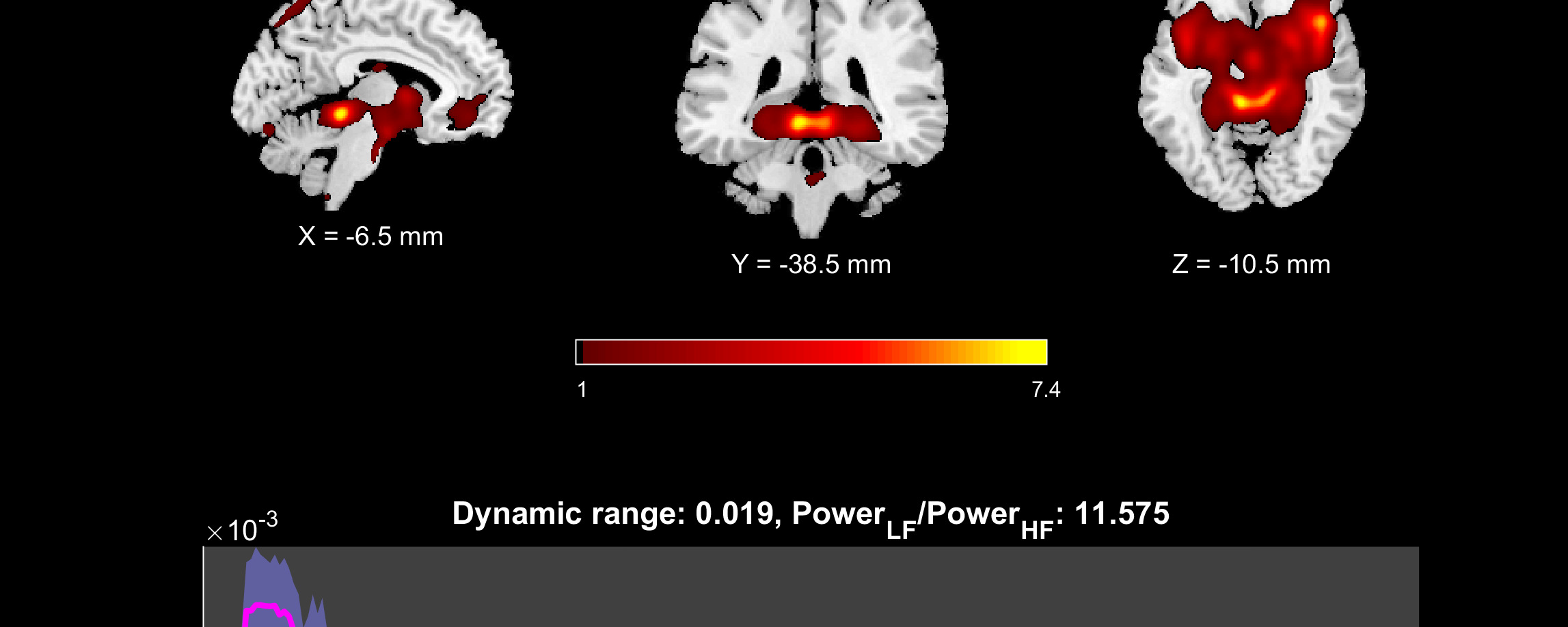

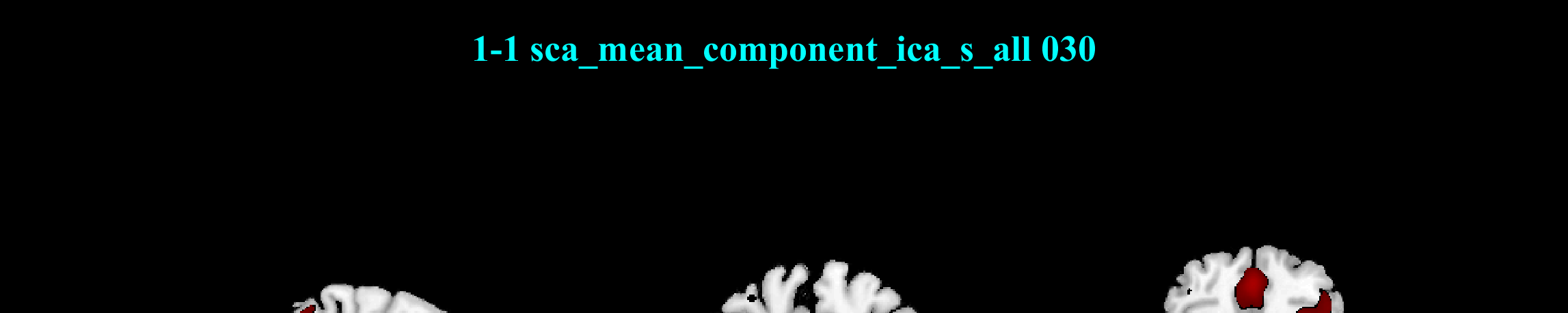

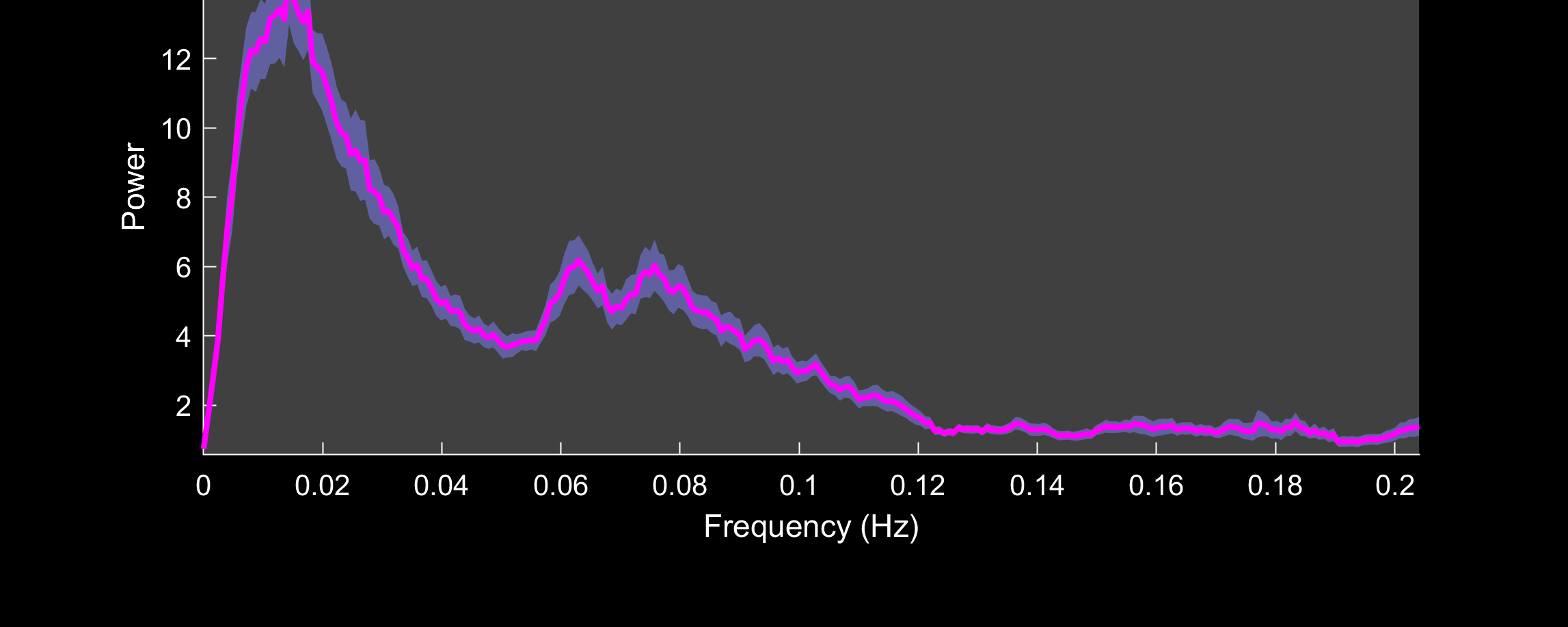

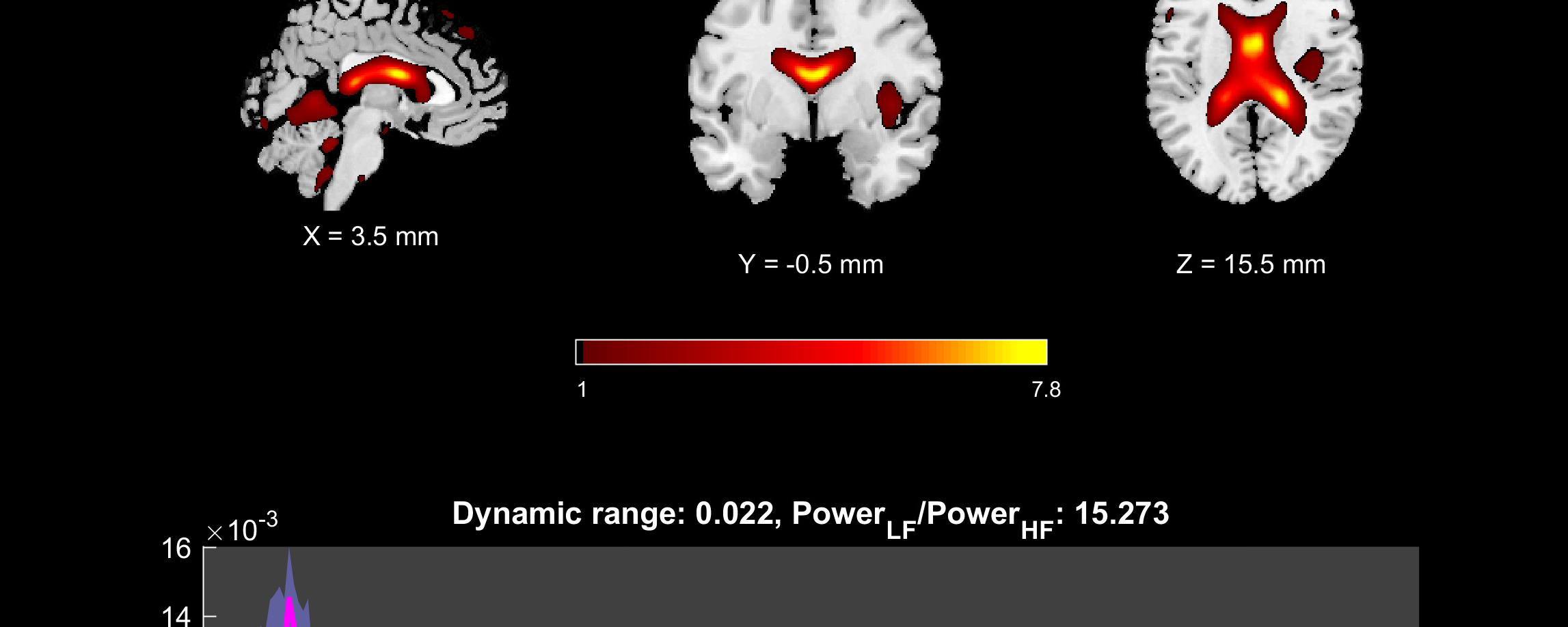

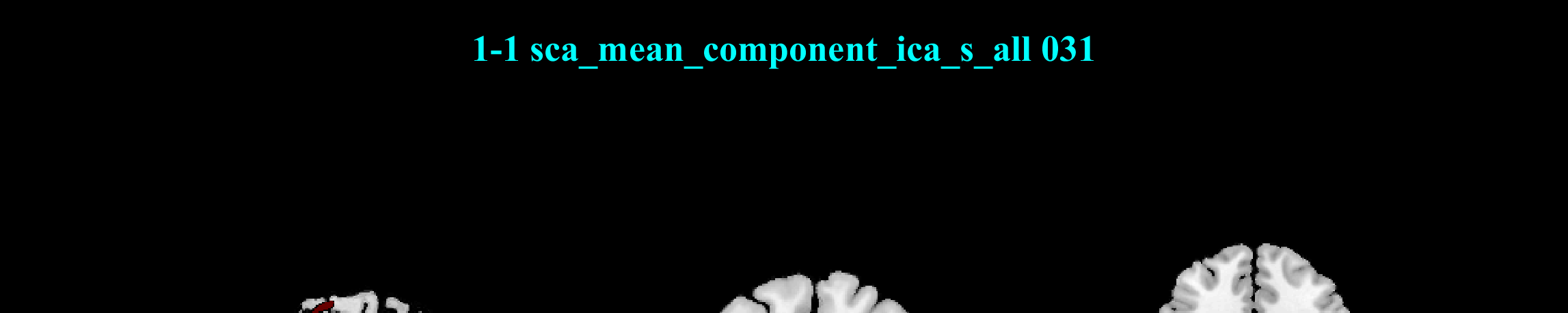

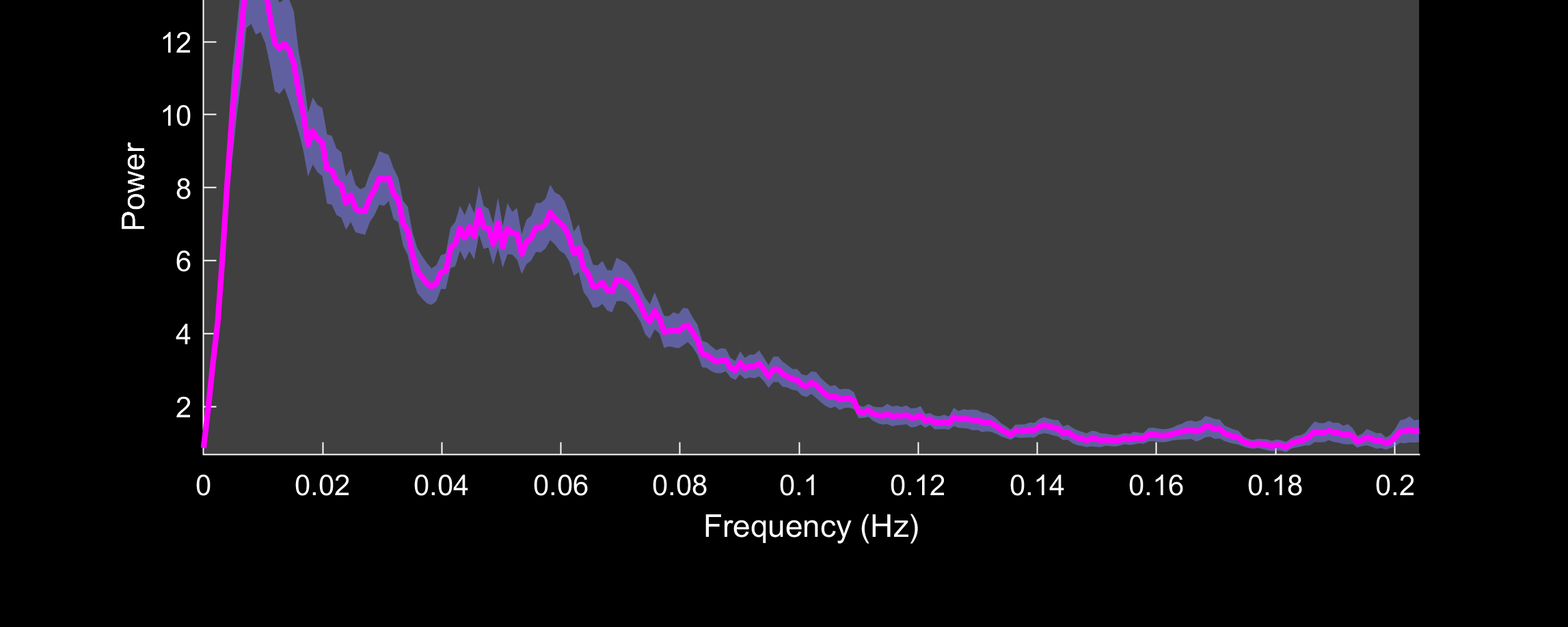

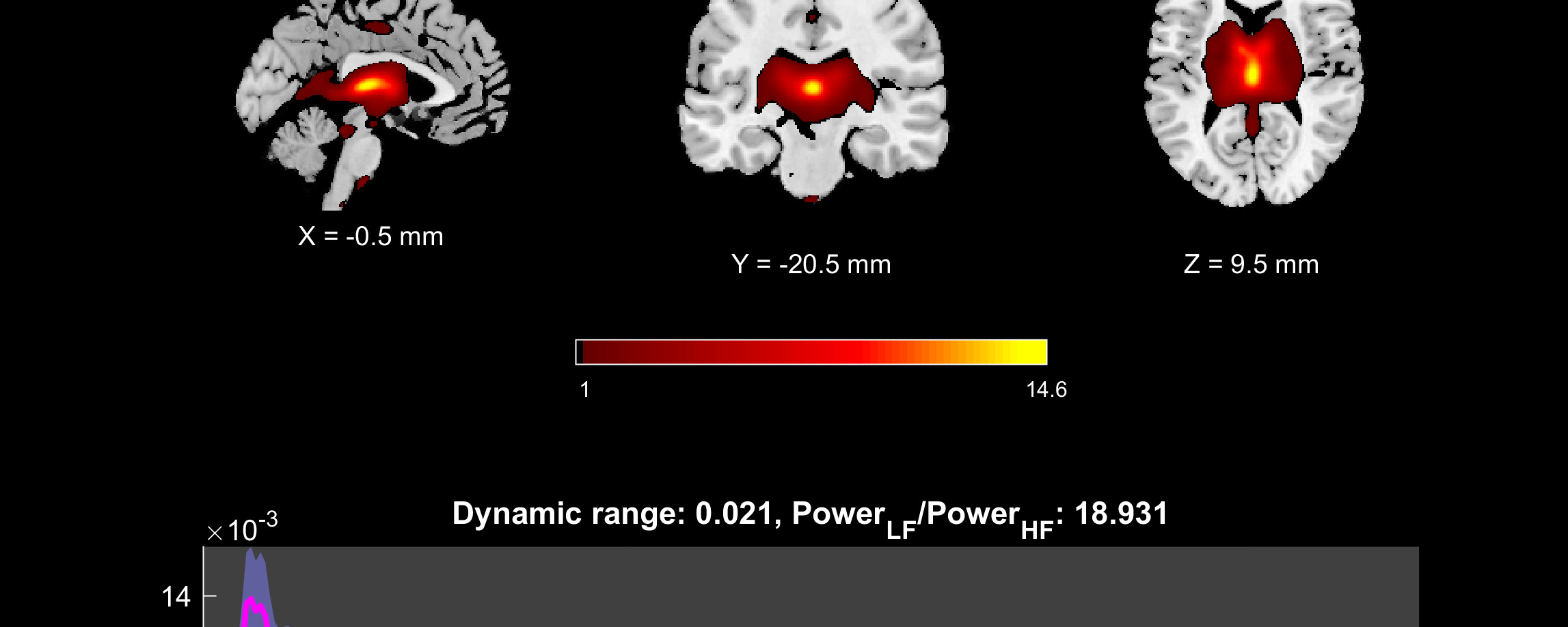

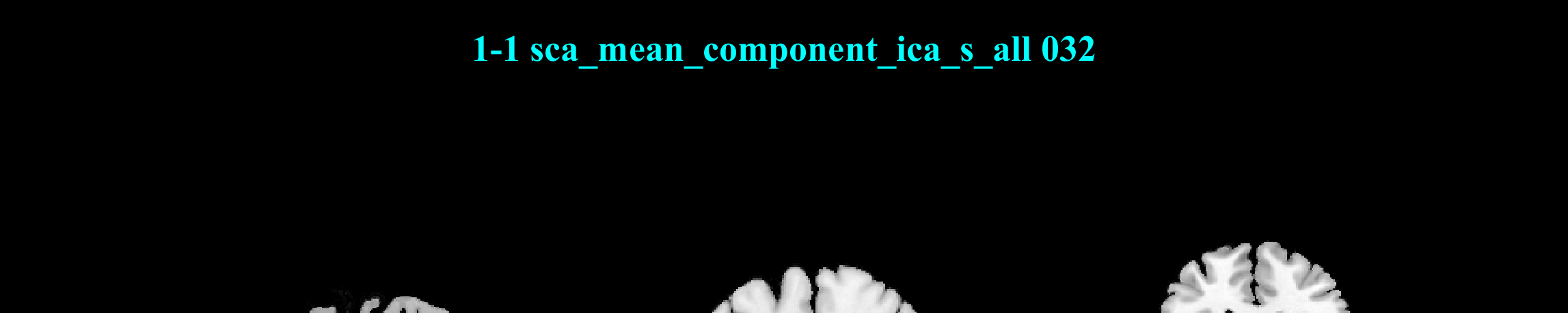

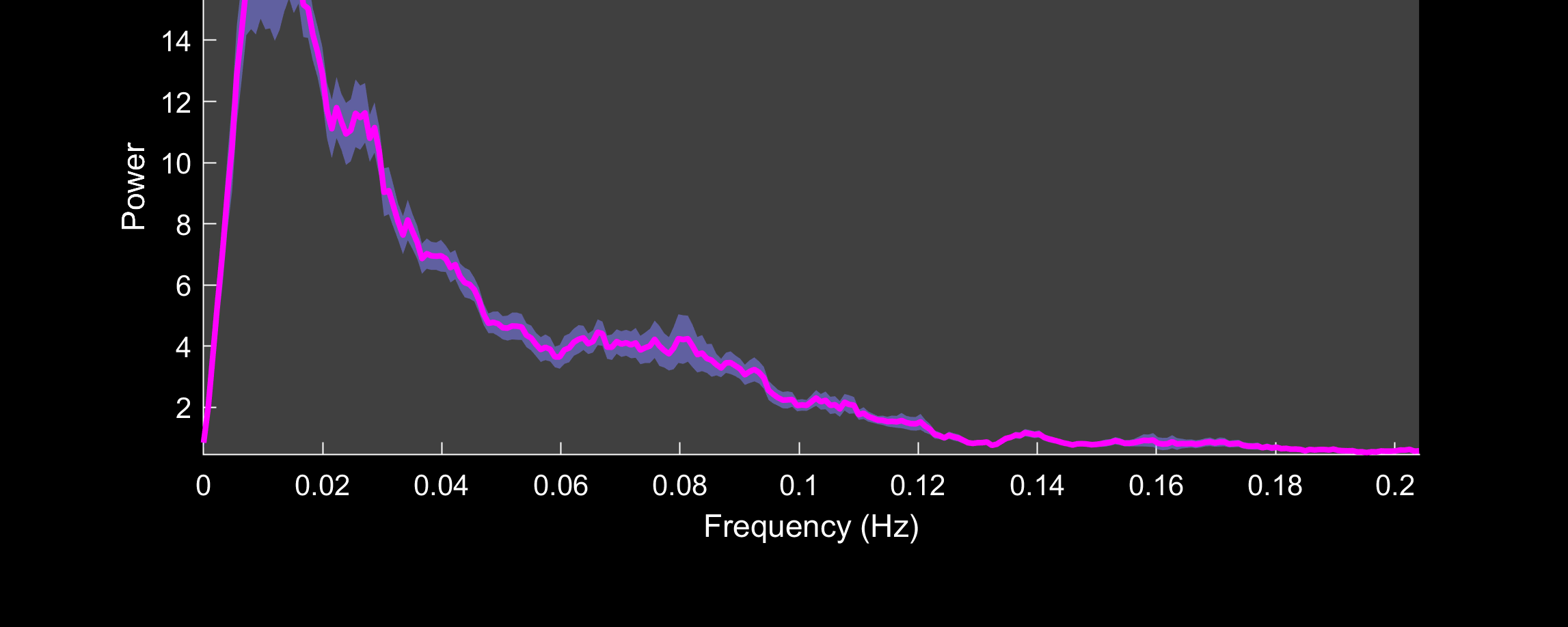

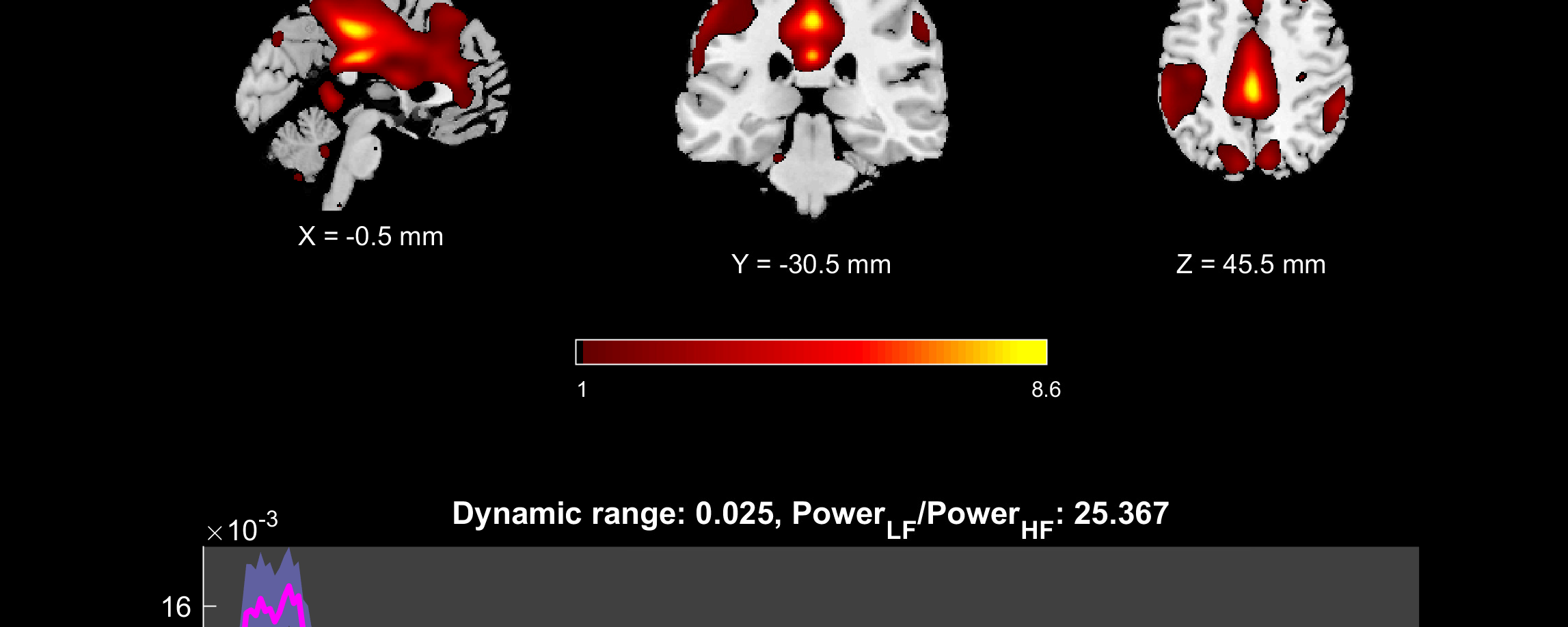

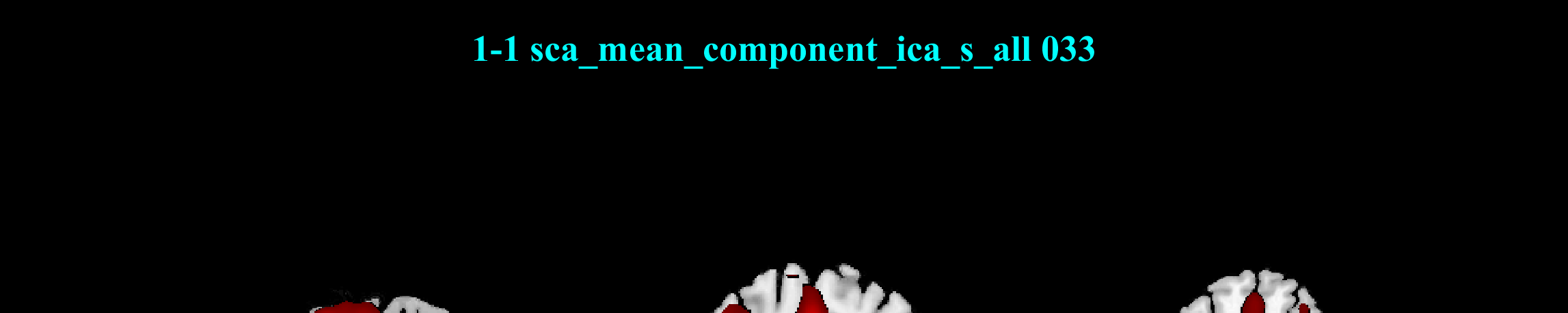

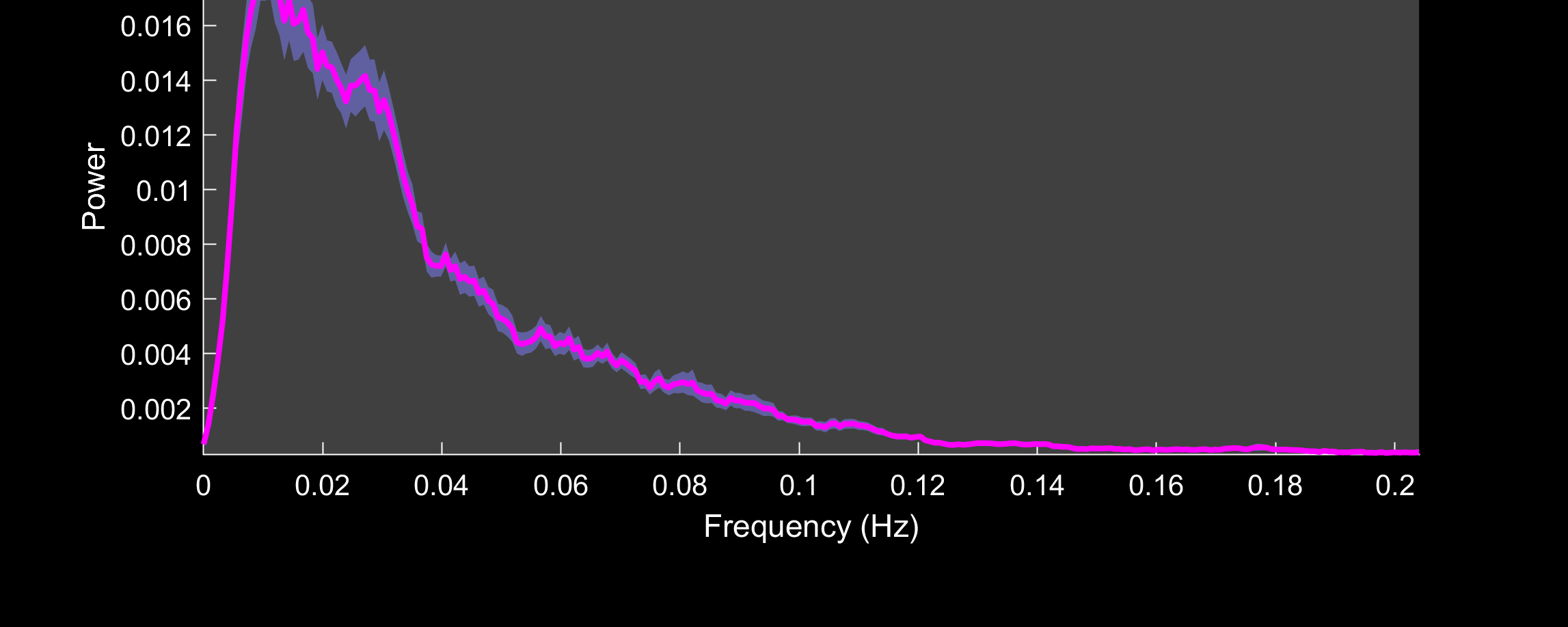

Supplement: Supplementary data 1 [file mmc1.docx]

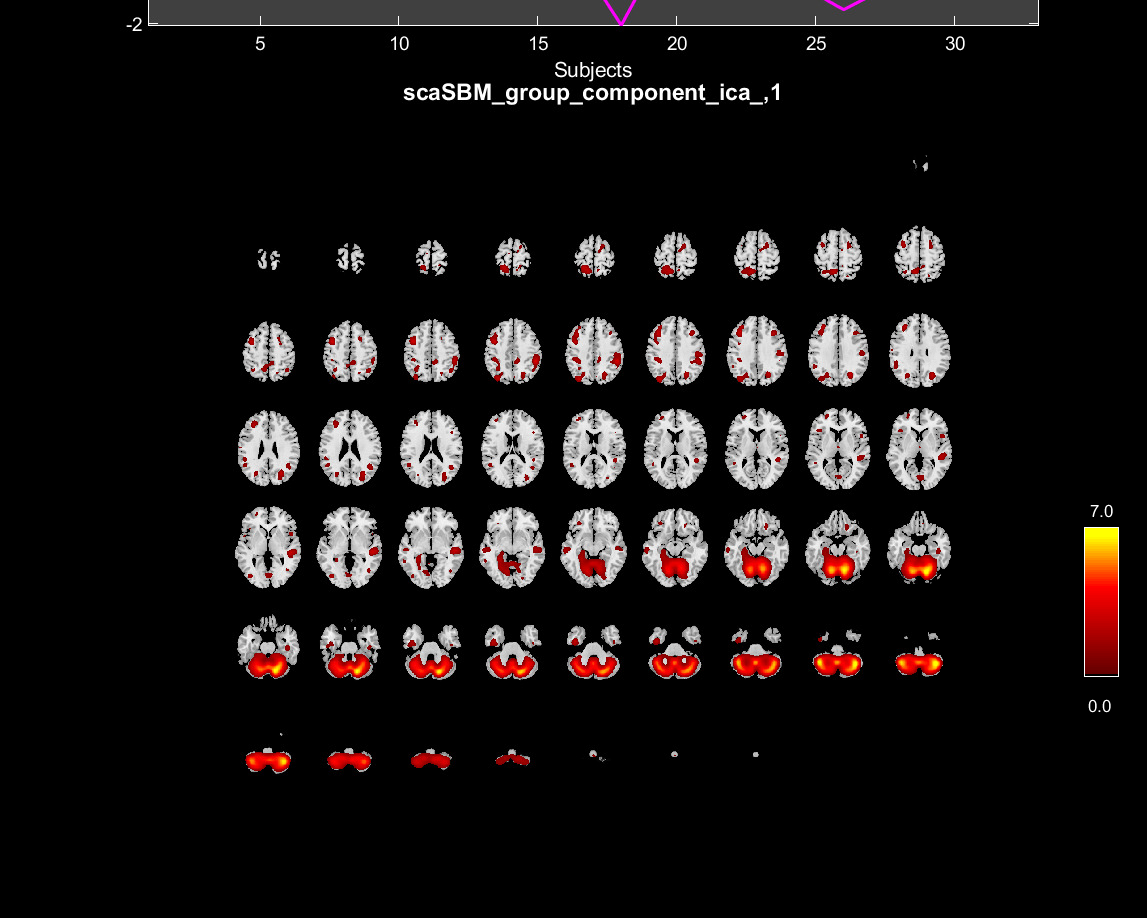

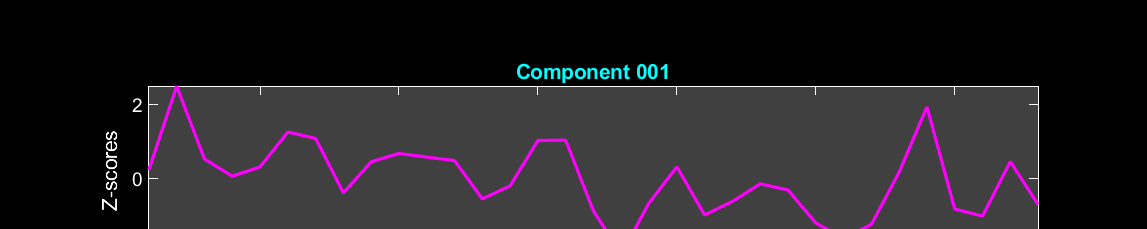

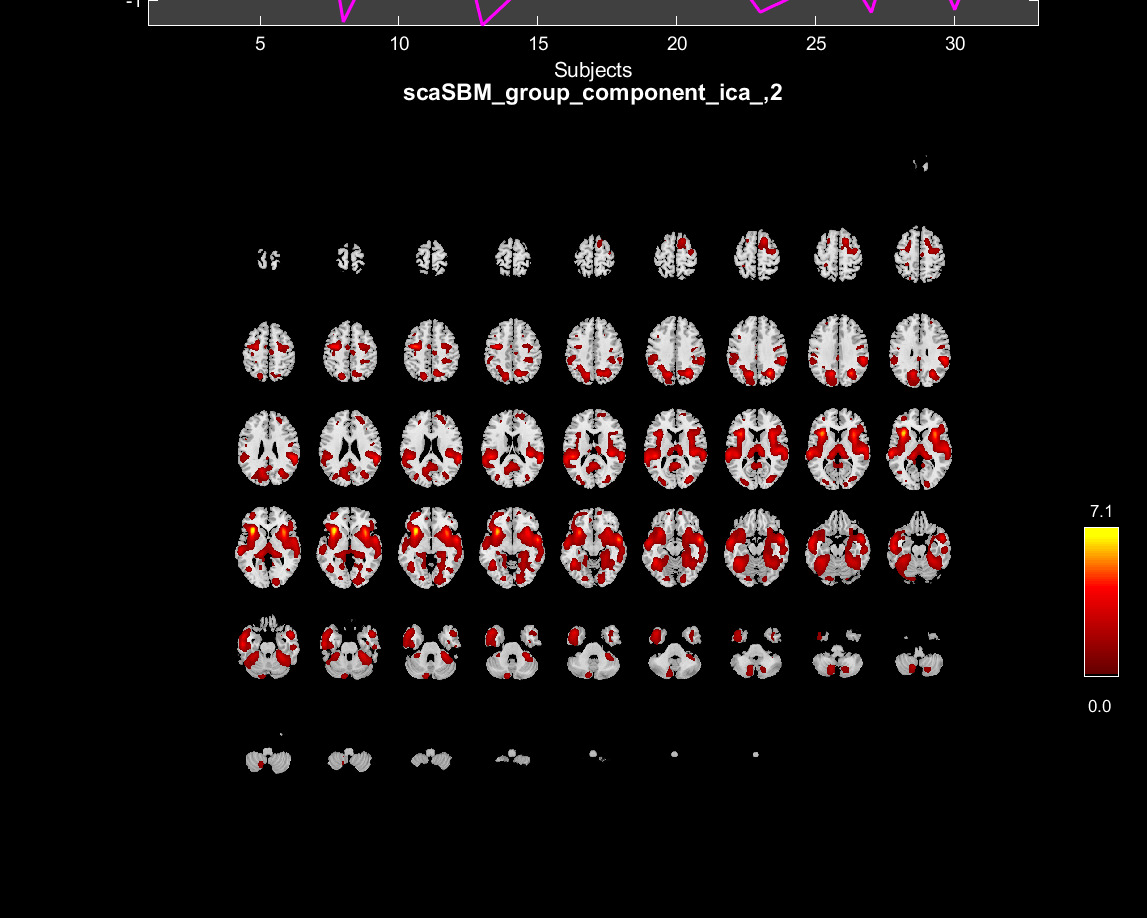

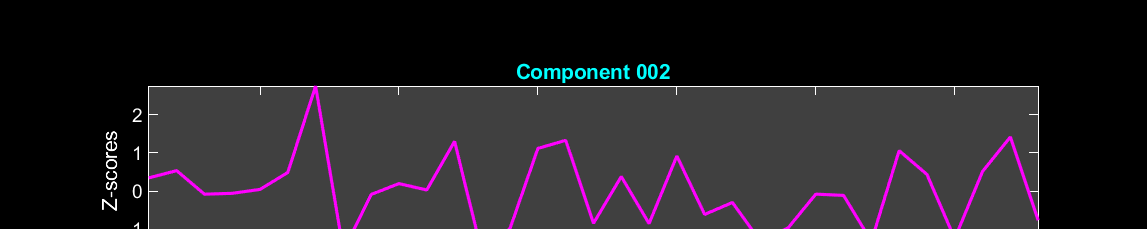

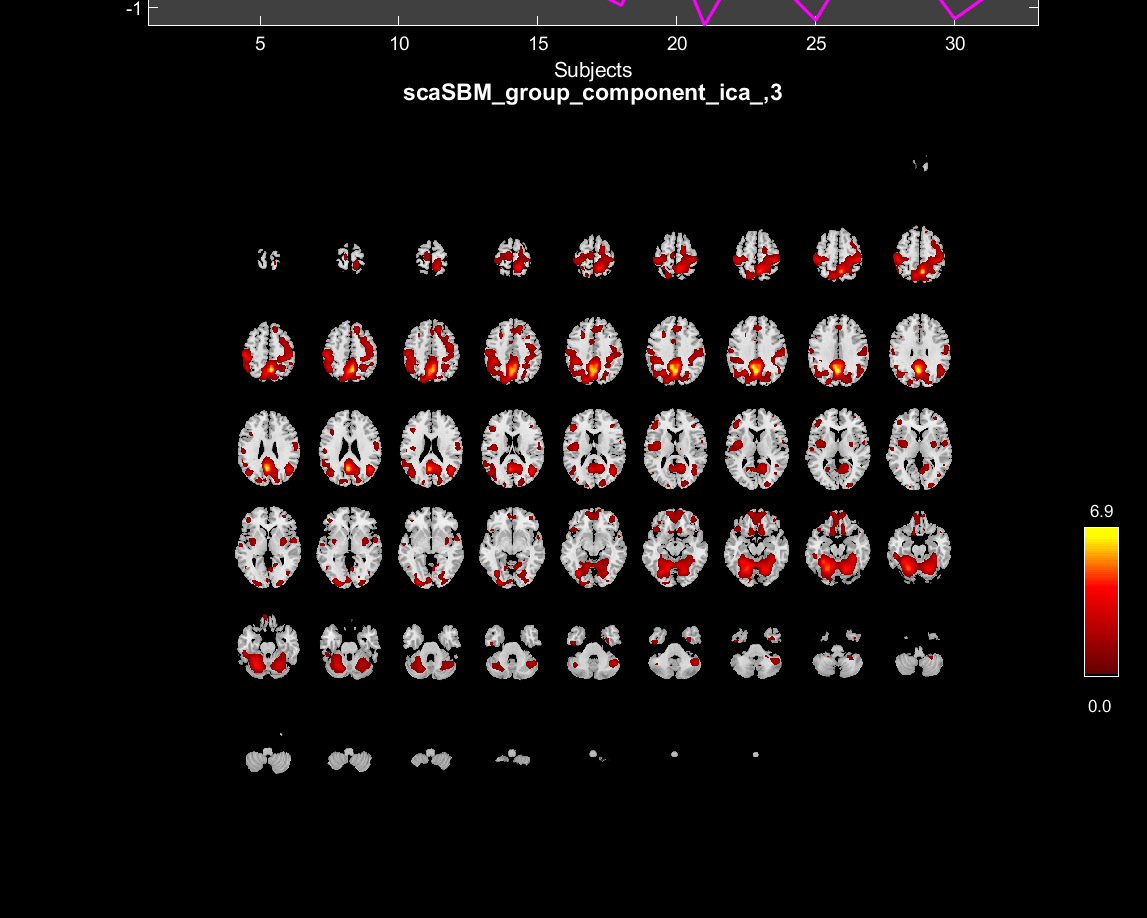

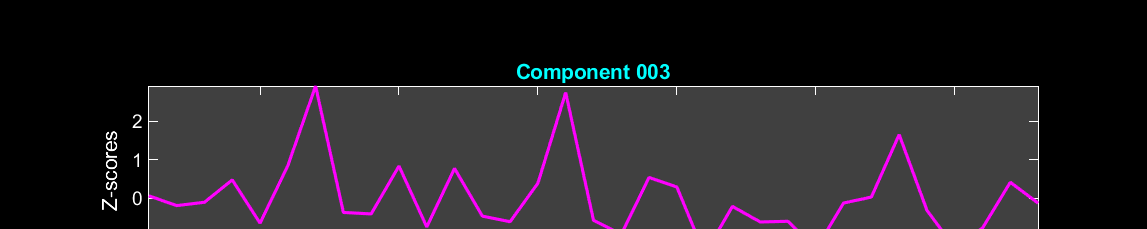

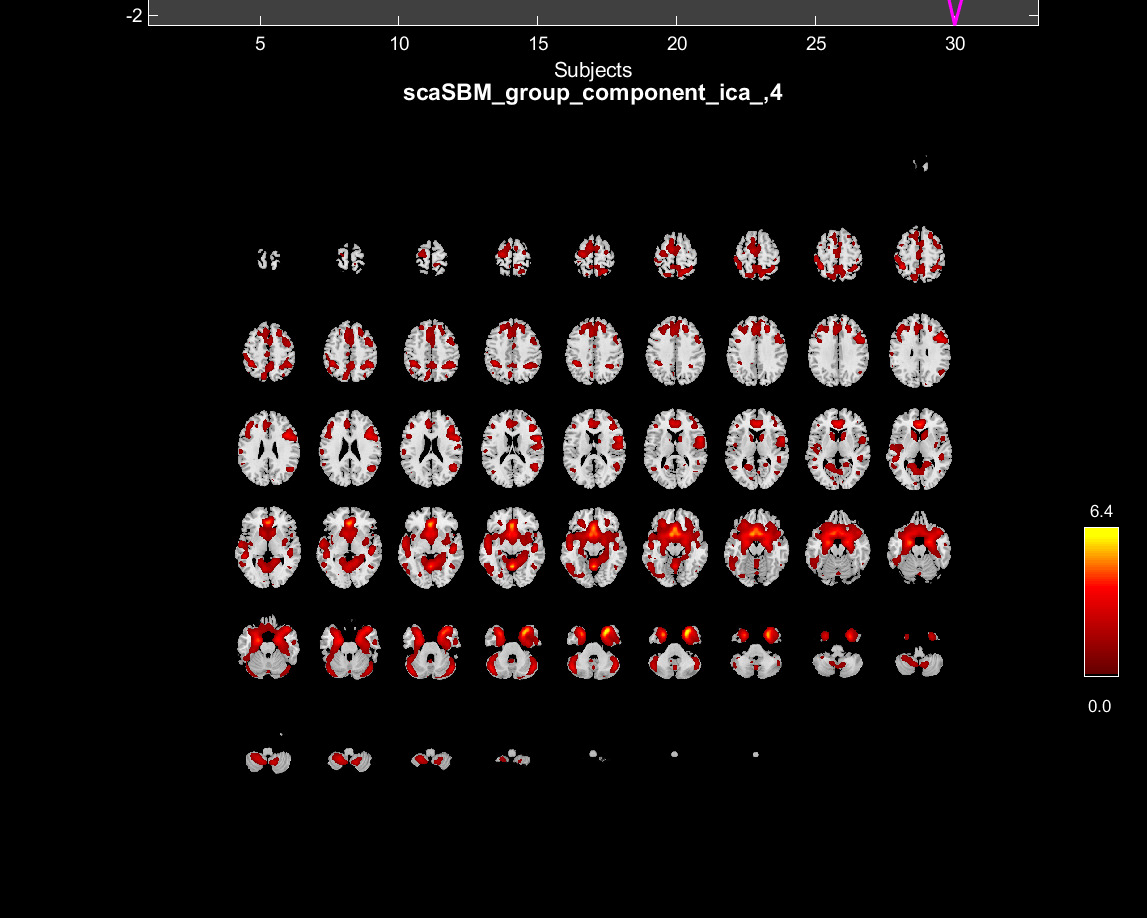

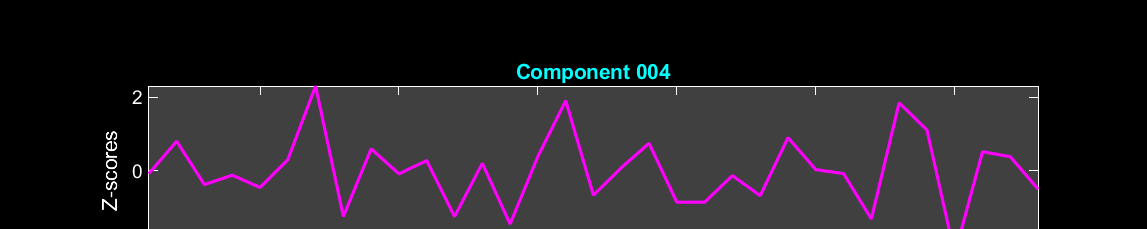

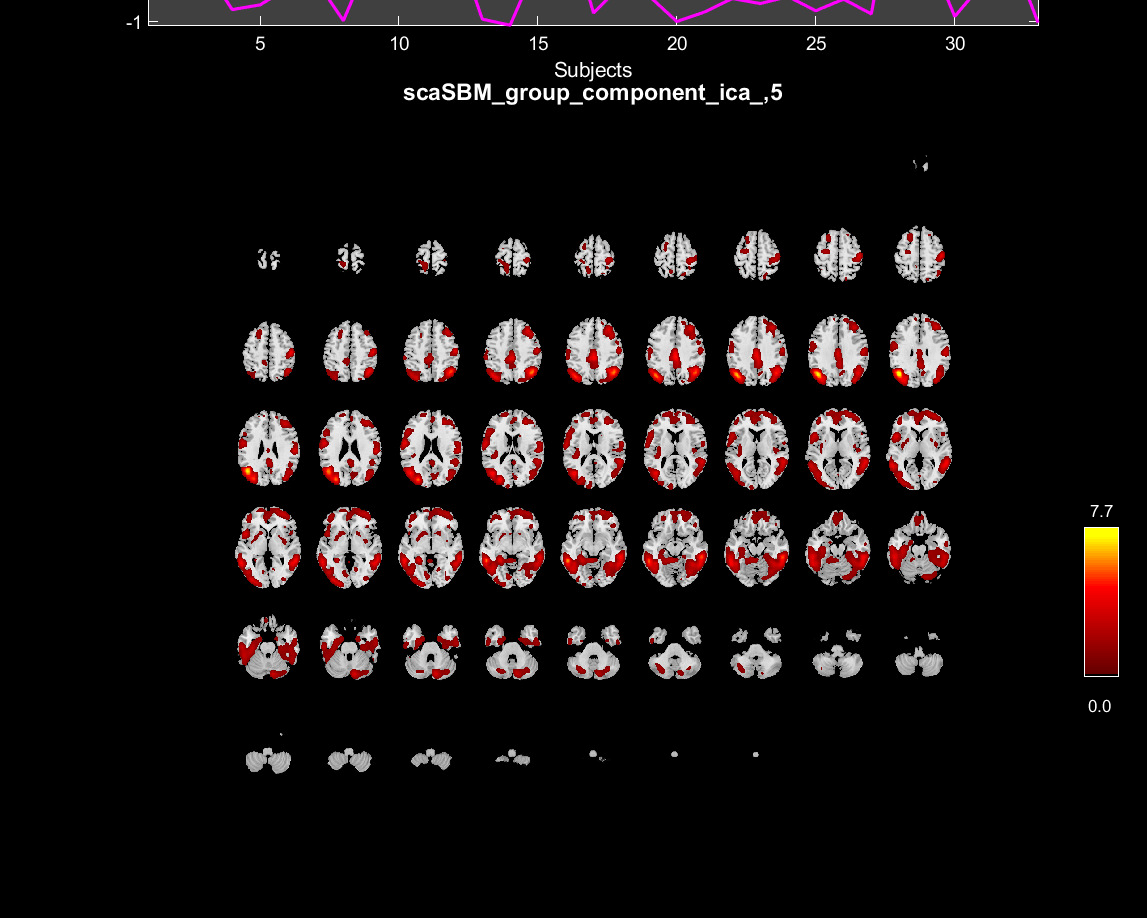

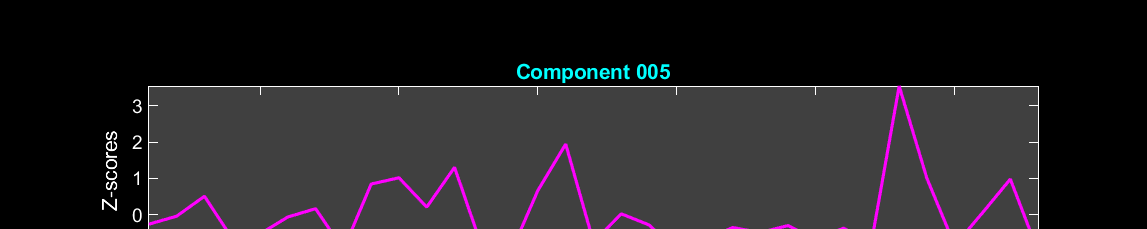

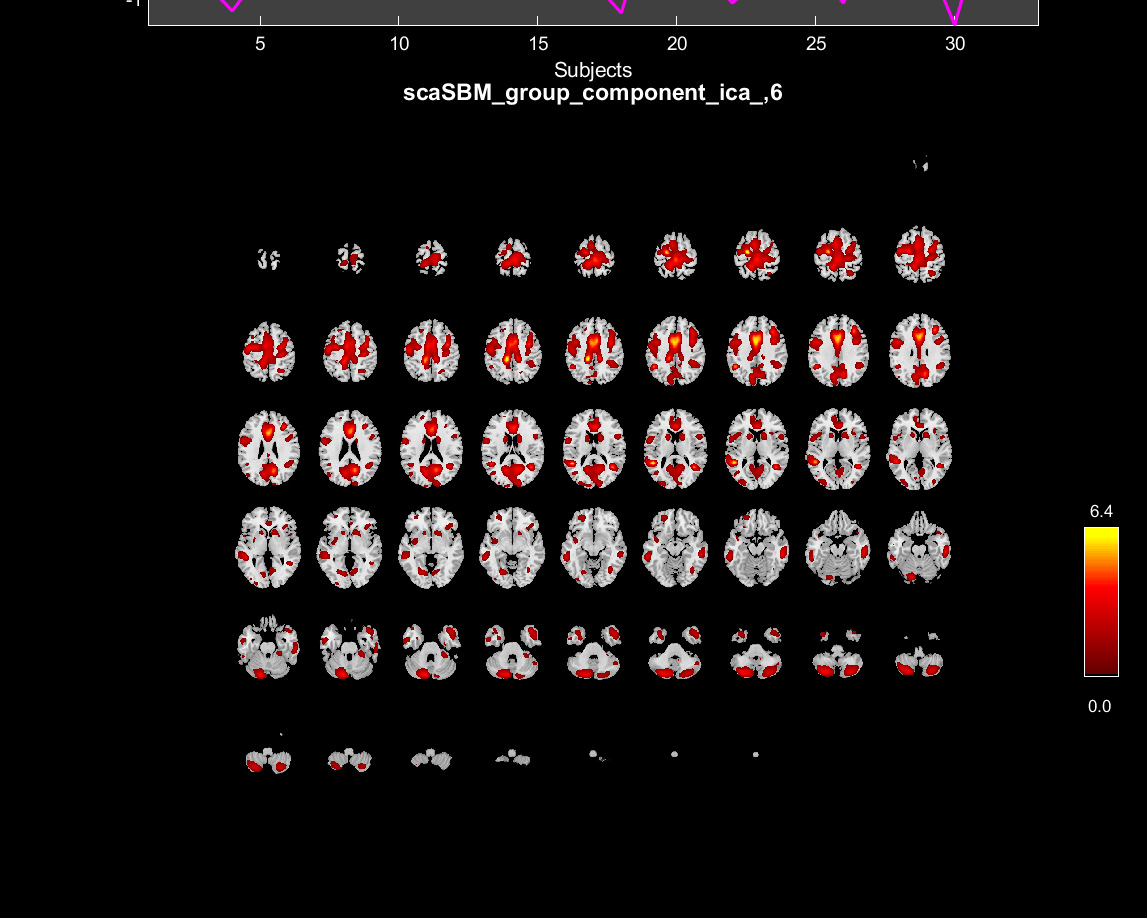

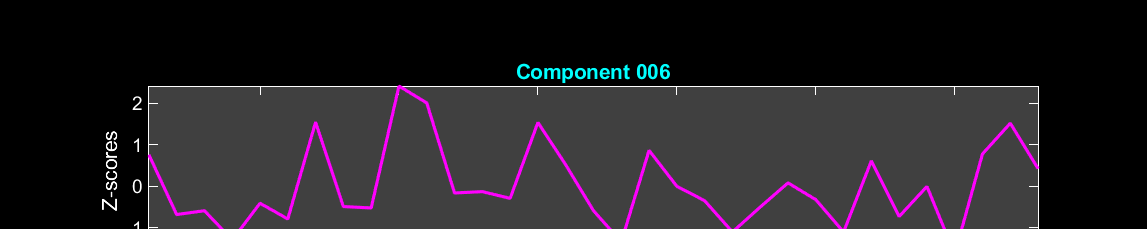

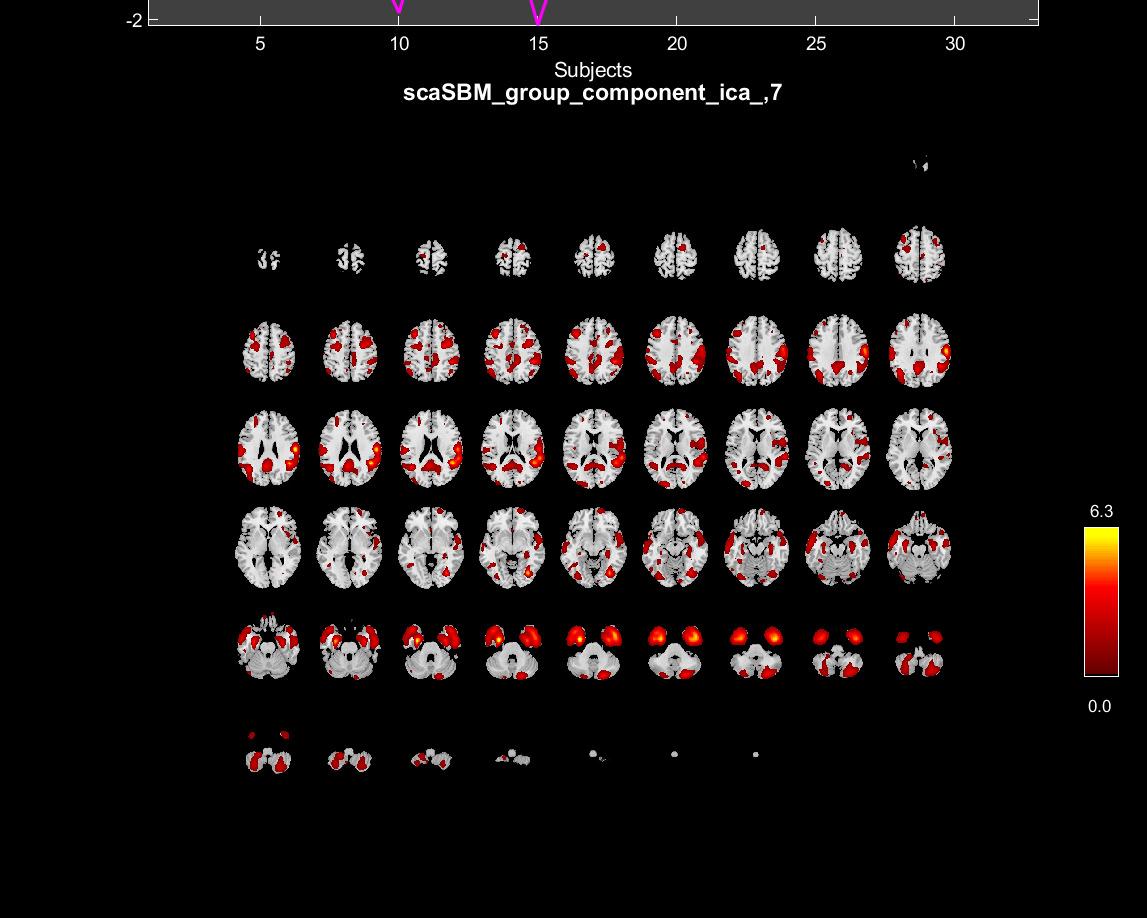

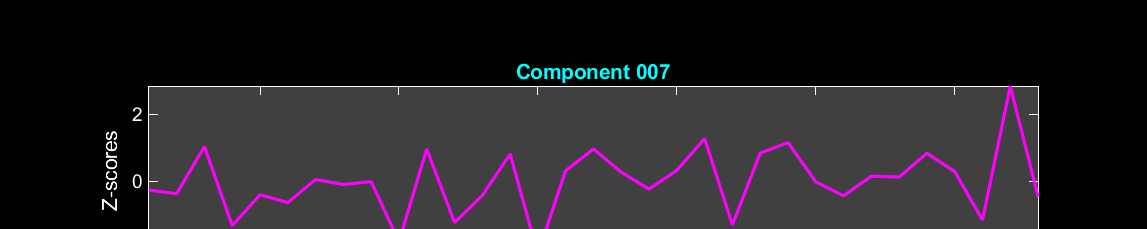

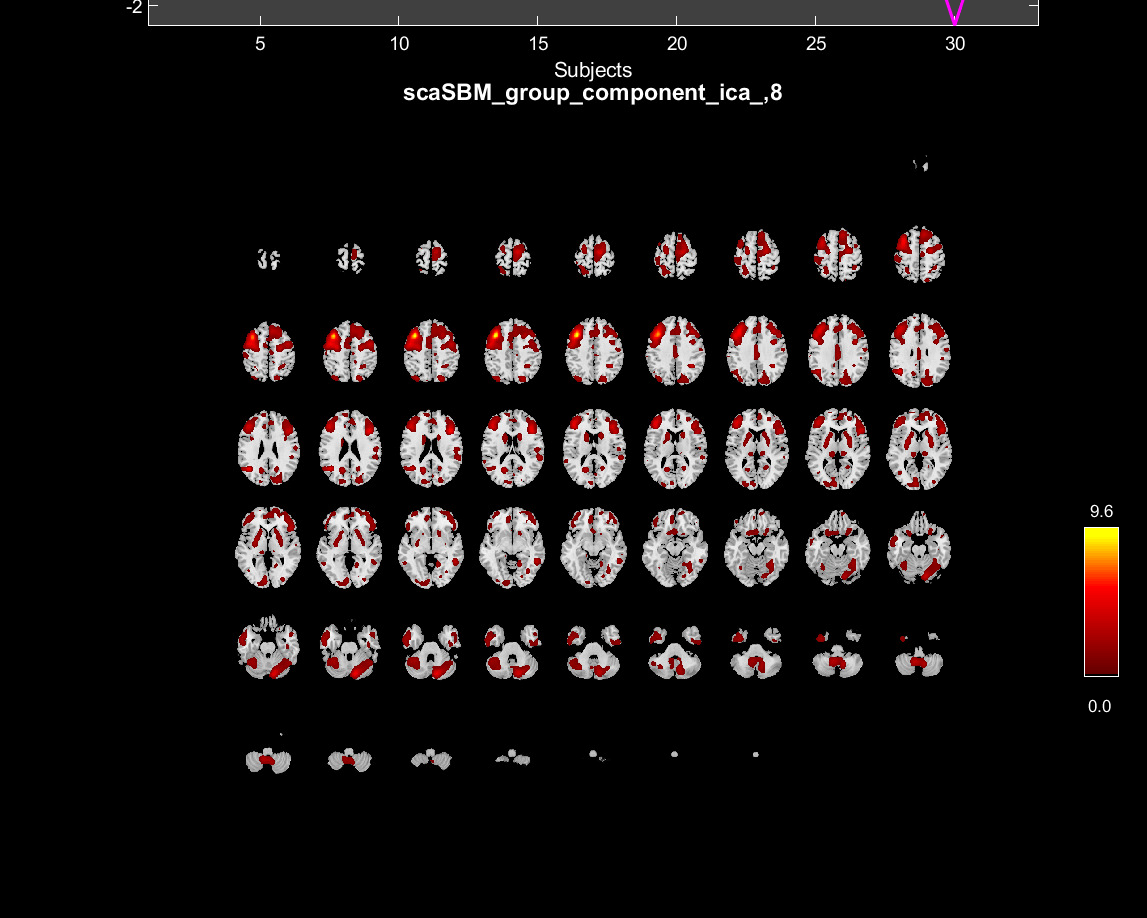

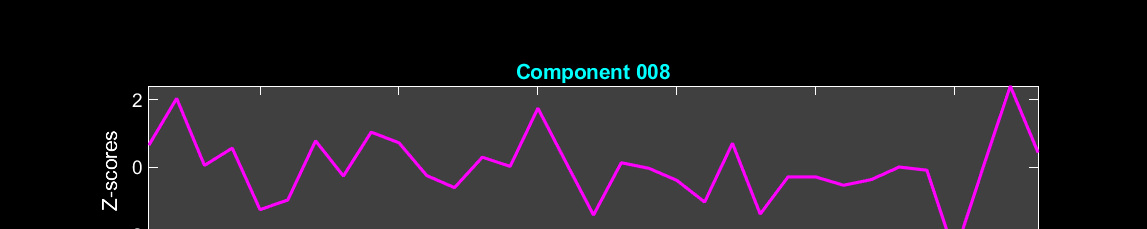

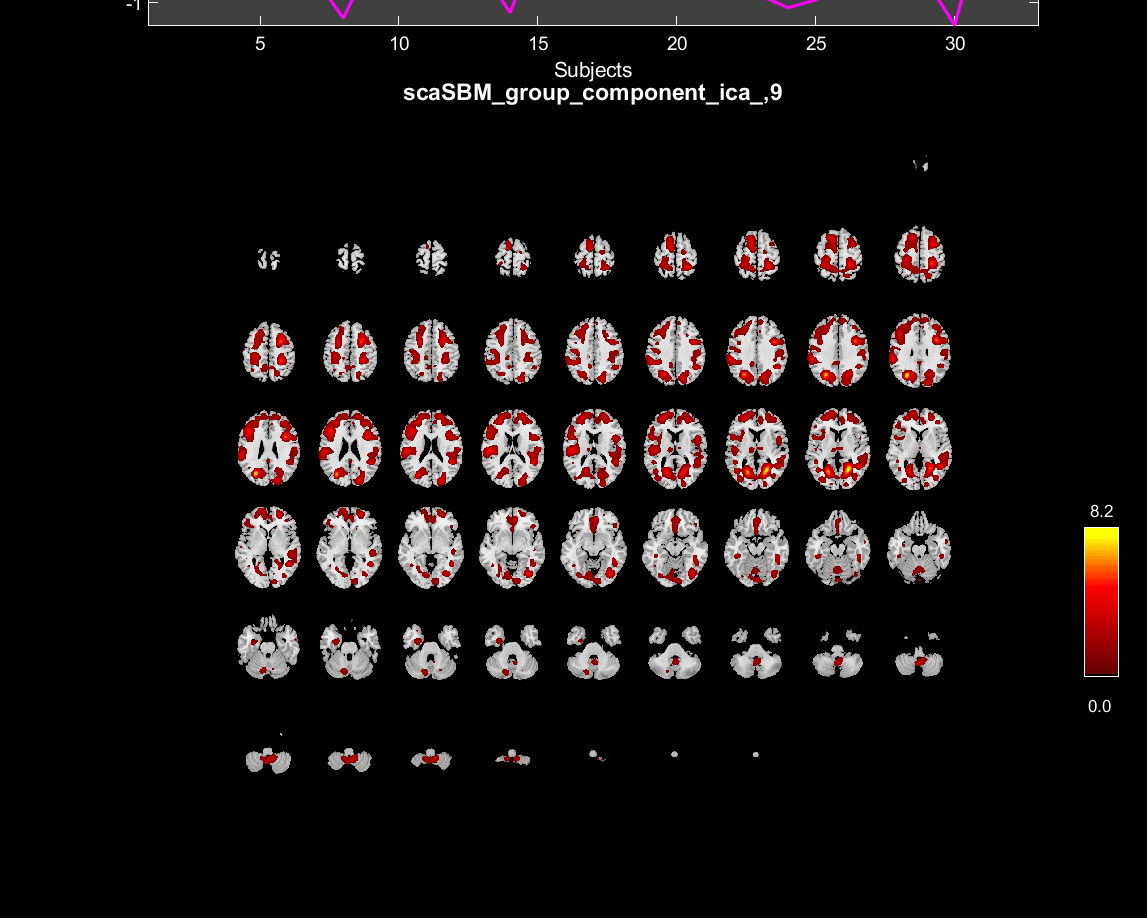

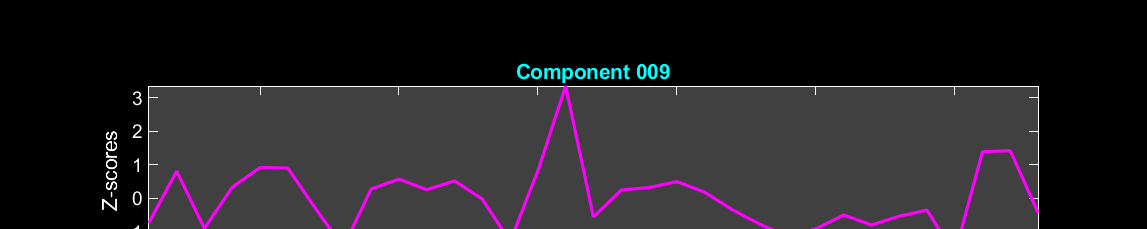

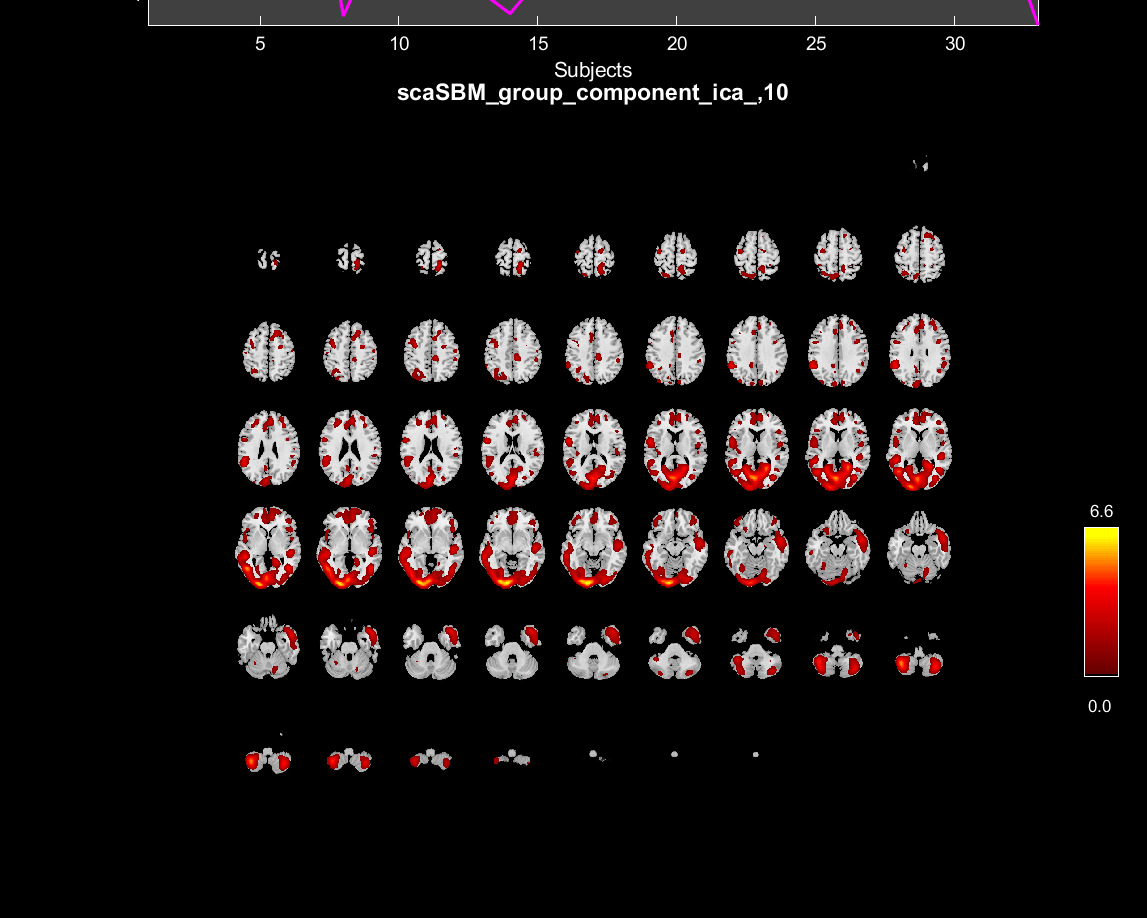

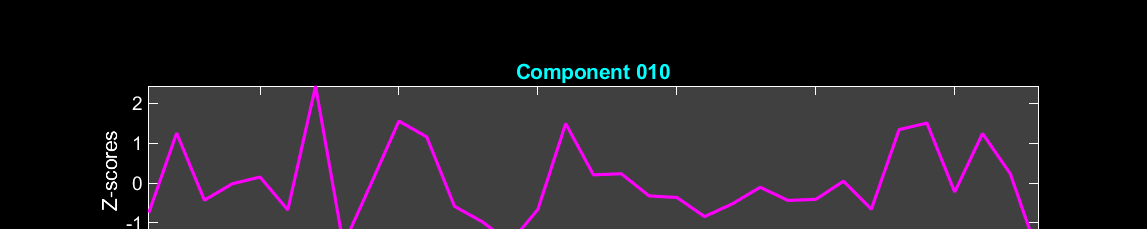

Supplement: Supplementary data 2 [file mmc2.docx]
