# Supplementary material for: A resting-state fMRI pattern of spinocerebellar ataxia type 3 and comparison with 18F-FDG PET
Source: Neuroimage Clin. 2022 Apr 25;34:103023. doi: 10.1016/j.nicl.2022.103023 (PMC9062756; doi:10.1016/j.nicl.2022.103023)
Supplement: Supplementary data 3 [file mmc3.docx]

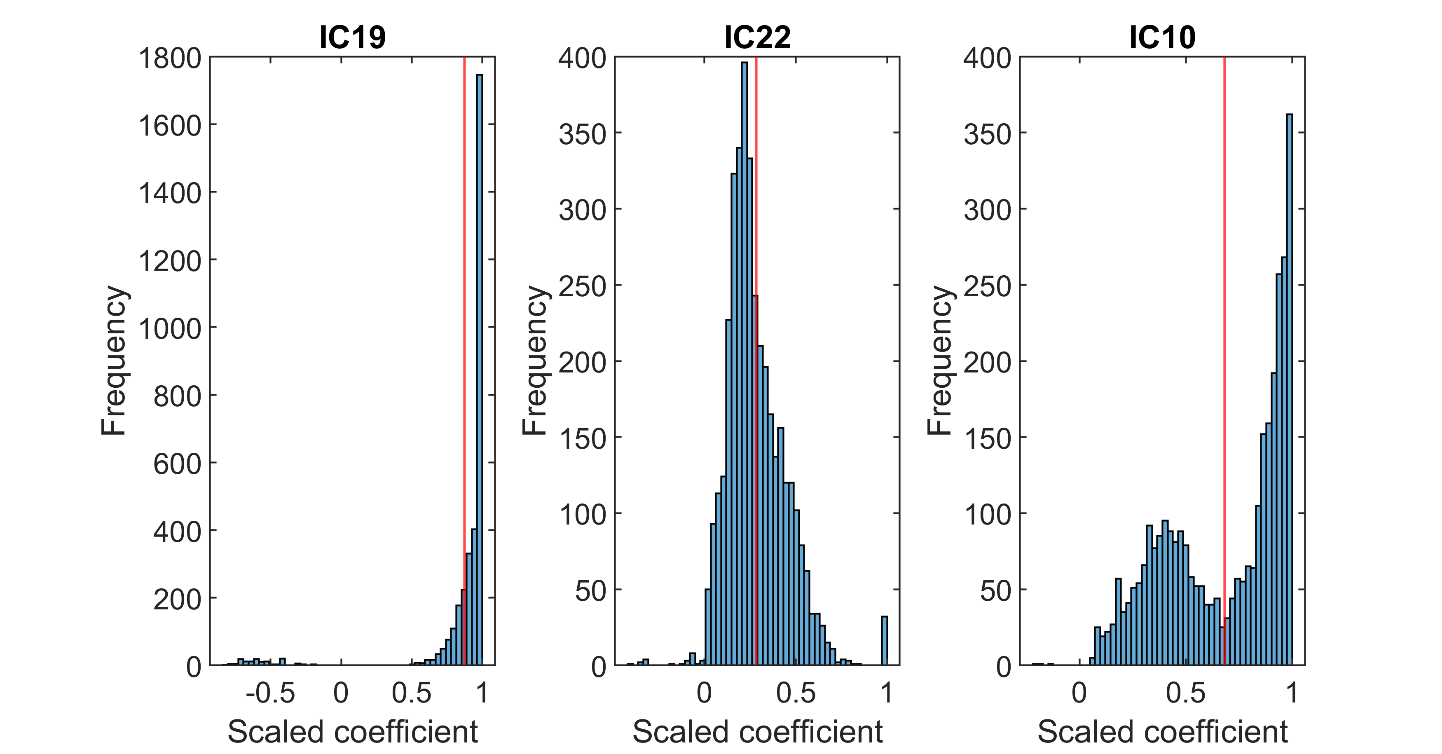


**Supplementary Figure 1:** Histograms for coefficients obtained with stepwise logistic regression with bootstrap resampling (5000 iterations). The mean is indicated with a red line.


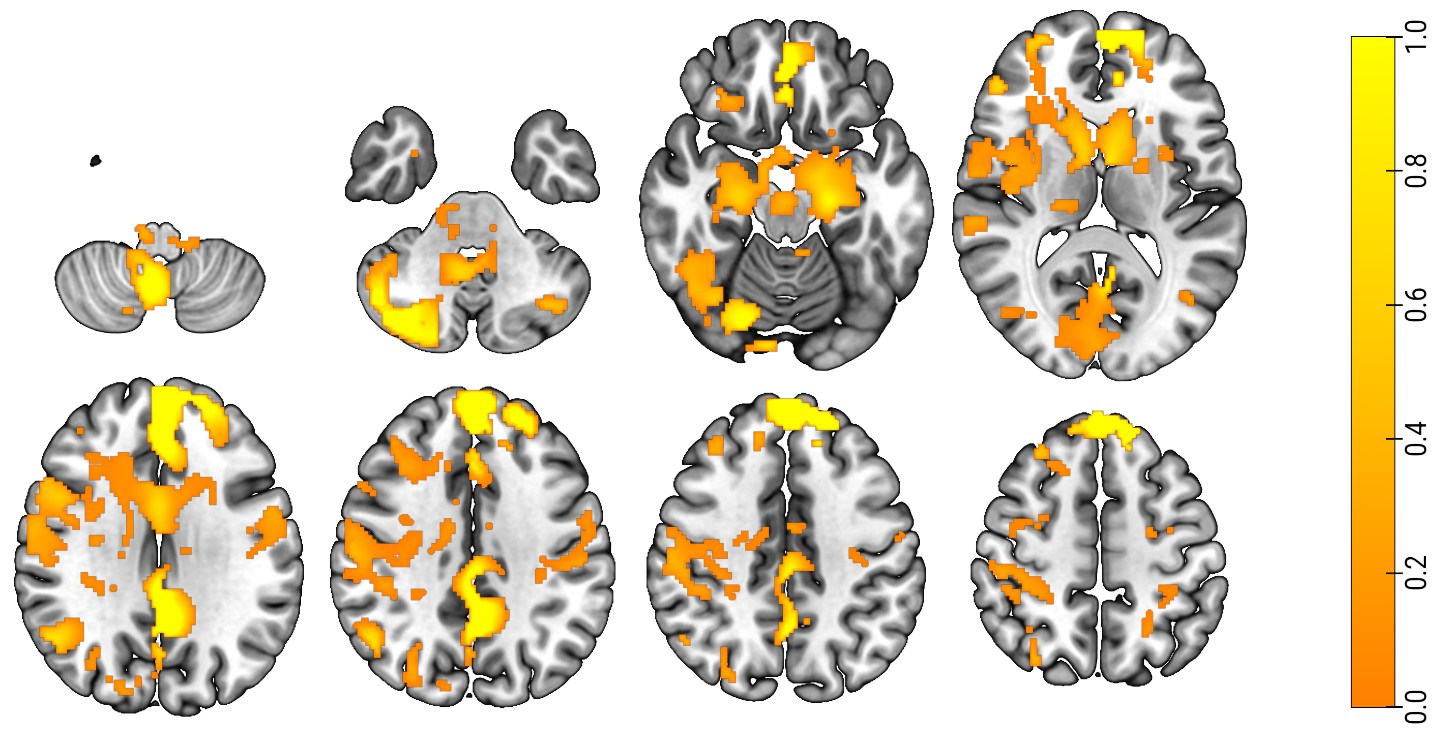


**Supplementary Figure 2:** Most stable (positive) voxels outside the 99% confidence interval. Colors reflect Z-scores.


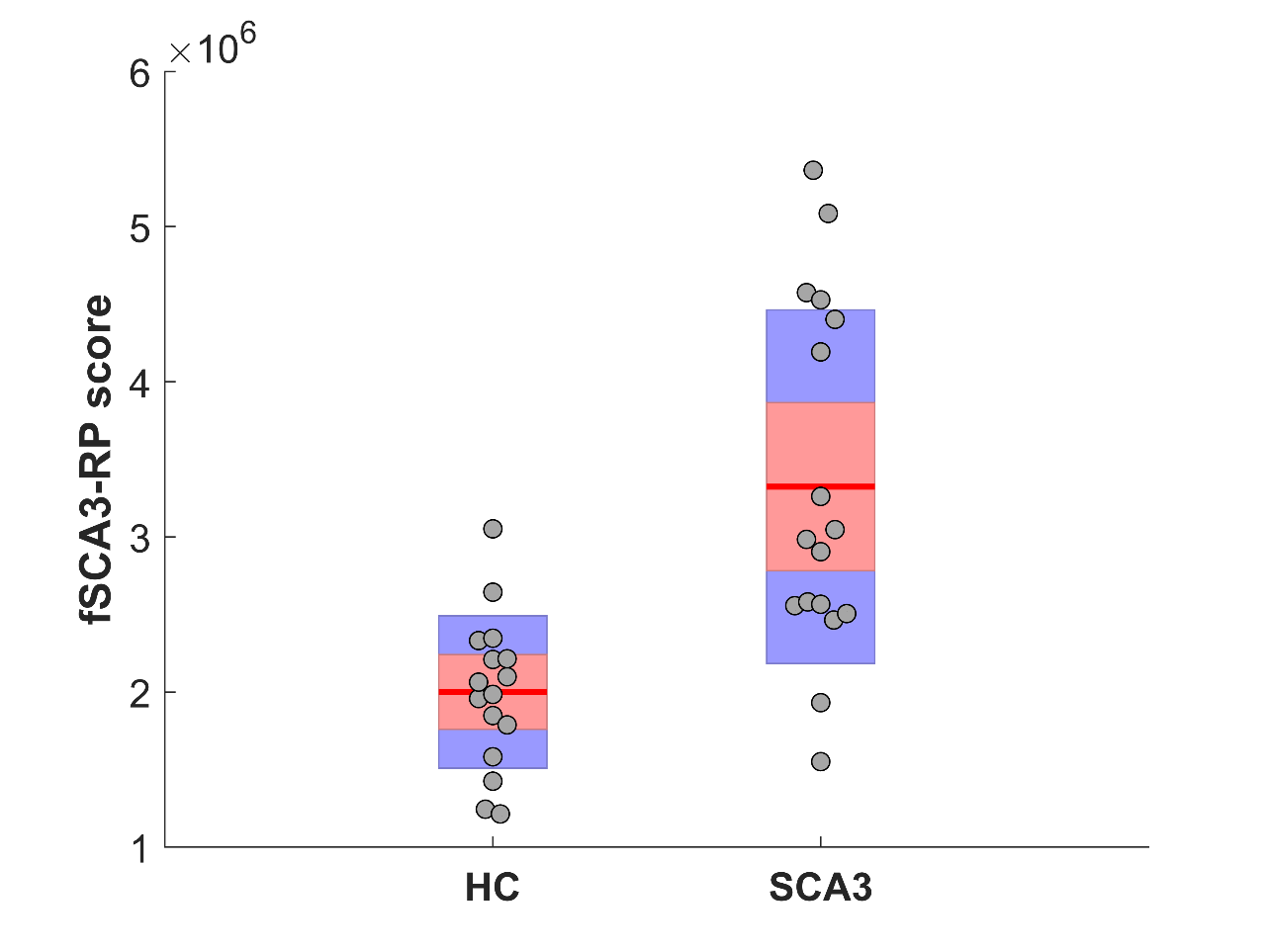


**Supplementary Figure 3:** Jittered data plot showing fSCA3-RP subject scores per group. Confidence interval is shown in red; ± 1 standard deviation is shown in blue.


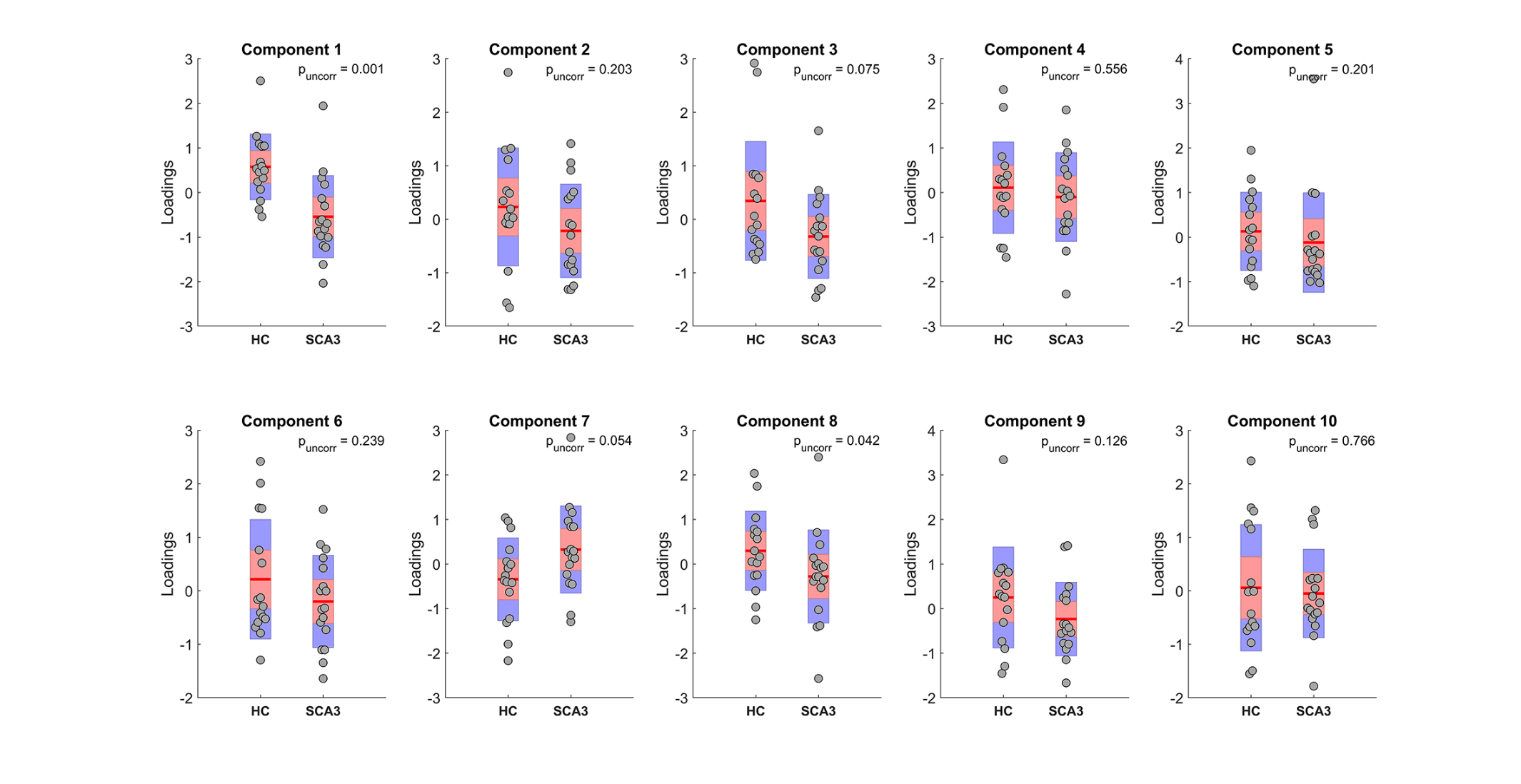


**Supplementary Figure 4:** Group differences in mixing coefficients of grey matter independent components.

**
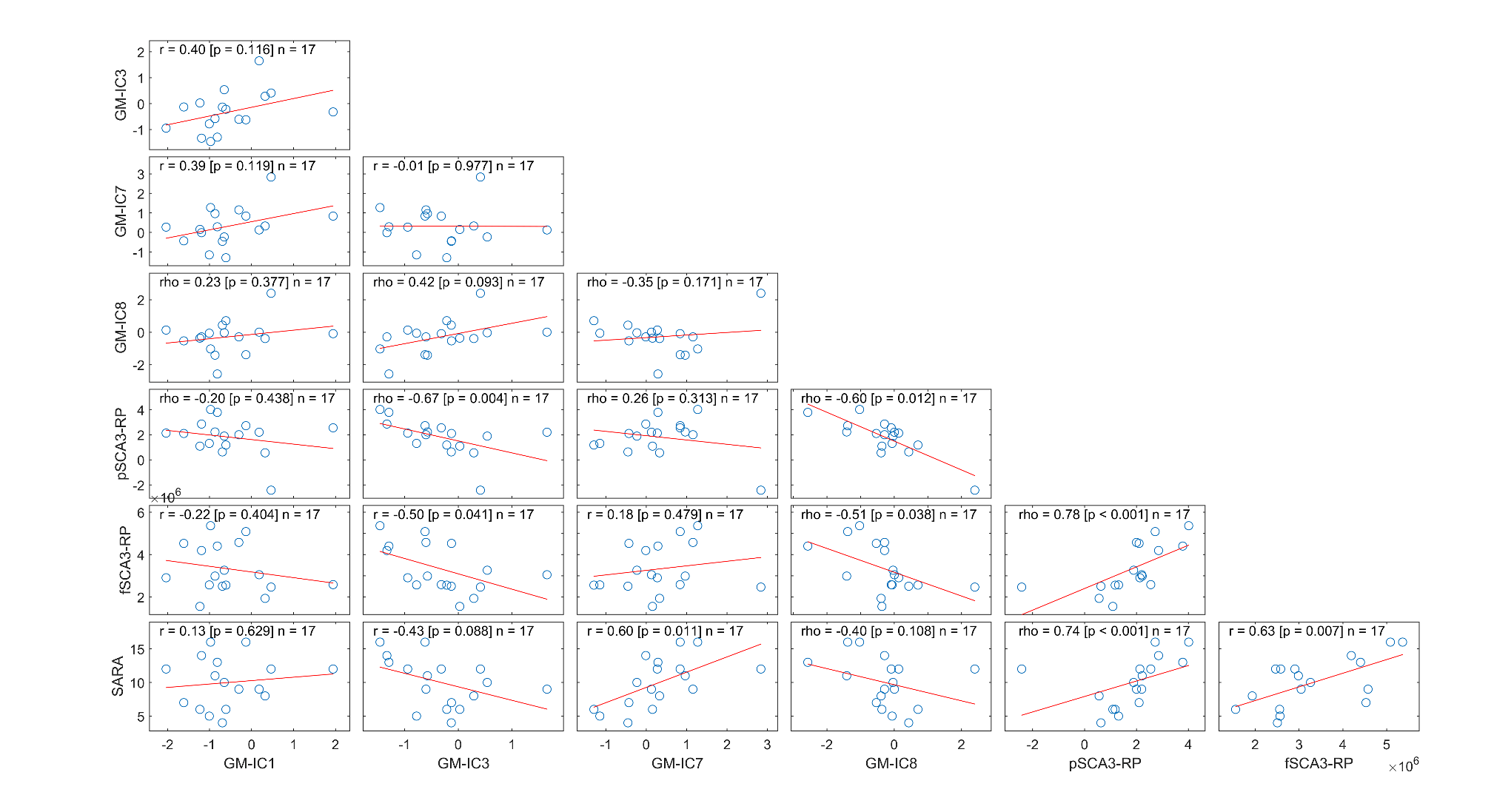
**

**Supplementary Figure 5:** Correlations between grey matter (GM) component mixing coefficients, pSCA3-RP, fSCA3-RP and/or SARA scores.
